# Comment on “A Tactical Medicine After-action Report of the San Bernardino Terrorist Incident”

**DOI:** 10.5811/westjem.2018.6.39216

**Published:** 2018-07-26

**Authors:** Daved van Stralen, Sean D. McKay, Kenji Inaba, Mark Hartwig, Thomas A. Mercer

**Affiliations:** * Loma Linda University School of Medicine, Department of Pediatrics, Loma Linda, California; † Element Rescue, Greenville, South Carolina; ‡ University of Southern California, Keck School of Medicine, Department of Surgical Critical Care, Los Angeles, California; § San Bernardino County Fire Protection District, San Bernardino, California; ¶ Strategic Reliability, Redlands, California

Editor:

We noted several consequential factual errors in the after-action report written by Bobko et al^1^ compared to the official after-action report of the San Bernardino terrorist incident.^2^

The authors^1^ state law enforcement transported two deceased victims to a hospital. Before establishment of Triage A, probation officers (PO) transported one survivor to a hospital emergency department.^2^ After establishment of Triage A, law enforcement officers (LEO) transported two victims from the Casualty Collection Point (CCP) to Triage A where paramedics determined death.^2^ To prevent further transport of deceased victims, the Special Weapons and Tactics’ (SWAT) medic wrapped tape around their wrists. No other deceased victims were moved from the scene.

The authors^1^ place the CCP on the other side of the building from the conference room. Figure 2 shows Triage A, not the CCP. The CCP was directly outside the conference room adjacent to Parkcenter Dr. South.^2^ This facilitated movement of victims from the conference room to the CCP, and then into law enforcement vehicles to Triage A.

The authors^1^ state SWAT medics did not operate in the medical branch and that duplication of medical resources occurred. After the conference room was cleared, the SWAT medic separated from the SWAT operation to provide patient care.^2^ Convergent POs and patrol LEOs initiated casualty treatment in the conference room and at the CCP under the direction of the SWAT medic. We found no law enforcement medical assets were dispatched. The fire department was the primary medical first responder and handled triage, treatment, and medical transport for victims.^3^ The Air Rescue (AR) helicopter responded as a law enforcement asset to a crime in progress. (The county communications center must dispatch all EMS aircraft).^2^ Two medics, SWAT and AR, rendered organized medical care in support of each other without duplication of efforts. Five minutes after LEOs first entered the conference room, LEOs and POs began evacuating victims to the CCP.^2^

The authors^1^ state a designated law enforcement medical director (LEMC) would streamline processes and enable the SWAT medic to focus on medical aid in the hot zone. In this incident, the early arrival of a SWAT medic was incidental to a nearby training exercise. The SWAT medic became the acting LEMC operating at the point of contact and did focus solely on providing medical care within the hot zone. He accomplished this with volunteer LEOs and POs who received rapid instruction for immediate medical aid and methods for patient carry to the CCP.^2^ These actions gave the SWAT medic time for the primary triage. The efforts of the AR medic enhanced patient treatment and assisted in secondary triage.^2^ In this environment characterized by volatility, uncertainty, complexity, ambiguity, and threat (VUCA-T),^4^ actions at the point of contact were more effective than streamlined processes. The first patient arrived at Triage A approximately 18 minutes after clearance of the room.^3^

The authors^1^ describe “delay in treating patients.” The deceased suffered non-survivable injuries from massive blood loss and extreme respiratory system or head injuries.^2^ The majority of gunshot wounds were in the head, chest, and abdomen of which none were amenable to citizen first aid. Environment and time are independent comorbidities, with nonlinear contributions to death, converting a “potentially survivable injury” into a non-survivable injury.^2^

A public safety incident appears disorganized and inefficient from a distance, yet it is a response to local, immediately available, imperfect information not detectable from farther away. This results in improvisation and nonlinear self-organization to local information.^2^,^5^ Top-down and bottom-up sensemaking,^6^ using cognitive and affective mental processes,^7^ gives direction.

Public safety personnel engage novel, unstructured problems in an unstructured environment. We urge caution when translating operational methods and thinking from the stable hospital environment to situations characterized by uncertainty, time-compression, and threat in a VUCA-T environment.^4^

There is more to an active shooter incident than medical care and law enforcement activity. We identified elements of high reliability organizing; interactive, real-time risk assessment and management; leadership *in extremis* with leader-leader constructs; proactive critical incident stress management; visual communication with heedful interrelating; and self-organizing improvisation.^2^

Victim extraction began five minutes after LEO entry, completed in 18 minutes. Within 18 minutes the fire department triaged, treated, and initiated transport for 14 patients, none of whom died. Our analysis identified different lessons learned.^2^

## Supplementary Information


