# Supplementary material for: Comment on “A Tactical Medicine After-action Report of the San Bernardino Terrorist Incident”
Source: West J Emerg Med. 2018 Jul 26;19(5):825–6. doi: 10.5811/westjem.2018.6.39216 (PMC6123096; doi:10.5811/westjem.2018.6.39216)
Supplement: Supplementary file 1 [file wjem-19-825-s001.pdf]

2018

strategicreliability, LLC

Making Better Decisions

Tactical Improvisation: After-Action/ Comprehensive Analysis of the Active Shooter Incident Response by the San Bernardino City Fire Department

DAVED VAN STRALEN, SEAN D. MCKAY, GEORGE T. WILLIAMS, AND THOMAS A. MERCER

SUBMITTED TO THE SAN BERNARDINO COUNTY FIRE PROTECTION DISTRICT

SUBMITTED BY STRATEGIC RELIABILITY, LLC, REDLANDS, CA

To the Responders on December 2, 2015,

Significant improvement and insight can be made, not only internally, but for other first responder agencies throughout our country when the voices of the responders are heard. Those who responded to the IRC building that day, did so with consummate courage and professionalism. The collective training, dedication, and immediate actions of these first responders undeniably saved lives. We are extremely grateful to the responders who volunteered to relive the emotional events of December 2, 2015, in an honest attempt to not only improve their response to future incidents, but to share these insights and lessons learned with other agencies across the nation, enhancing their response capabilities.

ACKNOWLEDGMENTS

Strategic Reliability, LLC

Element Rescue, LLC

Rescue Craft by Tactical Medical Solutions

Cutting Edge Training, LLC

ASSOCIATES

Najm Meshkati, PhD Viterbi School of Engineering, University School of Engineering

Yalda Khashe, PhD (c) Viterbi School of Engineering (c), University School of Engineering

Dennis Kowal, PhD Institute for Defense Analysis

Gary Provansal Division Chief San Bernardino County Fire Department (retired)

Steve Papenfuhs Sergeant, San Jose Police Department (retired)

Ryan Galdes SFC, 18D, USASOC

CONSULTANTS

Dominick Briganti Lieutenant, Clearwater Fire and Rescue

Matt Garrison MSG, 18Z, USASOC

Paul DiTuro MSG, 18D

Brendan Hartford Police Officer, Chicago Police Department, SWAT Team Medic, EMTP NRP., Secretary – Committee for Tactical Emergency Casualty Care

Eric Soderlund Detective, Pinellas County Sherriff's Office / Law Enforcement Correspondent with Imminent Threat Solutions

Ryan Mitchell Lieutenant, Firefighter, Paramedic, BA

Joe Paulino Chief of Police, San Bernardino City Unified School District Police Department

SURGICAL CONSULTANTS

Kenji Inaba, MD FACS, Surgical Critical Care, Keck School of Medicine, University of Southern California

John Agapian, MD FACS, Surgical Critical Care, Riverside University Health System - Medical Center

Charlie Hendra, RN BSN, Trauma Services, Riverside University Health System - Medical Center

Daniel Ludi, MD FACS, Trauma Surgery, Riverside University Health System - Medical Center

ADVISORS AND REVIEWERS

Captain Chesley "Sully" Sullenberger Author, Speaker, Safety Expert

Alex Alvarez MSG, 18D

Doug Chadwick Deputy Chief, Whatcom County Sheriff's Office, Bellingham, WA.

Alan Hester VP, Tactical Medical Solutions, Former 18D

Rodney Johnson Lieutenant, Clearwater Police Department, SWAT Team Leader

Julie McKay Managing Partner, Element Rescue, LLC

Chris Miller CHSE, MICP, MSCP, Director of Operations, Center for Medical Simulation and Research, University of California, Riverside, School of Medicine

Randy Paige CBS investigative reporter

Jeff Parks Undersheriff, Whatcom County Sheriff's Office, Bellingham, WA.

Gina Piazza DO, FACEP, Associate Professor of Emergency Medicine at the Medical College of Georgia

Kathy Platoni Psy.D. DAAPM, FAIS, Clinical Psychologist, COL (RET), US Army, COL, Ohio Military Reserve

Andrew Schrader P.E. Recon Response Engineering, LLC

SAN BERNARDINO COUNTY FIRE PROTECTION DISTRICT

Carly Crews RN, BSN, MICN

Tim Porter Assistant Chief

APPRECIATION FOR SUPPORT FROM

Deputy Chief Scott Webster, Captain Mark Kittelson, Captain Evan Hannah, and Captain Brian Larsen of Clark County Fire Department, Route 91 Task Force. Your insights, comments, and discussions concerning the San Bernardino incident and the recent attack your department responded to was invaluable.

AUTHOR BIOGRAPHIES

Daved van Stralen, MD, FAAP Dr. van Stralen is a principal of Strategic Reliability. He assisted in creating the PICU and the pediatric critical care transport service, Loma Linda University Medical Center, and the Emergency Medical Care bachelor's degree, Loma Linda University. He is an Assistant Professor of Pediatrics, Loma Linda University Children's Hospital and Children's Subacute Center at Community Hospital of San Bernardino (California). Dr. van Stralen has been Medical Director for American Medical Response, San Bernardino County, the San Bernardino County Fire Department, and the Riverside County EMS Agency. In the 1970's he worked in South Los Angeles as an ambulance man and fire department rescue ambulance driver, Los Angeles City Fire Department.

Thomas A. Mercer, RAdm, USN (retired) RAdm Mercer is a principal of Strategic Reliability. He was Captain of the USS Carl Vinson (CVN 70), studied by UC Berkeley High Reliability Organization (HRO) faculty. He flew 255 Vietnam Combat Missions, has 970 carrier arrested landings, and has 3,700 hours in the A-4C and A-7E. RAdm Mercer was commander of Subic Bay Navy Base when the Mt. Pinatubo eruption during a typhoon threatened Clark Air Base. During his operational squadron tours, RAdm Mercer was detailed as the Aviation Safety Officer and the Aircraft Maintenance Officer. He was the principle investigator for eight major aircraft accidents and the author of numerous incident reports improving the reliability and correcting material deficiencies of new fleet aircraft. He had three combat deployments to Vietnam (USS Franklin D. Roosevelt, CVA 42, and USS Kitty Hawk, CVA 63).

Sean D. McKay Mr. McKay works with Rescue Craft by Tactical Medical Solutions and is the Director of Disruptive Rescue & Austere Medicine with Element Rescue, LLC. Mr. McKay served as a firefighter/paramedic and SWAT Rescue-Medic Team Leader on the West Coast of Florida until December 2006. He currently develops and conducts training in operational rescue and austere medicine for USSOCOM, federal agencies, and tactical municipality teams. Sean also sits on the Executive Board of the Committee for Tactical Emergency Casualty Care.

George T. Williams Mr. Williams is the Director of Training for Cutting Edge Training, LLC, in Bellingham, Washington. He is a Training Specialist and certified police Master Instructor. He has diverse experience in police training, emergency management training and response, Emergency Operation Center operations, and terrorism response. He also serves as an expert witness in federal and state courts throughout the nation in police force response, practices, and policy. He is a published author of two books and more than 300 articles related generally to policing practices, tactics, or skills.

RECOMMENDED CITATION:

van Stralen, Daved, Sean McKay, George T. Williams, Thomas A. Mercer. 2017. *Tactical Improvisation: After-Action/ Comprehensive Analysis of the Active Shooter Incident Response by the San Bernardino City Fire Department December 2, 2015.* San Bernardino, CA: San Bernardino County Fire Protection District.

Published 2018

TABLE OF CONTENTS

Memo From the San Bernardino County Fire Protection District ....- 7 -

Executive Summary ....- 8 -
Supporting Resources .... - 9 -
AAR Findings .... - 10 -
What Worked and Why .... - 10 -
Gap Analysis .... - 11 -

Abbreviations, Definitions, and Figures ....- 13 -
Abbreviations .... - 13 -
Definitions .... - 14 -
Figures .... - 15 -

Introduction ....- 17 -

Purpose and Scope of this Report ....- 19 -

Factual Narrative ....- 20 -
The Shooting and Law Enforcement (LE) Response .... - 20 -
Rescue and Extraction .... - 21 -
Fire Response, Triage and Treatment .... - 22 -

Methodology ....- 28 -
The AAR Team .... - 28 -
Methods .... - 28 -
Medical Information .... - 28 -
Materials .... - 29 -
Literature Review .... - 30 -
Data Evaluation .... - 30 -
Time Segments: Clinical and Operational .... - 32 -

Data ....- 34 -
Injuries and Illness .... - 34 -
Level of Triage .... - 35 -
Wounding Pattern .... - 36 -
Time Lines .... - 37 -

Analysis: Time Line ....- 38 -
Multiple Time Lines in an Active Shooter Incident .... - 38 -
Considerations .... - 39 -

Gap Analysis: Local Standards ....- 40 -
San Bernardino City Fire Department Standards .... - 40 -
ICEMA Standards .... - 40 -
San Bernardino City Fire Department Standards .... - 43 -
Key ICS Positions .... - 43 -
ICEMA Standards for Fire Department Multi-Casualty Incidents .... - 44 -
8070 Aircraft Rotation Policy .... - 44 -
EMS Regulatory Obligations .... - 44 -

Gap Analysis: National Strategies for Patient Care Response During Intentional Mass Casualty Events .... - 46 -
SBFD Response .... - 46 -
Hartford Consensus and Context .... - 46 -
Military Wounding Patterns vs. Civilian Wounding Patterns .... - 47 -
Findings: Overview of Methods and Data .... - 50 -
Wounding pattern .... - 50 -
Patient movement .... - 51 -
Triage .... - 52 -
Injury Discrepancy: Field to Emergency Department .... - 53 -
Treatment .... - 54 -
Times .... - 56 -
Working with Law Enforcement .... - 56 -
Threat, Cognition, and Affect .... - 57 -
CISM/ CISD .... - 57 -
Leadership *in extremis* (in dangerous contexts) .... - 58 -
Public Safety Disciplines .... - 58 -
Communication .... - 59 -
Analysis .... - 60 -
Introduction: Concepts for this Active Shooter Incident .... - 60 -
Emergency medical care in the public safety environment .... - 61 -
THE VUCA-T Environment and Self-Organizing Improvisation .... - 62 -
High Reliability Organizing and Public Safety Disciplines .... - 68 -
Threat, Cognition, and Affect .... - 71 -
Expert Training and Performance .... - 77 -
Leadership .... - 80 -
Leadership in action .... - 84 -
Triage and Survivability .... - 87 -
Working with Law Enforcement .... - 96 -
Critical decision .... - 97 -
Incident Command System (ICS) .... - 98 -
Communication .... - 101 -
News Media .... - 105 -
Beyond imagination .... - 108 -
Gap between Fire and Police Command .... - 111 -
References .... - 114 -

SAN BERNARDINO COUNTY FIRE PROTECTION DISTRICT

157 W. 5th Street, 2nd Floor ● San Bernardino, CA 92415-0451 ● (909) 387-5974 ● Fax (909) 387-5542

Administrative Headquarters

Mark A. Hartwig
Fire Chief/Fire Warden

Don Trapp
Deputy Chief

John Chamberlin
Deputy Chief

Proudly Serving:
City of Adelanto
City of Fontana
City of Grand Terrace
City of Hesperia
City of Victorville
Town of Yucca Valley

And the Communities of:
Amboy
Angelus Oaks
Baker
Baldwin Lake
Baldy Mesa
Barton Flats
Big River
Black Meadow Landing
Bloomington
Blue Jay
Cedar Glen
Crestline
Deer Lodge Park
Devore
El Mirage
Fawnskin
Forest Falls
Green Valley Lake
Harvard
Havasu Landing
Helendale
Hinkley
Johnson Valley
Joshua Tree
Lake Arrowhead
Landers
Lucerne Valley
Ludlow
Lytle Creek
Mentone
Mt. Baldy
Mt. Home Village
Mt. Pass
Mt. View Acres
Muscoy
Needles
Oak Hills
Oro Grande
Park Moabi
Phelan
Pinon Hills
Pioneer Town
Red Mountain
San Antonio Heights
San Bernardino
Searles Valley/Trona
Sky Forest
Spring Valley Lake
Summit Valley
Twenty-Nine Palms
Twin Peaks
Windy Acres
Wonder Valley
Wrightwood

May 8, 2018

Re: San Bernardino City IRC Incident that occurred on December 2, 2015

The IRC incident that occurred in San Bernardino, CA on December 2, 2015 was a tragic incident that impacted many individuals and their families. The incident also impacted the City of San Bernardino as well as surrounding communities. Families and communities have been changed forever.

San Bernardino County Fire Protection District would like to honor the victims and families that have been impacted by this incident. We understand that as time passes the pain may never go away. For many of you the scars from this incident will last a lifetime. Our hope is that this report will answer some of your questions as well as offer some closing for you. Many of you reading this report are heroes. Heroes that possibly protected victims, assisted with evacuating the facility, or maybe treated injuries as best you could. Thank you for your assistance.

We would also like to recognize San Bernardino City Fire Department as well as surrounding departments that were involved in this incident. San Bernardino City Fire Department acted in a calm, professional manner from the beginning of the incident into the following days. The actions that you took during the incident no doubt saved many lives. Many departments provided automatic aid into San Bernardino City during and after the incident. This ensured support for the IRC incident as well as provided Fire and EMS coverage in San Bernardino City.

The facts for this incident were gathered and reviewed over many months. Meetings, discussions and interviews were held to confirm that this report is as accurate as possible. San Bernardino County Fire Protection District has thoroughly reviewed the documentation contained in this report and approves its release.

Respectfully,

Mark Hartwig, Fire Chief

San Bernardino County Fire Protection District

BOARD OF SUPERVISORS

Robert A. Lovingood	Janice Rutherford	James Ramos	Curt Hagman<br>Vice-Chairman	Josie Gonzales	Gary McBride
Vice Chair, First District	Second District	Chair, Third District	Fourth District	Fifth District	Chief Executive Officer

EXECUTIVE SUMMARY

On December 2, 2015, the San Bernardino County Environmental Health Department conducted a training program in a conference room of the Inland Regional Center (IRC) located in San Bernardino, California. Shortly before 1100 local time, two assailants dressed in black, later identified as Syed Rizwan Farook (the male assailant) and his wife, Tashfeen Malik (the female assailant), approached the conference room with semiautomatic rifles. Outside the exterior doors they shot five individuals, killing three of them. They then entered the conference room and fired upon the attendees. Within minutes, the two assailants shot 36 attendees, killing 14 of them.

A San Bernardino Police Department (SBPD) SWAT Medic, who was also a San Bernardino City Fire Department (SBFD) firefighter paramedic, entered the building as part of the SWAT Team. After the initial clearing of his designated area, he broke off from the SWAT Team and transitioned to patient care. The SWAT Team and other Law Enforcement (LE) officers cleared the upper floors of the building. Law Enforcement (LE) officers from police departments and the county probation office assisted the SWAT Medic and removed casualties to the casualty collection point outside the conference room. These LE officers also acquired police and civilian vehicles to transport casualties from the casualty collection points to the fire department triage and treatment area.

The initial SBFD response included four companies (three engine companies and one truck company), three ambulances, and one battalion chief. Two additional companies, upon hearing the initial assignment, volunteered to respond. A fire dispatcher self-initiated a second alarm just before the responding Battalion Chief requested the same assignment. This action brought an additional nine units to the scene. Fire companies staged at Waterman and Orange Show Road. The LE command post (CP) was at the north side of the IRC building initially but soon changed to a site north, at Waterman Ave. and Orange Show Rd.

While in staging, the fire battalion chief (BC) assigned predesignated duties for medical communication, triage, treatment, transportation, and staging. The medic engine company assigned to medical communication (MedComm) notified the EMS system (ReddiNet) of an active shooter incident with the possibility of 20 victims. ReddiNet began polling area hospitals for capacity and bed availability. A second medic engine company notified Loma Linda University Medical Center of the incident. An LE officer at Triage A waved the fire companies in but, without radio confirmation, they were held in staging. Radio incompatibility between the San Bernardino City Fire Department and the San Bernardino Police Department prevented the LE Incident Commander (IC) from communicating the order for fire/EMS personnel to move to Triage A.

As fire department IC (FD IC), the Fire Battalion Chief drove toward the CP to form a unified command (UC) with the LE IC. Parked police vehicles blocked his access to the CP. For the same reason, fire companies hand carried life-saving equipment several hundred yards to Triage A. One company, risking entrapment of their vehicle, drove the same distance against oncoming traffic.

The Tactical Command Post was positioned just north of the IRC building at the intersection of Waterman and Parkcenter Drive North. Unified Command (UC) with FD IC and PD IC formed early by radio, assisted by the Emergency Manager from the San Bernardino City Unified School District (SBCUSD) PD. Physical UC occurred later. San Bernardino County Fire Department responded by their duty officer for liaison. A Type 3 Incident Management Team (IMT) was requested for logistic support.

Triage A was set up with salvage tarps placed on the ground. Chalk was used to mark the tarps “Immediate,” “Delayed,” “Minor,” and “Deceased.” A fire engine blocked the view of the deceased tarp from the area where

evacuees would be placed. While Triage A was being set up, an LE officer arrived to advise they were bringing patients out soon. Almost immediately patients arrived on foot and in LE driven vehicles.

SBFD paramedics and fire officers, assisted by San Manuel Fire Department mutual aid, also treated patients and assisted evacuees at Triage B. This also occurred later on at the golf course, and at SBFD Fire Station 231 (FS 231) after Triage A was moved due to the discovery of a suspicious device. Several other incidents associated with the original incident occurred as well, to which SBFD responded. One Rescue Task Force (RTF) with an embedded Terrorism Liaison Officer (TLO) responded for mutual aid from Rancho Cucamonga Fire Department and SBFD also created an ad hoc RTF.

SUPPORTING RESOURCES

SBPD, SBCUSD police, and San Bernardino County Probation Officers provided force protection to fire personnel and apparatus. One incident of a police shootout deprived SBFD of force protection at FS 231 for a period of time.

Fire Department Chaplains from throughout San Bernardino and Riverside Counties began responding 90 minutes after the first fire apparatus responded. Other services began responding and Critical Incident Stress Management became available for first responders.

American Medical Response sent supervisors and began staging. Symons Ambulance responded with two ambulance strike teams with a physician-led team of nurses, paramedics, and EMTs. Inland Counties Emergency Agency initiated emergency operations, notifying the California State Medical Health Operational Area Coordination (MHOAC). Riverside County EMS Agency heard about the call and surveyed Riverside County for the security of hospital and EMS resources.

Burlington, Northern, and Santa Fe Railroad were notified of the need for track closure approximately three hours after the incident started.

SBCUSD PD and SBCoSO brought in buses to evacuate people from IRC to Rock Church.

San Manuel Fire Department, Colton Fire Department, San Bernardino County Fire Protection District and Rialto Fire Department provided mutual aid. American Medical Response, Symon's Ambulance, and Sheriff's Air Rescue provided EMS support.

Patients were transported to Loma Linda University Medical Center Trauma Center, Arrowhead Regional Medical Center Trauma Center, Riverside University Health Sciences Center Trauma Center, St. Bernardine Medical Center, Community Hospital of San Bernardino, San Antonio Regional Hospital, Kaiser Permanente Fontana Medical Center, and Kaiser Permanente Ontario Medical Center.

After the resolution of the active incident (Days 2 - 7), local and federal law enforcement investigations were supported by the Fire Department's Type 3 IMT. LE personnel unfamiliar with ICS and IMT concepts initially resisted cooperation. However, within a few days, they had become more comfortable and accepted the benefits that the IMT provided through logistic support for their multi-day forensic investigation.

AAR FINDINGS

Overall, the operations observed in this incident can be characterized as efficient and appropriate. Thirty-one minutes after the first call to 911 reporting the shooting, SBPD and probation officer action brought the first patients to Triage A. Within 18 minutes of arrival of the first patient, SBFD triaged, treated, and transferred 14 patients for transportation to the hospital. One hour after the first 911 call, the last immediate patient was in an ambulance on the way to the hospital.

SBFD assisted in the movement of twenty patients from the IRC to area hospitals. Probation officers drove one patient from the CCP, Sheriff's Air Rescue transported two patients, AMR transported twelve patients from Triage A and five patients from Triage B. Three patients went by private vehicle to area hospitals.

In total, 28 people went to the hospital and 14 died at the scene from non-survivable gunshot wounds. All patients extracted from the IRC survived.

In order to better understand the accomplishments and gaps identified in this After-Action Report (AAR), it is important to first acknowledge the environment in which this event took place, as well as the environment's effect on operations. This event occurred in an environment best characterized as VUCA-T, modified from the US Army's characterization of the post-Cold War environment: Volatile, Uncertain, Complex, and Ambiguous. It was also an environment which contained a threat, hence VUCA-T.

WHAT WORKED AND WHY

The most likely reason for the efficient operations observed in this incident, despite the complex and challenging environment, was the use of several concepts not routinely discussed for these emergencies or in public safety.

- The rapid integration and cooperation of various public safety entities made for an almost seamless operation. Notably involved were the San Bernardino Police Department, San Bernardino City Unified School District Police Department, San Bernardino County Probation Department, San Bernardino County Sheriff's Office (and Air Rescue Service), and American Medical Response.
- Early response of a Type 3 Incident Management Team with the San Bernardino County Fire Protection District logistically supported the forensic investigation.
- Leadership of the San Bernardino City Fire Department reflected the traits identified from the military as "leadership in dangerous contexts" or "leadership *in extremis*." Members of the San Bernardino City Fire Department, at all levels, appeared to have high levels of trust and recognition that leaders would stand by them no matter the situation or outcome.
- The "leader-leader" form of leadership contributed to the initiative and improvisation demonstrated throughout the event. "Visual communication" played a prominent role in these operations. Various small unit actions self-organized to merge with each other and rapidly improvise solutions on the spot, a type of "self-organizing improvisation."
- Members of the San Bernardino City Fire Department repeatedly demonstrated elements of High Reliability Organizing (HRO). These are organizations with identified characteristics enabling entry into hazardous environments, such as those characterized by VUCA-T, with fewer than expected mishaps.
- The culture of the San Bernardino City Fire Department, having a form of high reliability culture, was embedded with a common social knowledge of how to operate in these situations. Members had the attitudes to engage, the duty to serve those in danger, and the freedom to act and improvise.

- Despite the threat exposure, San Bernardino City Fire Department personnel thought clearly and deliberately. They used a pragmatic, adaptive approach to solve problems effectively.
- Members of the San Bernardino City Fire Department and the San Bernardino County Fire Protection District professionally addressed the friction which occurred between FD/LE during institution of the Incident Command System (ICS) and the Type 3 Incident Management Team (IMT), owing to the unfamiliarity of law enforcement agencies with those operations.
- The public benefited from previous school active shooter training with the San Bernardino Police Department, the San Bernardino City Unified School District Police Department, other agencies, and local hospitals. The use of a problem-solving approach led to real-time, practical solutions that facilitated rapid movement of victims from the scene in the warm zone to area hospitals.

GAP ANALYSIS

The Strategic Reliability Team recommends that the San Bernardino County Fire Protection District institutionalize the aforementioned observed characteristics. However, the following gaps should also be addressed.

Address law enforcement interoperability

- Identify improved methods of force protection using both fire department and law enforcement assets.
- Identify improved methods for fire apparatus to access a large and intense law enforcement response.
- Identify improved methods for fire personnel to identify plainclothes law enforcement officers.
- Provide ICS and IMT services to law enforcement for prolonged activities, thus enabling law enforcement to focus on criminal activity and forensic investigations.

Evaluate EMS operations in the extreme VUCA-T environment

- Work with EMS agencies, using the experience of this incident, to improve the inefficiencies of EMS care.
- Evaluate prehospital care, field triage, and determination of death in a hazardous area and during a multi-casualty event. This should include identification and treatment of shock and pneumothorax.
- Work with the EMS agency and local hospitals to use this experience for improved coordination within the system.
- Evaluate operational parameters for an active shooter incident and any incident within a hazardous environment.
- Identify methods to support paramedic judgment in the VUCA-T environment.

Evaluate fire rescue operations in the extreme VUCA-T environment

- Investigate the effectiveness and utility of various Rescue Task Force configurations in diverse response scenarios.
- Investigate training for specific fire personnel to enter environments more hazardous than routinely encountered.

Communications

- Investigate passive radio communication to aid on-scene personnel following other agencies.
- Recognize the utility of visual communication in a noisy, distracting environment.
- Identify methods for cross-agency training such as with law enforcement and EMS.
- Improve training for the interaction with news media for personnel who have been approved for media interviews.
  - To connect with the public during these incidents.
  - To give the rationale and concerns of the fire department and, by describing fire department activities, to give reassurance to the public during these incidents.

As a result of this review, the Strategic Reliability Team provides detailed recommendations for institutionalizing the positive characteristics observed and addressing the gaps observed within the body of this report.

ABBREVIATIONS, DEFINITIONS, AND FIGURES

ABBREVIATIONS

AMR	American Medical Response Medical Transportation
AR	Air Rescue (helicopter)
AS	Active Shooter
BC	Battalion Chief (FD)
CAD	Computer-Aided Dispatch
CCP	Casualty Collection Point
CISD	Critical Incident Stress Debriefing
CISM	Critical Incident Stress Management
CP	Command Post
EMS	Emergency Medical Services
FD	Fire department or fire service
GSW	Gunshot Wound
IC	Incident Commander (LE-IC, FD-IC)
ICEMA	Inland Counties Emergency Medical Agency
ICS	Incident Command System
IED	Improvised Explosive Device
IMT	Incident Management Team
IRC	Inland Regional Center
LE	Law Enforcement
LEO	Law Enforcement Officer
PCR	Patient Care Record
PO	Probation Officer
RTF	Rescue Task Force
SBCoFD	San Bernardino County Fire Protection District
SBCPO	San Bernardino County Probation Department
SBCSO	San Bernardino County Sheriff’s Office
SBCUSD-PD	San Bernardino City Unified School District Police Department
SBFD	San Bernardino City Fire Department
SBPD	San Bernardino City Police Department
SWAT	Special Weapons and Tactics
UC	Unified Command

DEFINITIONS

Incident Action Plan: An oral or written plan containing generalized objectives reflecting the overall strategy for managing an incident. It may include the identification of operational resources and assignments. It may also include attachments that provide direction and important information for management of the incident during one or more operational periods. (FEMA)

Incident Commander: The individual responsible for all incident activities, including the development of strategies and tactics and the ordering and the release of resources. The IC has overall authority and responsibility for conducting incident operations and is responsible for the management of all incident operations at the incident site. (FEMA)

Incident Command System: A standardized on-scene emergency management construct specifically designed to provide for the adoption of an integrated organizational structure that reflects the complexity and demands of single or multiple incidents, without being hindered by jurisdictional boundaries. ICS is the combination of facilities, equipment, personnel, procedures, and communications operating within a common organizational structure, designed to aid in the management of resources during incidents. It is used for all kinds of emergencies and is applicable to small as well as large and complex incidents. ICS is used by various jurisdictions and functional agencies, both public and private, to organize field-level incident management operations. (FEMA)

Incident Management Team: The Incident Commander and appropriate Command and General Staff personnel assigned to an incident. (FEMA)

Public Information Officer (PIO): A member of the Command Staff responsible for interfacing with the public and media or with other agencies with incident-related information requirements. (FEMA)

Rescue Task Force: The Rescue Task Force is an aggressive response to an active shooter incident that provides rapid medical treatment at the point of injury to those who have been injured. The RTF is a joint response between Police and Fire/EMS departments but can be adapted to any agency large or small, rural or urban. The Rescue Task Force concept has been endorsed by the IAFF, NFPA, and FEMA. (ARK Medical)

Shrapnel: Fragments or splinters thrown out from a bullet or explosive device. Primary fragmentation is from a bullet, exploding bomb or shell, or objects intentionally embedded into an explosive device. Secondary fragmentation creates splinters or debris from the bullet or explosion (Browner et al. 2014,339).

Staging: Location established where resources can be placed while awaiting a tactical assignment. This area is typically outside the immediate incident perimeter, but close enough for quick response to the scene.

Triage & Treatment Sites: Designated Area in the warm / cold zone, for Fire Department and / or EMS assets to perform patient assessments & treatment.

Unified Command: An application of ICS used when there is more than one agency with incident jurisdiction or when incidents cross political jurisdictions. Agencies work together through the designated members of the Unified Command; it is often the senior person from agencies and/or disciplines participating in the Unified Command, to establish a common set of objectives and strategies and a single Incident Action Plan. (FEMA)

FIGURES

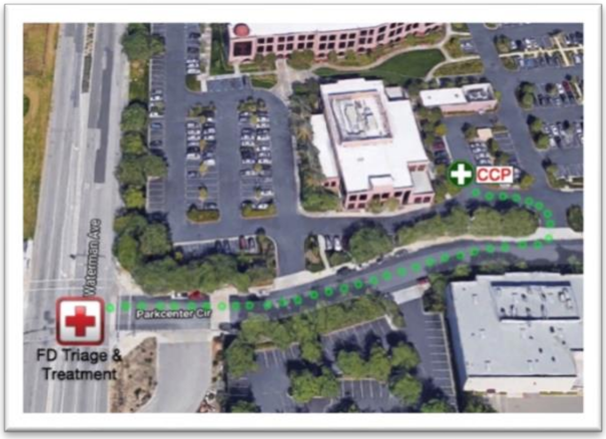

Figure 1: CCP, FD Triage / Treatment Area, and evacuation route from CCP. Map data: Google, Image Landsat

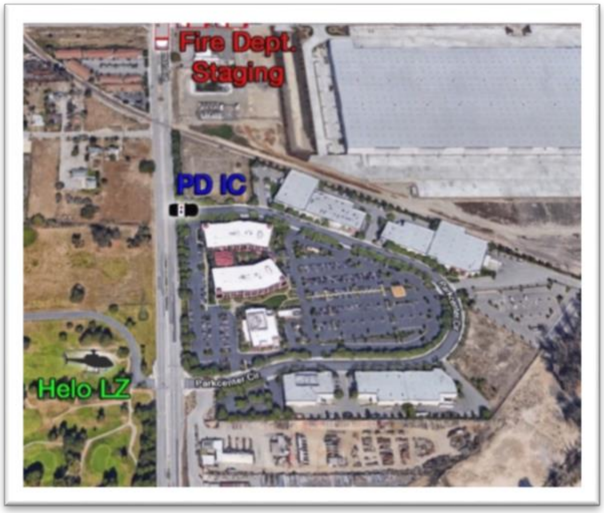

Figure 2: FD Staging, PD IC, and Helicopter LZ. Map data: Google, Image Landsat

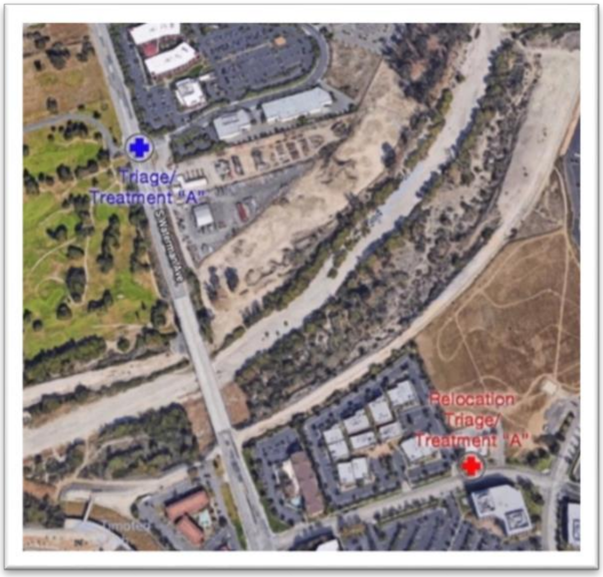

Figure 3: Initial Triage / Treatment area and secondary location. Map data: Google, Image Landsat

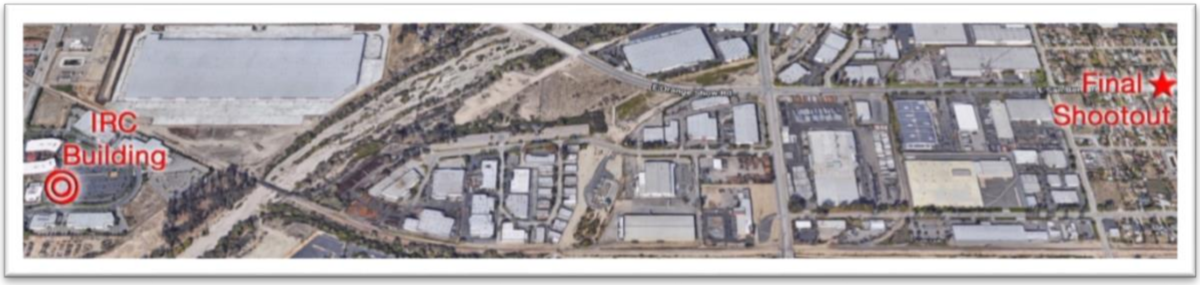

Figure 4: Location of final shootout in proximity to initial incident (IRC)

INTRODUCTION

"The success from that day came from cops getting in there and doing what they needed to do and from fire getting the victims to the hospital so quickly."

"There's nobody better than the Fire Department we had. I have nothing but praise for Fire from my needs."

The SWAT Commander and Police IC on December 2, 2015

On December 2, 2015, members of the San Bernardino County Department of Public Health were attending a training event. Decorations in preparation for an after-hours Christmas party misled some to believe this event was a Christmas Party. Two assailants entered the conference room and began shooting. During this attack, 14 people were killed and 22 more suffered penetrating trauma or injuries of varying severity.

The San Bernardino Police Department (SBPD) responded, joined by convergent law enforcement officers from the San Bernardino County Probation Department, San Bernardino City Unified School District, the San Bernardino County Sheriff's Office (SBCSO), and other regional police departments.

Every station of the San Bernardino City Fire Department (SBFD) responded to provide rescue and medical aid. Other fire departments provided mutual aid: Colton FD, Loma Linda FD, Redland FDs, San Bernardino County Fire Protection District (SBCoFD), and San Manuel FD.

American Medical Response Medical Transportation (AMR) responded to assist with medical aid and transport of patients to local hospitals. Symons Ambulance Service, with a physician-led team of nurses and paramedics, responded to staging in support of patient transportation to area hospitals.

Law Enforcement (LE) and Probation Officers (PO) moved 15 patients from the conference room to a hastily identified Casualty Collection Point (CCP) during the search for the assailants. This movement began 13 minutes after the first reported shots.

In 18 minutes (11:29 to 11:47 a.m.) SBFD transferred 14 patients from Triage A to AMR and the Sheriff's Air Rescue helicopter for transportation to hospitals. Probation officers drove one patient, triaged as "Immediate", directly to the emergency department (ED). No transported patient died.

Several lines of action, arising independently from diverse groups, converged to move casualties toward safety and medical treatment.

These lines developed from SBPD, Probation Officers (PO), San Bernardino City Unified School Police Department (SBCUSD-PD), SBFD, and AMR. The converging lines began in overlapping sequence yet arrived to aid casualties within a few minutes of each other.

1. LE made entry searching for assailants, the Special Weapons and Tactics (SWAT) team arrived, SWAT commander became Incident Commander (IC), SWAT medic began treating casualties, Sheriff's Air Rescue (AR) helicopter landed and the team moved to the conference room.

2. PO arrived, gave first aid to casualties in parking lot, entered conference room to give aid, set up Casualty Collection Point (CCP), extracted casualties, obtained vehicles for transportation, drove casualties to Triage Site A, then provided force protection at Triage A.
3. SBCUSD-PD arrived, joined the IC and consulted in the selection of a site for triage, then assigned school police officers to create a secure perimeter at Triage A.
4. AMR arrived, an LE officer directed the first ambulance to CCP, ambulances arrived at staging, SBFD identified routes for ingress and egress, an AMR supervisor arrived through the ingress route and confirmed ambulances could use the routes, then assigned an ambulance staging site.
5. SBFD arrived at staging, moved to triage, treated patients arriving moments later, SBFD team moved to the conference room, and SBFD began the process of transporting patients.

Each intersection of these converging lines of action created a new action.

1. The SWAT commander met with SBCUSD-PD and the triage site was identified and force protection created just before arrival of SBFD.
2. Convergent LE and PO met the SWAT Medic while initiating casualty treatment and extraction giving the SWAT Medic time for the primary triage.
3. PO began driving casualties from the CCP to Triage A. The Air Rescue medics met the SWAT Medic to enhance patient treatment and began a secondary triage.
4. SBFD completed set up of Triage A as casualties arrived. The SWAT Medic met SBFD at Triage A, accompanied them to the conference room and sent the ambulance crew to safety, then completed the secondary triage.

To accomplish these actions, public safety personnel worked outside of their training and normal practice.

1. LE began casualty treatment and extraction as SWAT started to clear the floor above and before the IRC building was fully secure.
2. LE officers and probation officers administered emergency first aid and moved urgent casualties to the CCP.
3. SBFD entered the conference room within the warm zone in response to information that there were potentially more victims. In the warm zone, they completed secondary triage in the conference room (an IED was later found in the room).
4. Probation officers treated casualties and moved critically wounded casualties to the CCP, placed them in vehicles, then drove the casualties to Triage A.
5. The ambulance entered the warm zone to assist casualties. The assailants were later known to have driven by the area twice (data downloaded from vehicle GPS) and the cold zone unknowingly became a warm zone.
6. Before SBFD IC (Fire IC) and LE IC could construct a Unified Command (UC), the SBPD and the SBFD commanders began fulfilling all of its functions.

There were no straight lines, people filled in the gaps they were not trained for, and everyone worked toward the same goal.

As resolution of the active incident occurred, SBFD and the San Bernardino County Fire Protection District recognized that the prolonged LE investigation would benefit from logistic support. Continuing their use of ICS, they brought in SBCoFD's Type 3 fire Incident Management Team (IMT) to the site. LE, not accustomed to implementation and operation of ICS or an IMT, initially resisted but then experienced the benefits of these assets.

PURPOSE AND SCOPE OF THIS REPORT

The charge to the investigative team in developing this After-Action Report (AAR) was as follows: First, to perform a gap analysis between guidelines, training and the demands of this specific incident. Second, to offer recommendations for improvement and to identify lessons learned.

This After-Action Report is specific to the fire service functions of fire, rescue, and EMS. It will, however, examine overlapping functions for an incident where circumstances brought the missions of law enforcement and the fire department personnel together. This AAR also describes the actions of mutual aid agencies that supported rescue, EMS, ICS, and IMT.

Note that there are two periods of time covered in this report, 1) the initial response for rescue and EMS during the active shooter incident and 2) long-term IMT support for the extended law enforcement investigation.

This AAR describes the actions of participants, identifies the science supporting such actions, and identifies concepts that can improve the public safety response to an active shooter incident. This is a reflective report intended for use as a tool to identify what worked, what can be learned, and what gaps exist in current operational and training models. This AAR does not examine the decisions or actions of the first responders through the benefit of hindsight, but rather places these actions within the context and complexity of the incident.

The San Bernardino County Fire Protection District commissioned this review on behalf of the San Bernardino City Fire Department. San Bernardino City Fire Department has since been annexed into the San Bernardino County Fire Protection District. The delay in developing this AAR of the San Bernardino City Fire Department's actions and decision-making resulted from administrative demands of incorporating a large fire department with its public services into another larger fire department.

FACTUAL NARRATIVE

THE SHOOTING AND LAW ENFORCEMENT (LE) RESPONSE

On December 2, 2015, the San Bernardino County Environmental Health Department conducted a training program in a conference room of the Inland Regional Center (IRC). Minutes before 11 o'clock in the morning two assailants dressed in black, later identified as Syed Rizwan Farook (the male assailant) and his wife, Tashfeen Malik (the female assailant), approached the conference room with semiautomatic rifles. Outside the exterior doors, they shot five individuals, killing three of them. They entered the conference room and fired upon attendees, shooting 36 attendees and killing 14 of them.

At 10:58 a.m., the San Bernardino City Fire Department (SBFD) dispatch received a call for a water flow alarm at the IRC building. Almost immediately the San Bernardino Police Department dispatcher, sitting at an adjacent desk, received a call for the same address reporting a shooting, "5 rounds heard–nothing seen." Fire and police dispatchers received phone calls for five minutes describing shooting inside the conference room.

Within two minutes of the first call, law enforcement (LE) officers were dispatched, arriving on scene six minutes after the first report of the shooting. Witnesses, however, had observed the assailants leaving the premises moments before arrival of the first law enforcement officer. Police officers from the San Bernardino Police Department, convergent police officers, San Bernardino County Probation Department (PO), San Bernardino City Unified School District (SBCUSD), and the San Bernardino County Sheriff's Office (SBCSO) arrived on scene.

A shooting victim with an injured arm had escaped and reached Building 2 where a pediatrician was evaluating clients for the IRC. The victim was pale and feeling faint but did say there had been a shooting in the conference room and they needed a doctor. The pediatrician used the victim's sweater to improvise a sling for her arm then went to Building 3 where his associates were working on the second floor. The security guard had ordered people to lock their doors and not leave. The pediatrician ran over to Building 3 to see if his associates on the second floor needed help. Because the shooters were still thought to be in the room, he used the stairway next to the entry door of the conference room. There was no answer at the door and police had not arrived. He then realized that, because he was Middle Eastern descent, he might be confused with any assailants. He ran back to Building 2 where he gave aid to those suffering severe emotional trauma during the incident and continued assistance when his group was evacuated to the golf course.

An SBPD sergeant arrived and created a command post (CP) on the trunk of his car parked on Waterman Ave. just south of Parkcenter Circle North, in front of the IRC. SBCUSD police arrived at the command post.

The first LE responders on scene took immediate action to enter the building and mitigate the threat. Within one minute of each other, two law enforcement teams of four officers each made entry into the building. Some of the first LE officers to make entry were convergent LE officers from other cities. PO immediately encountered wounded victims in the parking lot who were asking for assistance and medical aid.

The San Bernardino Police Department Special Weapons and Tactics (SWAT) team, with 12 team members and a SWAT Medic (a San Bernardino City Fire Department firefighter/paramedic and armed reserve police officer with collateral duties attached to the SWAT team) responded from a training session. As SWAT arrived, people were running from the building past the team.

The SWAT Commander observed LE vehicles were arriving from the south, congesting the north bound lanes. He then notified SBPD dispatch to have arriving LE units come from the north, for SBFD to stage to the north at Waterman Ave. and San Bernardino Ave, (Orange Show Rd.), and for all arriving LE units to change to the SBPD frequency. Arriving LE officers did not receive the frequency change notification.

The SWAT Commander reached the doors of the conference room then withdrew from the "hot zone" to become IC at the CP. He went to the CP the SBPD sergeant had established on the trunk of the SBPD vehicle.

The rapid influx of convergent law enforcement officers congregated loosely in the area of the CP, some moving to the IRC building. The IC assigned supervisors to form squads of 12 officers for various assignments with one squad to Triage A for force protection in conjunction with SBCUSD police officers.

RESCUE AND EXTRACTION

The SWAT Commander and SBCUSD police discussed placement of the casualty collection point (CCP) and triage site (Triage A). They selected the parking lot in front of the conference room for CCP and the golf course area for Triage A, located one hundred yards from the hot zone. The intersection of Waterman Ave. and Parkcenter Circle South has an unpaved continuation of Parkcenter Circle into the golf course. School police command also assigned school police officers to Triage A for force protection.

The LE IC directed additional incoming police to extract all living casualties and to acquire vehicles of opportunity (VOO). After SWAT officers moved past the conference room, but before the first floor was completely cleared, the assigned officers entered the conference room to extract casualties. These initial extractions from the conference room were technically performed in a hot zone by law enforcement.

Once these VOOs were obtained, the law enforcement officers loaded the casualties at the CCP and drove them to Triage A.

The SWAT Medic, upon his initial approach to the southeast corner of the IRC building, observed multiple victims outside, two of which were breathing with audible obstruction. He quickly performed a positional airway, improving their respirations, then followed his partner into the building to assist in clearing the first floor. It took the SWAT medic and his partner approximately one and a half minutes to finish clearing their designated area on the first floor. They then linked up with the other entry personnel at the stairs. The first floor was determined to be clear. The SWAT medic notified his team sergeant he intended to transition to patient care and broke off from the team, returning to the conference room.

LE officers, minutes after the SWAT Team entered the building, began moving victims out to a hasty CCP just outside of the southeast entry point. Convergent LE officers, many of them probation officers, assisted police officers in physically moving the casualties to the hasty CCP the IC selected in the parking lot just outside the conference room doors. The SWAT medic quickly assisted the PD officers with improvised victim movement of those still alive (e.g., the use of chairs), then began triaging those victims still in the conference room. He moved counter clock-wise, systematically assessing each victim.

After two deceased victims had been hastily moved by law enforcement to the CCP and then to the triage/treatment area, the SWAT medic used medical tape to mark the deceased by wrapping tape around the victim's wrist. This clearly differentiated between deceased and living to guide law enforcement officers assisting with the wounded.

Law enforcement officers also obtained vehicles, driving the casualties to Triage A site. LE had delivered these two deceased victims to Triage A just after San Bernardino City Fire Department units set up the site, eleven minutes after the start of victim extraction.

As the SWAT medic was completing primary triage and death determination, he heard reports of an additional 20 victims. About that time, a Sheriff's Air Rescue flight medic entered the conference room to assist. The flight medic assisted with removal of casualties and confirmed death in the deceased.

Observing SBFD personnel at Triage A, the SWAT Medic approached them for assistance with managing the potential additional casualties in the warm zone. An ad hoc team accompanied the SWAT medic to the conference room and conducted systematic secondary triage and confirmation of death. They also placed triage tags on casualties and the deceased. They then returned to Triage A.

Fire officers advised an AMR ambulance, on scene at the CCP, to move to Triage A for safety. A LE officer had earlier directed this early arriving ambulance to the CCP.

FIRE RESPONSE, TRIAGE AND TREATMENT

The initial SBFD response was four companies (three engine companies and one truck company), three ambulances, and one battalion chief. Two companies, hearing the initial assignment, volunteered to respond. A second call brought an additional nine units to the scene. Staging was initially the LE CP but quickly changed to a site north, Waterman Ave. and Orange Show Rd.

All responding SBFD personnel interviewed made specific reference that their fire units had a SBPD portable radio assigned for the purpose of monitoring calls. Many of the first responding units stated that this capability greatly enhanced their fidelity of the scene prior to their arrival and while staging. On average, the SBFD personnel stated they would hear critical on scene information approximately two minutes prior to the information being relayed over the SBFD dispatch.

While in triage, the fire battalion chief (BC) assigned predesignated duties for medical communication, triage, treatment, transportation, and staging. As fire department IC (FD IC) he drove toward the CP to form a unified command (UC) with the LE IC. Parked police vehicles blocked his access to the CP.

The medic engine company assigned medical communication (MedComm) notified the EMS system (ReddiNet) at 11:12 a.m. of an active shooter incident with possibility of 20 victims. A second medic engine company notified the Loma Linda University Medical Center of the incident.

ReddiNet sent an advisory of the incident at 11:17 a.m., "MCI: Actual/San Bernardino/**Mass Shooting** This is not a drill/ Waterman X Park Center San Bernardino/ Est Pts 20 Ini." ReddiNet then initiated hospital polling for bed availability, transmitting the total to MedComm at 11:24 a.m.

An LE officer at Triage A waved the fire companies in, but without radio confirmation, they were held in staging. Radio incompatibility between the San Bernardino City Fire Department and the San Bernardino Police Department prevented the LE IC from communicating the location of Triage A to fire/EMS personnel. The IC broadcast for any

police officer to communicate the location and for fire/EMS to move from staging to set up the triage/treatment site.

The fire Incident Commander observing the police officer at Triage A and not receiving countermanding orders from the police Incident Commander, made the decision to move fire units to the triage sites. The units moved in either by driving or walking depending on road congestion and began setting up for their mass casualty incident (MCI) protocols.

The number of LE vehicles on the road made access to Triage A difficult. Some fire companies hand carried life-saving equipment several hundred yards. One company, risking entrapment of their vehicle, drove the same distance against oncoming traffic.

Triage A was set up with salvage covers because the large MCI kits with colored triage tarps had been removed earlier for other purposes. Salvage covers were placed in the ground with chalk marking for “Immediate,” “Delayed”,” Minor”, and “Deceased.” A fire engine blocked the view of the deceased tarp from the area where evacuees would be placed.

While Triage A was being set up an LE officer arrived to advise that they were bringing patients out soon. Almost immediately, patients arrived on foot or in VOOs. Patients were triaged from the VOOs and placed on the tarp matching their triage level. One deceased patient was placed first on the immediate tarp then moved to the deceased tarp.

Triage level changed with treatment or time. Needle decompression of the chest shifted an immediate patient to delayed. Complaints of back pain and a repeat examination revealed two gunshot wounds to the back, changing the level of triage from "Delayed" to "Immediate.” In this patient, bullet holes were not visible in the shirt and there was no blood at the site of the entry wound.

TRIAGE

All medics used visual triage of 1) activity, 2) skin, 3) breathing, 4) blood, and 5) anatomic site. Blood pressures and numerical respiratory rates were not obtained. Carotid pulse was palpated for absence or presence. Triage scores and triage tags were not used for decisions to treat or transport.

1) ***Activity.*** Those triaged as more serious had the “look of death”, an anxiety distinct from anxiety due to the incident. All patients were quiet with little talking. In those cases, the medics reported evaluating respirations as the possible cause of the silence (below).
2) ***Skin***. Pale skin had higher triage priority.
3) ***Breathing.*** Gasping breathing was most significant followed by evaluation for rapid breathing. Two patients with gasping breathing responded to needle decompression and the medics moved the patients from "Immediate" to "Delayed".
4) ***Blood.*** The site with visible blood was evaluated for bandaging or tourniquet. All medics stated they evaluated for a tourniquet in order to move a patient from “Immediate” to “Delayed”. Wounds to chest, abdomen, and back had little external bleeding.

5) ***Anatomic site.*** After activity and breathing, anatomic site had greatest influence in triage for treatment and transportation. Head, chest, and abdomen wounds were classified as “Immediate”. Extremities were classified as delayed. The shoulder, considered by medics as an extremity, was evaluated for any sign of chest penetration, which would give the patient an “Immediate” level triage.

Intravenous access, mostly acquired en route, was obtained in 12 of the 18 patients transported with gunshot wounds. Four patients received a fluid bolus of 300-500 mL normal saline. In one patient, the heart rate decreased from 120 beats per minute to 110 beats per minute and in the other three, there was no change in physical examination or vital signs.

Some patients, on arrival to Triage A, had become emotional. Medics took additional, unrecorded and unreported, measures to calm patients.

DEATH DETERMINATION

Determination of death occurred in the warm zone, conference room, and the cool zone, Triage A. These patients were ashen gray, not breathing, and not moving. Medics who determined death in the conference room identified obvious injuries inconsistent with life (those missing a portion of cranium or brain matter) or massive wounds. The medic felt for presence of carotid pulse, if absent he checked the other carotid artery. Then the medic made a death determination. At least two medics made the determination using separate examinations.

TRANSPORTATION

A single northbound lane on Waterman was sufficient for ambulance passage to Triage A. Ambulance staging was placed at Waterman and Hospitality Lane to the south.

All paramedics spontaneously placed the more seriously injured patients toward the “ambulance side" of the “immediate” tarp. Wounds to head or trunk went to one of three trauma centers and extremity wounds went to medical centers.

Several ambulances arrived before the patients did, which allowed immediate patient movement toward hospitals. During a pause, between sets of ambulances, the Sheriff’s air rescue transported two patients to Riverside University Health System Medical Center. The medical center did not receive notification of this transport.

TRIAGE B

Triage B was set up near an abandoned house on Waterman near Parkcenter Circle North. Approximately 100 individuals were gathered there. AMR transported five patients to the hospital.

MOVE SITE

Law enforcement requested the triage area move from Triage A to another site due to the discovery of a suspicious device and concerns that a third assailant might have remained in the area. The San Bernardino City Fire Department personnel moved the triage/treatment site to FS 231 due to additional security concerns.

Symons Ambulance arrived at Waterman and Hospitality Lane with a physician-led team of nurses and paramedics, then moved to FS 231, for staging at 12:38 p.m.

GOLF COURSE

Several hundred people had evacuated to the golf course. A few began developing medical complaints. MT 224 with the SBFD EMS Nurse, evaluated patients and identified one who was driven by an all-terrain vehicle to FS 231, the site where Triage A had moved.

EMS SUPPORT

ICEMA (Inland Counties Emergency Management Agency) initiated operations to coordinate hospitals and ambulances. Medical Health Operational Area Coordination (MHOAC) is the regional coordinator for the state of California and prepared ambulance strike teams of five ambulances per team to respond from out of the county.

OTHER RESCUE UNITS

Other teams were created during the course of this incident. A rescue task force (RTF) responded for mutual aid from the Rancho Cucamonga Fire Department and another ad hoc RTF was created from mutual aid companies.

RESCUE TASK FORCE

ST 6210A was split into two at 3:23 p. m. to form an ad hoc RTF to support LE. It was created from ME 174A, ME 134A, and ME 65A with sheriff’s deputies, then assigned to their headquarters on Rialto Ave.

STRIKE TEAMS

- ST 6210A with BC 604 staged San Bernardino Ave. and Tippecanoe Ave.
- One strike team was formed from ME 174A, B 2112, and AMR to standby for a search warrant.
- A strike team with a BC and three engines and three ambulances was formed for any response in the area related to the incident.

JOINT TERRORISM TASK FORCE, RANCHO CUCAMONGA FIRE DEPARTMENT

The fire department liaison for the FBI JTTF in the Inland Empire (from Rancho Cucamonga Fire Department, RCFD) was requested to report to the command post at the FBI headquarters in the City of Riverside. He responded code 3 with the RCFD Training Captain (T 2120) to the Command Post at the incident. After arriving on scene, the RCFD TLO made face-to-face contact with the TLO from SBPD who advised him of critical confidential intelligence pertinent to the scene and its potential evolution. The Rancho TLO and 2120 integrated with multiple duties at the command post, and two additional Chiefs from Rancho Cucamonga Fire Department responded to the Command Post. During this period of time, there were still incoming tips and additional threats being called in to area hospitals, freeway off-ramps, and area businesses.

CHIEFS ON SCENE

- The fire chief responded and checked in with SBPD IC, SBFD IC, Triage A, and FBI. He held a command meeting.
- SBFD IC arrived on scene but physically could not reach LE IC for a unified command. The SBCUSD PD Emergency Manager had been on scene shortly after the first LE dispatch. He met FD IC and used the SBCUSD PD radio capacity for communication between the two ICs. Later he physically connected the two ICs.
- The second arriving SBFD BC arrived and assumed FD IC, allowing the first FD IC to assume tactical operations command. Other Battalion Chiefs arrived and assumed duties to support fire companies and fire operations.

FORCE PROTECTION

SBCUSD police officers and County probation officers provided force protection at Triage A. SBPD police officers provided force protection to Triage B and FS 231 (moved Triage A). Sheriff’s deputies, meanwhile, were assigned to provide force protection for the RTF from the Rancho Cucamonga Fire Department as well as the ad hoc RTF.

During the shootout with the suspects, force protection officers from the ad hoc RTF and the secondary triage site responded to aid police officers involved. During this period, the two RTFs and secondary triage site had no force protection. SBCUSD police remained at the original Triage A site.

SECONDARY INCIDENTS

SBFD, on behalf of SBPD, notified the Burlington, Northern, and Santa Fe Railroad to close the railway near Orange Show Road at 2:00 p.m.

Law enforcement activity requiring San Bernardino City Fire Department support continued throughout the day. Secondary incidents related to the terrorist event and responded to by San Bernardino City Fire Department personnel for staging include:

1. A shootout between the suspects and law enforcement officers approximately 4 hours after the initial attack. ME 222 self-dispatched, then a BC directed them over the radio to meet and assist AMR, which they did. An RTF was sent with sheriff to stand by for this incident, 3:45 p.m.
2. A reported shooting at Patton State Hospital, 12:53 p. m. ST 6210A (per fire chief).
3. A reported shooting at the Amazon Fulfillment Center (per fire chief)
4. Staging for a search warrant regarding a possible third suspect (per fire captain).

By 12:30 p.m. the San Bernardino City Fire Department requested fire department chaplains from numerous fire departments. Some responded to SBSO headquarters and others to the Rock Church where evacuees were located.

After the patients had been transported from the scene, fire units and law enforcement officers formed an extemporaneous rescue task force with fire units and law enforcement standing by should they be needed. During the shootout, the law enforcement officers with this task force self-deployed in support of law enforcement at the shooting, leaving their fire counterparts behind. A fire rescue task force from the Rancho Cucamonga Fire Department had also responded to the second scene.

That evening, the Redlands Fire Department deployed in support of Explosive Ordinance Disposal technicians from several counties when SWAT discovered equipment and supplies to construct improvised bombs at the residence of the assailants.

METHODOLOGY

THE AAR TEAM

Upon assignment of the After-Action Report from the San Bernardino County Fire Protection District (SBCoFD), Strategic Reliability, LLC, gathered a team of experts experienced with operations in dangerous contexts, the academic evaluation of injuries, or the academic evaluation of catastrophic events. San Bernardino City Fire Department (SBFD) had initiated an AAR within a month of the incident. The purpose of this AAR was to complete the AAR with the addition of the psychological element and High Reliability Organizing.

Selected members came from the disciplines of operational military, fire service, law enforcement, EMS, nursing, psychology, neuropsychology, critical care medicine, trauma surgery, and human factors engineering. Most of the experts had experience in multiple disciplines, giving a broad perspective of emergency response operations. Knowledge of High Reliability Organizing (HRO) and experience with operations in or the study of dangerous contexts was a major determinant for selection.

METHODS

The principle information for analysis came from interviews of volunteers. These interviews were predominantly conducted by two interviewers, with at least one interviewer having operational experience in dangerous environments.

The AAR team toured the site escorted by fire officials to view the shooting location, conference room, casualty collection point (CCP), and triage/treatment site A (Triage A).

The AAR team used the principles and theory of HRO to structure the presentation, analysis, conclusions, and lessons learned for this AAR (See Appendix HRO). Time lines and descriptions of actions taken by individuals came from archived information and interviews. The medical and organizational science literature informed the analysis and conclusions section of this report.

MEDICAL INFORMATION

Medical information came from interviews, redacted paramedic written reports (Patient Contact Record or PCR), and redacted autopsy reports. A team of trauma surgeons reviewed autopsy reports, which they discussed with the AAR team.

TRIAGE

Triage occurred at three points: triage for treatment, triage for transport, and triage for destination. The type of triage scoring system or other method utilized were obtained through interviews.

FIELD TREATMENT

Redacted patient care records from AMR provided treatment information.

MATERIALS

PREVIOUSLY COLLECTED AND ARCHIVED DATA

In July 2017, the San Bernardino County Fire Protection District released to the Strategic Reliability team preliminary material developed internally by the AAR team for SBFD. This included interviews, dispatch times, site maps, photos, published material in EMS professional journals, and an analysis of injuries. The material had been sequestered while the San Bernardino County Fire Protection District incorporated the San Bernardino City Fire Department.

The Coroner's Office made available redacted autopsy reports of the deceased. AMR made available redacted patient contact records related to the event including dispatch and transport times.

SBFD and the San Bernardino City Unified School District (SBCUSD) Police Department provided archived computer-aided dispatch (CAD) data. Law enforcement dispatch times came from the San Bernardino Police Department (SBPD) AAR (Braziel et al. 2016) along with other information regarding the police response.

SBFD, SBPD, SBCUSD-PD, and AMR made available participants from the incident who volunteered for interviews.

INTERVIEWS AND NARRATIVES

Points of Contact (POC) were identified for the various entities engaged in the active shooter incident at the Inland Regional Center (IRC). The POCs were offered the opportunity to contact the AAR group with questions, concerns, or information.

An EMS nurse for SBCoFD, previously with SBFD, requested volunteers for interviews from SBFD personnel who participated in events related to the December 2, 2015 active shooter incident. The AAR team contacted AMR for volunteers. Interviewees signed a consent for the interviews, which were scheduled for 45 minutes and could be terminated by interviewees at their request. One or two members of the AAR team interviewed personnel by phone or in person at an SBCoFD facility. Interviews typically lasted 45 minutes to two hours. Some interviewees were contacted later for clarification and some contacted the team to learn more about the process.

A nurse from Riverside University Health System - Medical Center voluntarily submitted information relevant to the findings of this AAR.

Standard questions, listed below, were asked and the interviewees were free to talk about any topic related to the incident or the interviewer.

1. When you received the call or arrived ONS (on-scene), what did you think would happen? What did you expect to do?

2. What actually happened?
3. Can you explain why things went that way? What was the gap between what you thought and what actually happened?
4. Based on what you learned, if you had to do this again, what do you think you'd do next time?
5. What do you want others to know, now that you've had this experience?
6. Did you read AARs of past events? Did you use anything from those earlier AARs in this event?
7. If you were giving a course or lecture, what would you say?

Anonymous interviews were then conducted with the respondents in civilian clothing. Name, rank, and assignment were not asked of any member interviewed, although many volunteered each. Handwritten notes were transcribed and aggregated for use in this report, and then destroyed.

Some interviews were conducted within weeks of the event by SBFD personnel and some 18 months later by the AAR team. A few of the interviewees were interviewed at both time intervals. The team recognized the time delay could influence accuracy of memory but also understood the time interval would give interviewees time to identify the utility of what they learned.

LITERATURE REVIEW

Medicine has a justified preference for recent research (Ioannidis 2016), often with a preference for articles less than ten years old. For other research, there is strength in an established concept. Background research for this report is from numerous sciences including medicine, organizational behavior, physics, complexity science, social psychology, and psychology. Literature from the public safety disciplines included firefighting, law enforcement, and EMS.

DATA EVALUATION

NARRATIVES

One author (DvS) aggregated transcribed interviews for a sequential description of the incident and a second aggregation related to tasks such as staging, triage, treatment, communication, and command. A second author (SM) reviewed individual interviews and recorded dispatch information.

This produced a view of the incident from an extremely local level to a more comprehensive view of operations. It also provided descriptions, in the participants' own words, of their thinking, actions, objectives, goals, and how they identified and resolved obstructions. This also revealed communication at multiple levels, from the environment, visual, face- to-face, and radio.

INJURIES

An active shooter incident: causes injuries, can exacerbate existing diseases, and create acute illnesses. Evacuees may shelter in place or be moved to a safe harbor, either of which may take place over an extended time period, depriving them of routine or timed medications. We created pragmatic definitions for injuries, wounds, disease, and illness following the definitions of Harry Emerson (1987).

HEALTH, DISEASE, INJURY, OR ILLNESS

Disease has a demonstrated pathophysiology that can be investigated, such as hypertension, and is evaluated by objective measures (signs) and subjective measures (symptoms). Illness is how the person experiences a disease--a personal measure described by subjective measures (symptoms).

For this report we differentiate wounds, injuries, disease, and illness.

- Injury is body damage from external energy. Examples include abrasions or sprains from trips or falls during escape.
- Wound is an injury that breaks the skin.
- Gunshot wound (GSW) is a bullet wound with high kinetic energy.
- Shrapnel wound is a lower energy wound from bullet or bomb fragments or fragments thrown off by a bullet or explosion.
- Disease is a medical disease either being treated by a physician or surgeon ("existing disease") or incident related such as syncope, angina, or myocardial infarction.
- Illness is the personal experience of distress possibly associated with a disease but not always. Examples include anxiety and fear.

Injuries described by paramedics on the patient care record (PCR) were recorded from the redacted EMS PCRs. When available, hospital findings were compared with this information. Injury data included cause, anatomic site of injury, and treatment provided before and during transport.

DECEASED VICTIMS

For deceased victims, information was obtained by interviews with paramedics for method of determination of death and autopsy reports for cause of death. Autopsy findings from the Coroner's Report contained circumstances of death, external and internal findings, and cause(s) of death with estimated time to death.

Trauma surgeons and a trauma nurses reviewed records, the environment, and the circumstances of determination of death and death.

Survivability was identified from the interaction between time and severity of wounding. Final determination of survivability was from discussion between the authors and trauma surgeons.

TIME SEGMENTS: CLINICAL AND OPERATIONAL

Times were obtained from a computer-aided dispatch (CAD) system. CAD data came from SBFD, SBCUSD-PD, and the SBPD CAD data reported in the SBPD AAR *Bringing Calm to Chaos* (Braziel et al. 2016). Helicopter transport times came from redacted patient care records provided by San Bernardino Sheriff's Air Rescue. AMR provided ambulance times from redacted patient care records.

TIME LINES

A time line was constructed for the activities of the assailants, victims, LE, ambulance transport, and SBFD. During the analysis of the various time lines, there were discrepancies and ambiguity within the collected data. Much of this ambiguity is attributed to high radio traffic, units talking over each other and a limited number of dispatchers. These friction points were obvious when listening to the dispatch audio files and were repeatedly verbalized by responders during the interviews. Where ambiguity existed, events from the narrative were used to clarify the time of an event and the order events occurred. A best guess, when necessary, was made by the lead author (DvS).

WOUNDING TIME

Wounding time or time from wounding, is the measure of time as a comorbidity that exists when a public safety agency assumes care of the patient. It expresses the increasing magnitude of injury and decreasing survivability as a function of time. We measured wounding time as the time interval between the first 911 call and transfer to the next service. This approximates time of wounding which could have occurred before or after the first 911 call. Generally, this is the time of 911 call to time at triage for the fire department, time of first transport for the ambulance service, and time arrived at the emergency department for the hospital. For the EMS system, it is also time from 911 call to time of arrival at a hospital.

For LE, we use time to threat mitigation, similar to time from wounding, and *time to triage*. Time to threat mitigation is measured the same as time from wounding, 911 call to time LE has control over the casualty. An additional measure is a goal-oriented measure, *time to triage*. In this incident, LE also had a mission to move the casualty into mass casualty incident (MCI) care and the EMS system independent of threat mitigation.

Time to threat mitigation is the duration of time the armed suspect(s) has access to potential victims beginning from the first act of wounding to the moment the threat of the suspect(s) is mitigated. Mitigation is measured by cessation of the suspect's ability to create new wounds, e.g., the suspect is killed, suicide, is arrested (by police or citizen intervention), flees, barricades or takes hostages, etc. The longer the delay in successfully mitigating the suspect(s)'s threat, the higher the number of dead and dying there likely will be.

These times have meaning for the different services. For LE, it measures victim exposure to assailants; for the fire department it measures capacity to receive patients; for the ambulance service it may influence mode of transportation and destination; for hospitals it describes the added burden of time to the injury; and for the EMS agency it characterizes the effect of overall system operations and function on time as a comorbidity. This can include trauma center arrival versus non-trauma center arrival.

OPERATIONS TIME

Time of operations: measures the total time a casualty or patient is in the care of a specific public safety service. This is the operationally relevant time the service can influence.

Law enforcement operations: from time of entry into the building to time of arrival at the triage site. It is too confusing during operations to track the last immediate patient to arrive at triage. Casualty collection points (CCP) are not included.

Fire department operations: from arrival time of first patient at the triage site to the last immediate patient transported from triage. This is the period of triage, treatment, coordination of care, and staging operations.

Ambulance transport operation: from time of first patient transported to the last immediate patient arrival at the emergency department. This is a measure of the availability of ambulances, surge capacity, and knowledge of transport times for various healthcare facilities.

EMS regulatory agency operations: from time of law enforcement entry to time of arrival of the last immediate patient at an emergency department.

OTHER TIME MEASURES

***Time to first transport**:* total operational time from first LE on scene to time of first transport. This measures integration of LE, fire department, and ambulance transport.

Triage time**:** time measured from arrival of the first patient at the triage site to the last immediate patient departing to the hospital. This has both clinical and operational relevance for triage operations.

Survivability time: the time from wounding to the time of emergency department care. Survivability has an inverse relationship from time of wounding to fire/EMS custody, time of arrival at triage to transport, and time from arrival at triage to time of arrival at the hospital. The greater the length of time, the greater the decrement to patient survivability.

These time measurements can assist in analysis of clinical and operational areas to identify best practices, focus on areas for improvement, and articulate lessons learned.

DATA

INJURIES AND ILLNESS

TRIAGE AND TRANSPORTATION

Triage Level	Number
Immediate	12
Delayed	1
Minor	7
Missing Tag PO/Ambulance	(2)

Triage Site	Number
Triage A	15
Triage B	5
**Total**	**20**

Mode	Number	
Ambulance	17	
Helicopter	2	
LE Vehicle	1	
Private Vehicle	3 Total	
	Same Day (1)	Next Day (2)

TRIAGE TAGS

Not used for one patient transported by ambulance.

INJURIES, WOUNDS, ILLNESSES

Injury	Number
Gun Shot Wounds	15
Shrapnel	1
Abrasions	1
Graze Wounds	1
Orthopedic	1
Syncope	1
Total	20

Injury discrepancy: field to emergency department

Patients may have more than one discrepancy.

Observable injuries on scene under-represented the severity of injuries identified in the hospital. While we recommend investigating these discrepancies, in no case would they have changed a patient's triage level.

LEVEL OF TRIAGE

"Immediate" or "Delayed"

- Severe bleeding leg wound, discharged from emergency department with graze wound.
- Superficial facial injury, transported immediately, surgical repair of facial fracture.
- Back [chest] with graze wound; in emergency department found to have pneumothorax, received chest tube and endotracheal intubation.
- Abdomen with (appears to be) shrapnel; in emergency department patient transferred to operating room for significant abdominal injuries.
- Suspected hypovolemia, fluid bolus 300 mL normal saline, heart rate decreases 120 to 110 beats per minute; in emergency department received blood transfusion.

"Minor" level

- Minor, home without care, transported by private vehicle to emergency department, chest radiograph showed shrapnel in chest.

TREATMENT IN TRIAGE AND TRANSPORT

Therapy	Number
Intravenous catheter	12
Fluid bolus, normal saline	4
Needle decompression of chest	2
Oxygen therapy	5
Tourniquets	0

WOUNDING PATTERN

SUMMARY, ANATOMIC SITE OF NONFATAL VERSUS FATAL

Anatomic Site	Fatal	Nonfatal
Victims	14	15
Head, Neck	3	0
Face	2	0
Chest, upper back	25	8
Abdomen, pelvis, lower back	6	4
Upper extremity	5	7
Lower extremity	9	9
**Total**	**50**	**28**
Wounds per victim	3.57	1.87

SURVIVORS

TRIAGE LEVEL AND TREATMENTS

Patient Location	Triage Level	Treatment
CCP	Immediate 1	d/c from hospital
Triage A	Immediate 11<br>Delayed 1<br>Minor 2	IV 12<br>Bolus 4<br>O_2 5<br>Thoracostomy 2
Triage B	Minor 5	0
Police Officer<br>(Off site)		IV<br>O_2

MORTALITY PATTERN: CAUSES OF DEATH

Anatomic Site	**Cause**
**Head and neck**	3
**Chest**	10
**Abdomen**	6
**Femoral vessels (junctional)**	1
**Total**	20

*Some deceased had multiple fatal wounds.

*Femoral vessels- Includes junctional bleeding in groin or axilla.

TIME LINES

Overarching Time line			
10:58 a.m.	Wounding	11:29 a.m.	Initial sweep first building complete<br>First patient arrives Triage A
11:00 a.m.	LE officers dispatched		
11:06 a.m.	Room entry	11:33 a.m.	First patient transported by ambulance
11:11 a.m.	First floor cleared<br>Patients extracted to CCP	11:41 a.m.	First patient arrival at hospital
		11:47 a.m.	Last patient transported by ambulance

Service	Wounding Time			Operational Time		
	**Start**	**End**	**Time**	**Start**	**End**	**Time**
**Law Enforcement**	See table below					
**Suspect Mitigation**	10:58 a.m.	11:11 a.m.	13 minutes	11:06 a.m.	11:29 a.m.	23 minutes
**To Triage**	10:58 a.m.	11:29 a.m.	31 minutes			
**Start of care**	10:58 a.m.	11:11 a.m.	13 minutes			
**Fire Department**	10:58 a.m.	11:29 a.m.	31 minutes	11:29 a.m.	11:47 a.m.	18 minutes
**Ambulance**	10:58 a.m.	11:33 a.m.	35 minutes	11:33 a.m.	12:21 p.m.	47 minutes
**EMS system**	10:58 a.m.	11:41 a.m.	43 minutes	11:06 a.m.	12:21 p.m.	1 hour 15 minutes

Law Enforcement times		
**LE mitigates threat (LE Officer dispatched)**	11 minutes	11:00 a.m. – 11:11 a.m.
**Mitigation – EMS, triage**<br>**Mitigation – EMS, transport**	18 minutes<br>23-36 minutes	11:11 a.m. – 11:29 a.m.<br>11:11 a.m. – 11:34-47 a.m.

Casualty times		
**Wounding – room cleared**	13 minutes	10:58 a.m. – 11:11 a.m.
**Wounding – EMS, triage/treat**	31 minutes	10:58 a.m. – 11:29 a.m.
**Wounding – EMS, transport**	36-49 minutes	10:58 a.m. – 11:33-47 a.m.

Fire Department Times		
EMS triage- transport, first	4 minutes	11:29 a.m.- 11:33 a.m.
EMS triage- transport, last immediate	18 minutes	11:29 a.m.- 11:47 a.m.
First- last transport	14 minutes	11:33 a.m.- 11:47 a.m.

ANALYSIS: TIME LINE

MULTIPLE TIME LINES IN AN ACTIVE SHOOTER INCIDENT

Having separate timelines in an interactive situation with different teams and objectives can bring into view the perspectives of the different parties. In this incident, the four parties are the suspects, victims, law enforcement, and fire department. Essentially, the suspect active shooter wants to create as many wounds as possible before law enforcement officers (LEOs) arrive. For public safety, there are two timelines:

1. Mitigate suspect threat.
2. Deliver patients to definitive medical care (emergency department or trauma center).

Having suspect and victim timelines displays the operations for these two competing objectives.

To mitigate suspect threat, LEOs must clear the building and identify any suspects who are mobile. This may require an unknown number of LEOs, therefore every available officer responds and drives in as close as possible to the incident. The unintended consequence is a large number of LE vehicles impeding fire rescue and EMS access to the victims and blocking escape routes if an improvised explosive device is discovered, a hazardous materials incident occurs, or confederates of the suspect initiate sniper fire.

To physiologically stop and begin reversal of the damage from severe gunshot wounds (GSW) necessitates acquiring an airway and stopping blood loss, taking the casualty out of danger, and/or bringing fire rescue and EMS into the hostile area. The study of competing time lines illustrates this conflict. The LE timeline shows the attempts to intercept the suspect timeline to stop the injury process while at the same time intercepting the victim timeline to reverse the injury process. Analysis of the efficacy of public safety’s response to the rapid wounding and killing in this type of event must include the limits of time for those exposed to the threat of the suspect(s) and the limited time available to law enforcement, fire and EMS working to save lives

***The suspect(s)’s timeline*.** The assailant(s) intent is to inflict the maximum number of wounds and deaths on the greatest number of people in the shortest period of time, prior to law enforcement’s arrival on-scene. The duration of these events has progressively decreased since the hours-long April 20, 1999 school attack by two suspects in Littleton, Colorado, at Columbine High School. Attackers typically study past Active Shooter incidents to improve their tactics including the common observation that law enforcement intervention generally takes less than five minutes after the first 911 call is received. Attackers have prepared for this by developing techniques to delay law enforcement or increasing the speed of firing their weapons. For example, at Virginia Tech, April 16, 2007, the suspect isolated a building by securely chaining doors impeding law enforcement entry, allowing for more injuries at an estimated rate of one round per second when shooting inside a classroom. The suspect in the Aurora movie theater shooting on July 20, 2012, murdered 12 and wounded 70 people in what is estimated to have been approximately one minute from start to finish.

The police timeline. Shortly following the Columbine High School shooting, Jeffrey Martin and George T. Williams (1999) described “active shooters who are armed, have been shooting, and continue to shoot even in the presence of responding police.” They recommended that law enforcement officers rapidly enter the scene to interdict the suspect and mitigate the threat of the shooting. Many Active Shooter response models now call for single officer entry through multiple ingress points to decrease the time the suspect(s) has contact with potential victims. The police timeline has consistently been considered to be the time to contact with the suspect. Rapid entry of officers

and diversion of the suspect's attention from unarmed victims, directing the attention of the suspects toward the police, will lower the casualty numbers.

The live victims' timeline. The US military defines sudden death from penetrating trauma as death within six minutes. The wounded are divided by EMS response protocols into three categories, those for *immediate transport* need immediate medical care, those in need of medical care but are capable of surviving for a period of hours (*delayed transport*), and those who suffered *minor* injuries who can tolerate extended delays to reach medical care. Each victim's Active Shooter timeline begins at the point of wounding and, depending upon the severity of the wound, ends either in death or upon the delivery to an emergency department or trauma center. The victim's timeline includes the first wound, the law enforcement response mitigating the suspect's threat, the transfer of the victim to EMS care, and the initiation of definitive medical care. Every delay in the chain of this timeline sequence creates medical uncertainty for those in immediate need of definitive care and will influence survival.

Historically, the primary goal for law enforcement active shooter response models is to "stop the killing." This translates into field operations as stopping or shooting the suspect(s). In the nearly two decades since Columbine, following the interdiction and mitigation of the suspect's threat, the practical needs of the wounded have created an informal secondary goal that law enforcement has not traditionally recognized. It is common practice that, once the suspect threat resolves, responding officers urgently call for the EMS response into a safe scene. In the Aurora movie theater shooting (TriData 2014,48-50), confusion regarding scene safety between law enforcement, the fire department and EMS on either side of the theater led to law enforcement officers using their vehicles to transport gunshot wound victims to area hospitals.

With more experience in these events, law enforcement now recognizes the need to facilitate the rapid transfer of critical gunshot victims to EMS personnel. EMS can then initiate Mass Casualty Incident protocols for triage, treatment, and transport of patients to definitive medical care. To achieve this, law enforcement is increasingly recognizing the importance of balancing the dual timelines involved in life-saving: 1) mitigation of the suspect threat and 2) the delivery of patient to definitive medical care facility.

This will also involve actions on the part of the fire department and EMS to close the distance between a safe triage area and the warm casualty collection point.

CONSIDERATIONS

LAW ENFORCEMENT

- The law enforcement focus toward earlier movement of casualties to EMS may place a burden on the fire department and EMS to move into the warm zone or have available specially trained paramedics (Mechem 2015).
  - *This will also involve actions on the part of the fire department and EMS to reduce the distance between a safe triage area and the warm casualty collection point.*
- Law Enforcement vehicles impeded access to the command post (CP) for unified command and to the triage site for medical care.
  - *Referral of this situation to law enforcement to consider methods to regulate the influx placement of vehicles.*

- Fire department assets responded into the warm zone for Triage A and for a team response to the casualty collection point.
  – *Work with law enforcement to identify methods to use fire apparatus as ballistic protection in or near a warm zone.*
- Designated Protective Services Details (force protection) were withdrawn from FS 231 to allow officers to respond for assistance at an active shootout with suspects.
  – *Meet with law enforcement to identify a Designated Protective Services Detail to train with fire companies for placement of apparatus, personal protection (behavioral), and designated protection of fire department assets.*

GAP ANALYSIS: LOCAL STANDARDS

SAN BERNARDINO CITY FIRE DEPARTMENT STANDARDS

SBFD Internal Standard		Did Comply	Did Not Comply
**MCI documentation<br>Multi-casualty Incidents 30.51**	Forms EMSA 01A	Yes	
	Patient log	Yes	
**Standard operational procedure<br>Incident Command System 30.22**	Command Post	Yes	
	Staff assignments	Yes	
	Liaison with other agencies	Yes	
	Alternate radio channels	Yes	
**Standard operational procedure<br>Command operations 30.23**	Communication	Yes	
	Command	Yes	
	Evaluation	Yes	

ICEMA STANDARDS

Met all standards except:

Did not meet Determination of death standard or use of START standard.

Standard		Did Comply	Did Not Comply
**Trauma Triage Criteria**	Reference 15030	12	
**Determination of Death Criteria**	ICEMA Reference 12010 Obvious		14
	ICEMA Reference 15030 Trauma		14
**Multi-Casualty Incident**	Reference 5050	Yes	
	Declare MCI	ME 231	
	ReddiNet	ME 231	
	IC	BC 602	

Standard		Did Comply	Did Not Comply
	Operations Chief	BC 605	
	Staging ¼ mile	ME 212 Colton	
**Triage**		M231	
**Triage A**		M231 assist	
		Tactical Medic	
**Triage B**		San Manuel	
		ME 241	
		ME 75	
**Triage B**		ME 252LL	
		ME 541 Cal Fire	
**Treatment**		MT 221 Leader	
		ME 224 Immediate	
		MT 224	
**Transportation**		ME 229	
		AMR SUP	
**Morgue**		MT 221	
**Use of Start Triage**		0	20
**Med Comm**		ME 231	
**Classification of patients**		Yes	
**Number of Adults**		28	
**Number of deceased**		14	
**Medical Control "Prior to contact"**		Yes	
**Field documentation**		Yes	
**Med Comm Log**		ME 231	
**Triage Tag Number**	Triage A	11	1
	Triage B	5	
	Probation	1	

20 patients

All met "trauma criteria"

Reference 15030

Trauma Triage Criteria

ANATOMIC INDICATORS

- *Penetrating injuries to head, neck, torso and extremities proximal to the knee or elbow.*

14 deceased

None of the deceased met “Obvious Death Criteria”

ICEMA Reference 12010

DETERMINATION OF DEATH CRITERIA

- Decomposition.
- Obvious signs of rigor mortis such as rigidity or stiffening of muscular tissues and joints in the body, which occurs any time after death and usually appears in the head, face and neck muscles first.
- Obvious signs of venous pooling in dependent body parts, lividity such as mottled bluish-tinged discoloration of the skin, often accompanied by cold extremities.
- Decapitation.
- Incineration of the torso and/or head.
- Massive crush injury.
- Penetrating injury with evisceration of the heart, and/or brain.
- Gross dismemberment of the trunk.

None of the deceased met “Penetrating Trauma Arrest”

ICEMA Reference 15030

DETERMINATION OF DEATH CRITERIA

If the patient does not meet the *“Obvious Death Criteria”* in the ICEMA Reference #12010 - Determination of Death on Scene, contact the Trauma base hospital for determination of death on scene for those patients who suffer a traumatic cardiac arrest in the setting of penetrating trauma with documented asystole in at least two (2) leads, and no reported vital signs (palpable pulse and/or spontaneous respirations) during the EMS encounter with the patient.

DETERMINATION OF DEATH IN THE 14 DECEASED IN THE IRC

Three separate paramedics entered the warm zone of an active shooter incident, before the room and outside area were fully cleared. They evaluated for signs of life, as described in this AAR. Because of danger to self, they had to exit the room as soon as they had each determined 1) the absence of signs of life, 2) the futility of providing care with severely limited staff and resources, and 3) their personal danger.

SAN BERNARDINO CITY FIRE DEPARTMENT STANDARDS

All pertinent goals met and documents complete
Standards for SBFD
Standards for ICEMA with exception of helicopter transport
(ICEMA EMS standards in medical section)

STANDARD OPERATIONAL PROCEDURE
30.22 Incident Command System
30.23 Command Operations

MCI DOCUMENTATION
30.51 Multi-Casualty Incidents

KEY ICS POSITIONS

- Air ambulance coordinator
- Air operations branch coordinator
- Delayed treatment area manager
- Ground ambulance coordinator
- Captain 229 was working side by side with American Medical Response Supervisor to manage ambulance resources
- Immediate treatment area manager
- MT 221 PM/FF assumed Treatment Unit Leader
- Litter bearer
- Litter bearer manager
- Medical communications coordinator (Med Comm)
- Paramedic/Firefighter from ME 231 made contact with primary Emergency Communications Center (ECC-call sign Comm Center) for San Bernardino County to give notification of MCI and obtain bed availability.
- Medical group/ division supervisor
- MT 221 Captain functioned more as Operations or Medical Group Supervisor
- Minor treatment area manager
- Patient transportation group supervisor
- Triage personnel
- Triage unit leader
- Triage A ME231 was given the pre-designated assignment for the Captain to fill the role of Triage Unit Leader
- Triage B. ME 241 tied in with EMS Coordinator from San Manuel FD (EMS932), who was establishing a Triage B area at the face to face request of SBFD Battalion Chief 605.
- Once all of the triage was completed an ME231 Engineer was assigned as Morgue Manager at the Morgue Tarp, separated from the treatment area.

ICEMA STANDARDS FOR FIRE DEPARTMENT MULTI-CASUALTY INCIDENTS

5050 Multi-Casualty Incident Med Comm

Triage Tags

8070 AIRCRAFT ROTATION POLICY

All EMS Aircraft requests from the field in San Bernardino County will be dispatched by the San Bernardino County COMM Center.

Sheriff's Air Rescue responded to a crime in progress and was not requested by EMS. Once on scene, Air Rescue Paramedics assisted SWAT Medic for tactical medical care.

This policy did not apply.

An EMS Aircraft going to a destination other than the one assigned by Comm Center, will notify COMM Center and the receiving facility. Notification may be made by ground or air crews, whichever is the most expeditious for information to be given to the receiving facility.

The decision to transport two patients occurred during an unexpected pause in ground ambulance arrival. Uncertainty existed whether more victims would be found. Ambulance manager elected to use Sheriff's Air Rescue to conserve ground ambulances and reduce sure to area trauma centers. The pause was about 3 minutes making it impractical to contact COMM Center and await a reply.

Exigencies of the situation precluded contacting COMM Center, a known critically injured patient with uncertain number of patients with an unpredictable time element.

EMS REGULATORY OBLIGATIONS

REGULATORY OBLIGATIONS

START TRIAGE

5050 Medical Response to a Multi-Casualty Incident

The Incident Commander (IC) will assign the first available resource to triage. Adults shall be triaged according to START as outlined in Fire scope.

Not met. START Triage not used. See conclusions and Lessons Learned.

DETERMINATION OF DEATH CRITERIA

12020 “Obvious Death Criteria”

15030 “Penetrating Trauma Arrest”

Contact the Trauma base hospital for determination of death on scene for those patients who suffer a traumatic cardiac arrest in the setting of penetrating trauma with documented asystole in at least two (2) leads, and no reported vital signs (palpable pulse and/or spontaneous respirations) during the EMS encounter with the patient.

Not met. See conclusions and Lessons Learned

MEDICAL RESPONSE TO A MULTI-CASUALTY INCIDENT (REFERENCE 5050)

First arriving resource with the appropriate communications capability shall declare an MCI; establish command, name the incident and request hospital bed availability through the Coordinated Communication Center (CCC).

Met, see below

The Incident Commander (IC) will assign the first available resource to triage. Adults shall be triaged according to START as outlined in Firescope. Pediatric patients shall be triaged according to JumpSTART (see definitions) developed by California Emergency Medical Services for Children.

1. The CCC will confirm patient departure from scene with Med Comm by providing the departure time.
2. The CCC will advise receiving hospitals of the number/categories of patients en route via ReddiNet or other approved method.
3. The CCC will notify all involved hospitals when the MCI is concluded.

GAP ANALYSIS: NATIONAL STRATEGIES FOR PATIENT CARE RESPONSE DURING INTENTIONAL MASS CASUALTY EVENTS

"I couldn't believe we didn't have any injuries that needed a tourniquet, there is such an emphasis put on them for active shooter response…at the end of the day, we had a bunch of torso injuries…"

On-scene Medic

SBFD RESPONSE

What stood out most, besides the effectiveness of the San Bernardino response, was the simplicity of their response system. Their collective reaction to this event appears to be almost second nature. What the interviewers heard repeatedly, while speaking to those that took part in casualty management from the point of wounding was, "We did what we do within our day-to-day response."

It is not uncommon for first responders in San Bernardino to be dispatched to gunshot wound (GSW) calls. A significant number of these calls have two, three, or four patients injured. On December 2, 2015, the first responders performed, for the most part, in the same manner they do on any "day-to-day" GSW call. What changed was that they immediately increased their tempo, resources, and maintained a constant sense of urgency when the incident dictated they do so.

The responders of San Bernardino did not adhere to any theoretical active shooter response model. Instead, they engaged the situation with what they knew and adapted when necessary. They were prepared to deal with whatever they encountered, although during the interviews, many were surprised that the wounding patterns of their casualties did not match what is being taught and written about nationwide.

HARTFORD CONSENSUS AND CONTEXT

In response to the increase in mass casualty and active shooter events, the American College of Surgeons convened, in Hartford, CT, the Joint Committee to Create a National Policy to Enhance Survivability from Intentional Mass Casualty and Active Shooter Events. The recommendations from that committee, now known as the "Hartford Consensus," have shaped national policy for intentional mass casualty and active shooter events. One area of influence is the focus on hemorrhage control based on the American military combat experience (Jacobs et al 2015).

The committee focused on the US military experience treating exsanguinating hemorrhage of an extremity as a preventable cause of death. The use of tourniquets and hemostatic agents contributed to this decrease (Kelly et. Al. 2008; Kotwal et al. 2011). The data used to support the recommendations put forth by the Hartford Consensus in this paper is the same data many would recognize if they follow the Department of Defense's (DoD) Tactical Combat Casualty Care (TCCC) Guidelines. On the surface, it may seem reasonable, to just follow what the military does. TCCC is evidence-based medicine, so the epistemology is inherently present for justification. Over the last 14 years of combat from two theatres of war, the DoD and TCCC committee have amassed an enormous amount of data on injury pathophysiology.

In addition, with the type of weaponry used domestically in the active shooter (AS) arena, such as the well-publicized AR15, the surface-level similarities between civilian and military events appear, at first, to be perfectly aligned to

draw broad based correlations and subsequent recommendations for medical care during AS responses. If that's the case, couldn't we just take this already existing foreign combat data and weave it into the fabric of civilian AS response? Unfortunately, the answer is usually "no."

MILITARY WOUNDING PATTERNS VS. CIVILIAN WOUNDING PATTERNS

It is true that bleeding control is a vital capability for every first responder and first care provider that arrives to any medical scene. Every first responder should be prepared to manage uncontrolled extremity bleeding for every situation, from a vehicle crash to a dynamic act of violence. However, emphasizing the tourniquet as the primary medical treatment needed for an AS event risks misdirecting first responders away from more likely reversible causes of death. The committee extrapolated from the tactical combat situation to tactical domestic situations. No comparison of wound patterns occurred.

Edward Smith, et al. (2016) specifically examined the overall wounding, the fatal wounding, and the incidence of potentially survivable wounds in civilian public mass shootings. Their stated goal was to gain a perspective on civilian fatalities in the same manner that Eastridge, et al., did for the modern battlefield in 2010. When comparing the results, they found that fatalities following civilian public mass shootings "differ from combat fatalities in the mechanism of injury, overall wounding pattern, the fatal wounding pattern, and the percentage of potentially survivable injuries."

In the conclusion of their paper, the authors state, "There are few, if any, deaths from exsanguinating extremity wounds (in active shooting events). We discourage a myopic focus on hemorrhage control for civilians and instead urge that the complete tenets of civilian-based Tactical Emergency Casualty Care (TECC) be implemented across the entire pre-hospital trauma spectrum and further recommend studying the strategy to affirm its benefit." While not discouraging the use of tourniquets or hemostatics, they emphasize that the risk of chest injuries is greater than that of military data. Additional treatment modalities should be equally emphasized for response to the civilian mass shooting.

TCCC guidelines come from injury patterns occurring in military combat operations. Prioritized treatment modalities decrease mortality, focusing on potentially preventable causes of death on the *battlefield*. Rapid identification and management of the extremity hemorrhage is central to the TCCC guidelines. Within the military dataset, exsanguination continues to reign as the number one preventable cause of *battlefield* death. Tourniquets and hemostatics have saved thousands of lives since their integration. The use of military data explains the Hartford Consensus views that hemorrhage control is second only to engaging and defeating the shooter as key to improving the survival of victims of AS incidents. They describe external hemorrhage control as "the critical step" in eliminating preventable prehospital death (Smith 2016).

Moving forward, however, it is crucial that we understand that the context is different between military and civilian injury datasets and response. The TCCC Committee and NAEMT specifically acknowledge, reference, and address this gap between civilian and military prehospital trauma care as the foundation for which the TCCC Guidelines were originally created, and list it as an enabling learning objective within their *Introduction to Tactical Combat Casualty Care* slide deck.

The current data utilized to shape our civilian national medical response to active shooter incidents (from the bystander to the prehospital care provider) is based on military injury data, predominantly from the last 14 years. There is an overwhelming amount of evidence showing the criticality of hemorrhage control for the extremities in

the *military* population. Yet it is also essential to appreciate that the military data does not translate directly to civilian data.

The military data supporting the conclusion for the sole focus on bleeding control assumes two inaccurate premises for civilian application: First, that the injured will be wearing body armor, most likely level IV front and rear plates, a Kevlar helmet, and potentially ballistic collar/throat, side, bicep, and groin protection. The casualty population of injured American Service men and women within the expansive data were wearing body armor and Kevlar helmets, a variable that greatly skews the numbers towards unprotected regions of the human anatomy (extremities). Of these injuries, ground level explosive devices outweigh gunshot wounds dramatically (Eastridge,73.7% Blast/Fragmentation compared to 22.1% GSW).

Although we should recognize that after the Boston Marathon attack, we must be prepared for the use of IEDs, we also need to appreciate that firearms are the predominate weapon of active violence incidents in the United States. Although TCCC was listed as one of the biggest advancements in decreasing mortality in combat, improved PPE was first on the list for good reason (Holcomb, et al. 2006). Due to PPE being absent from the civilian casualty population (we do not send our kids to school in level IV body armor), the relevance of this data must be critically analyzed within the realms of the domestic environment. By adding body armor and associated PPE onto the soldier, the variability of injuries narrows significantly to the unprotected anatomic regions. In contrast, the unprotected civilian anatomy is left wide open. By way of comparison, if we wished to look at decreasing civilian burns in house fires, we would not look to the data of burns experienced by firefighters in full PPE during interior firefighting operations.

Second, the penetrating trauma is occurring within different contexts. We would be remiss to not mention the distance as a contextual reference point for which these injuries occur. "In after-action reviews from combat, reported engagement distances ranged on average from 20-30 meters. Public mass shootings most often occur at a much closer range, most often inside rooms with the rare marauding attack occurring outdoors from a moving platform" (Smith 2016). In the military, the gunfight can have a standoff of greater distance with both parties firing at each other. In the civilian context, it is common that the assailant can get within very close proximity to their victims who are generally not fighting back. With this closer proximity and lack of ballistic armor, the instances of torso and head wounds are dramatically higher.

There appears to be little civilian data available to inform a national policy to enhance survival in domestic mass casualty shooting events. A contributing factor that may be influencing this deficit is confusion between precision and accuracy. Nate Silver (2012) describes it as follows: "Even if the amount of knowledge in the world is increasing, the gap between what we know and what we think we know may be widening. This syndrome is often associated with very precise-seeming predictions that are not at all accurate."

When looking at all the military data and corresponding research papers, there is, without a doubt, *precision*, and within the context of military parameters, it is also *accurate*. When we change the contextual parameters, however, and attempt to translate this data over to civilian mass casualty shooting incidents, the accuracy is lost even when the original data still maintains impressive precision.

The "Stop the Bleed" campaign emerged from the same Hartford Consensus group. Within context, the concept of Stop the Bleed should be a national initiative because hemorrhage currently accounts for a significant amount of deaths every year. There are also first responders who still do not carry tourniquets or hemostatic agents in their gear caches. The friction point is not that hemorrhage isn't important, or worthy of training and protocol. It is that hemorrhage control is still being relayed as one of the primary medical procedures a first responder will utilize during a domestic mass shooting incident. This is not valid, nor is there any data to support this assumption.

The yearly deaths from trauma, specific to hemorrhage, are from a full-spectrum of injuries ranging from industrial incidents to motor vehicle collisions. Many of the first responders interviewed, who were prepared to apply a tourniquet, voiced their surprise, due to all of the published papers concerning AS response, that none of their patients required tourniquet application. They were also surprised at the amount of thoracic injuries they encountered.

In light of the ever-present threat of explosives, and the recent Las Vegas incident where the shooter was firing down from the 32nd floor at very large groups of people, the potential for life threatening extremity hemorrhage is always a possibility. Every first responder should have the appropriate equipment and training to rapidly intervene, with emphasis on treatment capabilities beyond bleeding control as a mandate. This does not negate, however, the fact that thoracic injuries are of paramount importance in the AS arena, that they are more ubiquitous than many of us realize, and that rapid triage and transport is critical to the preservation of life.

FINDINGS: OVERVIEW OF METHODS AND DATA

WOUNDING PATTERN

Fatal versus Non-fatal

	Fatal=14		Non-fatal=15	
	Core	Extremity	Core	Extremity
**Total**	36	14	12	16
**Percentage**	72%	28%	43%	57%
**Extremity wound distribution per victim**		1		1.1
**Chest wounds**	25		8	
**Chest wound distribution per victim**	1.8		0.53	
**Total wounds**	50		28	
**Wounds per victim**	3.6		1.9	

* Core is head, neck, chest, abdomen, and pelvis.

Notes:

- Wounds to the trunk were associated more with fatality.
- Numbers are similar except chest is 25:8 (Fatal: Non-fatal) - Head is 3:0 (Fatal: Non-Fatal).
- Chest is predominant region for fatality. Fatalities received 1.8 chest wounds per victim while survivors received 0.53 chest wounds per victim.

DECEASED

There were five deceased victims whose autopsy face sheet reports suggested that operative repair could be possible. The face sheet does not carry details necessary for a sufficient examination. The five deceased victims had one or more of: lacerations of large vessels, pulpefication of solid organs, or tears and bullet fragments in the airway.

The above reports do not consider environmental time delays as a co-morbidity. The shortest time interval for a patient to leave Triage A, however, was 36 minutes and ambulance transport times averaged 5 to 10 minutes.

DEATH DETERMINATION

Deceased

- 14 on scene, two moved by law enforcement to Triage A.
- Nine with fatal wounds, five with non-survivable wounds.
- All deceased were found originally within the hot zone. The area remained a warm zone until after all casualties had departed the CCP.

ICEMA has two protocols for determining death: 1) “Obvious death” and 2) with penetrating trauma “traumatic arrest with penetrating injury” which requires base hospital physician contact.

DETERMINATION OF DEATH

Medics used the same visual method for determination of death: no spontaneous patient movement, gray skin color, and no visible chest movement. The medic would then feel for a carotid pulse in both arteries one at a time.

In each case several medics re- examined for death determination shortly after a medic made the determination. This occurred two to three times for each of the deceased.

All autopsies were reviewed by the AAR Team and trauma consultants who found fatal wounds or non-survivable wounds in all of the deceased.

The criteria used by the paramedics to determine death was not congruent with the criteria used for determination of death derived from signs of obvious death or penetrating trauma with physician contact. The paramedics who determined the victim was deceased looked for complete lack of movement and then both gray, ashen skin as well as absence of respiratory effort. They would confirm by palpating the carotid artery on one side and then the other side. Determining death required all the elements: no movement, no breathing, gray ashen skin, and no carotid artery pulse. When cross-referencing with START triage, with the absence of respirations after repositioning the airway, the victim was marked deceased.

PATIENT MOVEMENT

SCENE TO CCP

Law enforcement officers and probation officers:

- Moved casualties from the conference room to the casualty collection point (CCP) in the immediately adjacent parking area.
- Obtained vehicles to drive casualties from the CCP to Triage A.

INTER-OPERABILITY

Community law enforcement officers, school law enforcement officers, and probation officers from various jurisdictions rapidly combined to suppress the threat in order to move casualties as early and quickly as possible toward Triage A.

An SBFD team arrived, guarded by a SWAT Medic, in preparation for an additional 20 more casualties. There were no additional casualties, so the fire crew went into the conference room for a secondary triage and confirmation of death.

LAW ENFORCEMENT

- PD incident commander assigned law enforcement officers to move patients as soon as officers were available.
- PD officers quickly learned the purpose and procedure for first aid and moving patients (outside of their routine training).
- The SWAT Medic managed casualty care, delegated tasks, did not attach himself to any victim (The police officers have received this training previously)
- The SWAT Medic shared first aid kit to officers to treat any injury.
- Officers were also given medical kits to provide life-saving interventions (LSI), while loading onto VOO. This medical training was done and rehearsed prior to the event – allowing the single medic the capability of managing the casualty problem and delegating without getting drawn in to a single victim.
- Police officers and probation officers.

– Provided first-aid care from their duty first aid kits.

– Carried casualties to the casualty collection point (CCP) by hand carry and chairs.

– Drove the casualties to Triage A.

TRIAGE

TRIAGE SITES

Triage A

Selected by SWAT Commander and SBCUSD police. SWAT Commander, drawing on AAR for LAX shooting, wanted triage with unimpeded ingress and egress but near the incident location. School police, drawing on experience from active shooter practice drill, also wanted triage near the incident site but also near the golf course which had open space for a helicopter landing zone and a dirt road off the triage site that could be used for emergency evacuation of the triage site.

Truck companies carry larger MCI bags with the colored triage tarps and additional equipment. Triage and treatment teams used salvage tarps when the truck companies found the color-coded triage tarps had been removed prior to this incident for reconfiguration. Chalk marked the triage level for that tarp.

Triage B

San Manuel FD EMS coordinator, after face-to-face request from SBFD BC 605, established a second triage area on north side. This became Triage B. Medic Engine 241 was redirected to this site as it was forming because of a bomb threat near Triage A.

Triage

START Triage was not used by medics. The START system can "become a mindless algorithm that can potentially under-triage and/or fail you and your patient", said by one medic who responded to this incident.

Paramedics used their own factors for triage which are reviewed in the Triage Section later in this report. It is important to note that the factors have the same items in common.

Re-triage was part of routine patient management at the triage area.

INJURY DISCREPANCY: FIELD TO EMERGENCY DEPARTMENT

The medical condition of patients is difficult to determine because evaluating the patient and recording the data takes time away from transporting them to the hospital. By the time the patients reached Triage A, at least one-half hour had passed.

Some paramedics, however, recorded a brief physical exam we can compare with brief hospital notes for the same patient. Of significance is some patients appeared to be doing well but deteriorated at Triage A. It is also significant that some patients evaluated "delay," and were legitimate "delay" patients, were taken to the operating room on admission to the hospital.

No patient suffered any negative effects due to triage evaluation.

MISSED MEDICAL CONDITIONS

Patient: Gunshot wounds, "Delayed," yet not detected in first triage.

Found on "delayed" triage tarp (complained of new onset back pain).

Wounds (2) in back: no visible blood, difficult to detect holes in shirt.

Patient: Superficial facial injury, "Immediate".

Emergency department.

Surgical repair of facial fracture.

Patient: Back (upper) with graze wound, "Delayed".

Emergency department.

Chest tube, endotracheal intubation.

Patient: Suspected hypovolemia, fluid bolus, decrease heart rate, "Immediate".

Emergency department.

Received blood transfusion.

Patient: Abdomen, non-penetrating, shrapnel, "Immediate".

Emergency department.

Operating room, abdominal organ damage.

Patient: Severe bleeding wound, "Immediate".

Emergency department, extremity graze wound.

Discharged from emergency department.

Patient: Went home without care, the next day transported by private vehicle.

Emergency department.

Chest radiograph showed shrapnel in chest.

All medics interviewed had a profound appreciation for re-evaluation of patients. In almost all the medic interviews, the medic described re-evaluating patients, even after another medic had evaluated the patient. After every movement of a patient, it seemed, the patient was evaluated again.

TRIAGE TAGS

Triage tags did not assist in identifying level of triage for this response. Marking the deceased was difficult because of the water conditions in the conference room. The Tactical Medic used medical tape on the wrist of victims determined to be deceased. Water interfered with the tape remaining in place. [The Tactical Medic now keeps in his kit black electrical tape for its function in wet conditions.]

TREATMENT

Basic Life Support (BLS) care and Advanced Live Support (ALS) worked well for all patients.

BLS LEVEL OF CARE

- Supplemental oxygen was used 6/20 patients.
- Hemorrhage responded to pressure bandages.
- Tourniquets are a BLS level but were not used or needed.

ALS LEVEL OF CARE

Crystalloid fluid bolus, 3

Decrease heart rate after fluid bolus, 1 (patient received blood transfusion in emergency department).

No change in exam, 2

Needle thoracostomy with improvement, 2

(A third patient with respiratory findings and graze wound in chest received chest tube and endotracheal intubation in emergency department).

ICEMA Ref. 15010. Respiratory distress.

Indications for needle thoracostomy in trauma include chest trauma with symptomatic respiratory distress.

Medics needed more treatment supplies, which they obtained from the first arriving ambulances but that left the ambulances stripped of their supplies. Suggested that a cache of equipment be available for drop off to large scale incidents.

AMBULANCE

SBFD worked closely with AMR's paramedics who would assist in treatment working alongside SBFD medics. There were some episodes where there was limited voice communication, it was difficult to differentiate if ambulance teams were not accustomed to visual communications.

RECEIVING HOSPITALS

Destination

Patient destinations were noted to be inconsistent and patients arrived at the ED without prior notice. ReddiNet notifies facilities when all patients have been transported off scene and before arrival at facilities.

For MCI’s with over 25 patients there is no standing order with a number of patients that each trauma center would be able to take. For example, each trauma center commits to taking five patients without complete notification in order to relieve demands on treating field personnel.

Triage

Hospitals noted two missing triage tags on victims upon arrival. Triage tags were not tracked once the patients arrived at the ED. EDs did not have designated personnel to collect incoming triage tags for logging and maintenance.

COMMUNICATION

Consideration should be given to;

- ReddiNet polling did not include Riverside County trauma centers.
- EDs reported incomplete notification from field medics regarding patient condition.

In all, hospitals eventually reported that four of 20 patients received from EMS did not have triage tags. SBFD reported one patient did not receive a triage tag in the field and has recorded numbers for three of the four.

TIMES

CASUALTY TIMES

Time to:

Earliest peripheral IV	31 minutes
First EMS transport	38 minutes
First ED arrival	43 minutes

	Casualty Time	Operational Time
Law Enforcement		
Suspect Mitigation	13 minutes	23 minutes
To triage	31 minutes	
Start of care	13 minutes	
Fire Department	31 minutes	18 minutes
Ambulance	35 minutes	47 minutes
EMS	43 minutes	1 hour 15 minutes

WORKING WITH LAW ENFORCEMENT

An active shooter incident is a joint operation with various agencies working together. It is also a convergent operation and, though the agencies may have previously worked together, individuals may not be familiar with each other.

While the fire department has excellent experience working with law enforcement, for this incident, friction developed concerning: vehicle placement / parking, force protection, and the use of the incident command system.

SBFD personnel do not have experience discerning plainclothes law officers from officers in uniform showing a badge. Some officers dressed in a manner the suspects had dressed in.

The Incident Command System (ICS) caused friction when ranking law enforcement officers, who were not familiar with the structure of the ICS, found themselves working in, and leading, ICS.

PARKING

SBFD's response to Triage A was impeded by the large number of law enforcement vehicles. Unfortunately, this resulted in difficulty arriving at Triage A and difficulty initiating unified command (UC) between law enforcement and the fire department.

The congested parking also effected ingress of crews and egress of patients from the IRC building. Fire department assets responded into the warm zone to staff Triage A and for a team response to the casualty collection point.

FORCE PROTECTION

Those in triage/treatment area were concerned about force protection for personnel and victims.

Numerous LEOs on scene and near triage sites were in plain clothes, many were openly brandishing weapons. LEOs also arrived in unmarked vehicles driving around or through the area and triage sites. Fire and EMS personnel remarked they could not distinguish between some plain clothes LEOs and possible perpetrators. The FBI later found the assailants had driven by the area twice after the shooting incident.

The Designated Protective Services Details (force protection) were withdrawn rapidly from FS 231 to allow officers to respond for assistance at an active shootout with suspects.

THREAT, COGNITION, AND AFFECT

Despite the frustration of different missions coming together and opposing apparatus placement practices, no anger was demonstrated by any member at any time.

Though the possibility of direct threat against SBFD personnel was present, it did not influence the performance of any participant.

CISM/ CISD

AVAILABILITY

- Responded chaplains in 90 minutes, CISD by evening.
- Some responders asked if personnel with Type 3 IMT had access to CISD.
- Private ambulance personnel appear to not have access to CISD through their employer. Some hospital programs invited ambulance personnel into the hospital CISD meetings. Ambulance personnel requested participation with SBFD as they feel more connected to SBFD in culture and work activity than with hospitals.

ACCEPTANCE

- Strong to mild acceptance.
- Considered a weakness by some in the department, and they let others know their feelings.
- Some changed their minds after this incident to favor use of CISD.

SESSIONS

- Took place in a variety of settings from private meetings to meetings in public locations with passersby.
- SBFD personnel were sent to another agency, the Sheriff's office, but were not allowed to participate and were excluded from building.

LEADERSHIP *IN EXTREMIS* (IN DANGEROUS CONTEXTS)

Through the narrative, we see the elements of leadership *in extremis* was a natural, and well-developed component of SBFD leadership.

High reliability organizing methods are already present but not in a formal manner:

- The ability to move fluidly between emergency responder and control operator are present. Members seemed to be aware of the distinction between responding to failure and preventing failure. It also appeared to be the norm between the two processes.
- Preoccupation with failure and the vigilance towards vulnerabilities of the system is present and demonstrated in this report.
- Reluctance to simplify was present, members were always looking for other causes of an event and reasons for something more to happen.
- Sensitivity to operations, very clearly the overarching mission was to rescue victims and provide medical care. SBFD carried out this mission despite emergent problems. These problems were not disregarded and did not act as distractions from the mission but were addressed in real time concomitant with mission execution.
- Deference to expertise occurred at every level of command. Clearly, this was not only from respect for each other but from recognition of the abilities and physical position of the individual during the event.

PUBLIC SAFETY DISCIPLINES

In this incident, we can see the principles of each of the public safety disciplines form a basis for action and for incorporating into other disciplines. For example, the law enforcement officers provided emergency medical care in a hazardous environment. While the form of the environment would be recognized by another law enforcement officer, the medical care would not. A paramedic would know how to examine, lift, and carry a disabled patient while the law enforcement officers do not receive this training. Here the law enforcement officers in an unsafe criminal environment used the paramedic approach to meet the objective of a safe patient transfer.

Respect for the boundaries of the various public safety disciplines was also very clear in this response, yet at the same time there was a willingness to cross boundaries in a respectful and proactive manner. When others crossed over into the fire department's expertise, their action and assistance was well received and supported.

TRAINING

- Paramedics, police officers, probation officers, and ambulance crews showed evidence of attending the training programs. The program of instruction, particularly the school active shooter training program, clearly influenced SBFD's performance for this incident.
- The Los Angeles International Airport Shooting After-Action Report influenced several primary actors in this incident.
- Evident in this active shooter incident was the collaborative problem-solving that developed in the school active shooter training of two years earlier. The emphasis on collaborative problem-solving rather than procedural learning paid off.
- The use of rookies to create training programs teaches lifelong learning, self-efficacy, confidence, and respect to the junior first responder. It also brings new information to established personnel.
- Fire personnel mentioned they were aware an IED may have been present but did not describe any behaviors specific to search for, recognition of, or response to an IED.

Fire apparatus were placed for safety in the context of a fire emergency for protection, ingress of other apparatus, and emergency egress of all apparatus.

- There was no placement of fire apparatus with regard to providing ballistic protection to the triage areas, ambulances, fire and EMS personnel, or patients.
- Due to road congestion from law enforcement vehicles, some fire apparatus were left close to or at staging, some drove against traffic, but all fire companies did reach the triage sites.

SBFD has a tradition of ICS and MCI training. SBFD personnel view this as training “since our first day with the chain of action and transport sequence.” “We also keep this fresh with 20 hours of training a month to ensure that lessons learned, and other information is updated, and procedures modified.”

COMMUNICATION

There were radio failures and some of that was due to the task saturation of the individual while some of it was due to unavailability of the radio frequency. There was a large number of units talking over one another on the main Tac Channel, with requests and acknowledgements going unnoticed. Numerous suggestions were made to have a radio for passive monitoring of the police department.

ANALYSIS

INTRODUCTION: CONCEPTS FOR THIS ACTIVE SHOOTER INCIDENT

Concepts for effective operations in a structured environment do not necessarily translate or adapt to the high threat, unstructured environment. We cannot simply describe this incident as an active shooter incident where LE stops the shooters, fire extricates the patient from the scene, and EMS treats and transports the patient to the hospital. If we reduce this incident to serial stages of action, we miss deep and significant actions visible only by nuanced changes in behavior. We fail to recognize the significance of decisions by independent and interdependent individuals who, by necessity, solved problems and anticipated on a small scale with short-time horizons. This was an incident where public safety personnel came together to act faster than any one individual could think and plan. Bruce West describes these ad hoc relationships / behaviors when describing Complexity Theory. Complexity Theory views behaviors and actions as the interrelationships among a great many components parts.(1) It refers to these interrelationships or systems as complex, because it is impossible to fully understand these systems by reducing them to an examination of their constituent parts.(2) Instead, Complexity Theory holds that interactions produce collective behaviors and characteristics that are not exhibited when the components parts are examined individually.(3) This is in contrast with reductionist theories, which seek to comprehend a phenomenon by examining its individual attributes and are insufficient to understand complex networks.

Fundamentally, an Active Shooter incident is a law enforcement response with the fire service and Emergency Medical Services supporting the life-saving mission. The priority of each in saving a life is fluid, overlapping, and simultaneous. The extensive forensic investigation phase, while not a fire department function, may be supported by fire department processes such as a complete Incident Command System (ICS) with an Incident Management Team (IMT).

Common characteristics of public safety incidents, however, contain nonlinearity, unpredictability, uncertainty, and are in close proximity of threat. Public safety, using both affective and cognitive processes, effectively and organically engages these incidents. Public safety leadership may benefit from leadership concepts developed in dangerous contexts compared to existing business models. Public safety personnel become conditioned to regulate the expected impairment of cognitive processes caused by stress and threat.

Despite conditioning, experience, and knowledge, however, overwhelming events can cause psychological injury when the strength and skills to enter a dangerous context are then injured by that same context. The ability to operate in these environments has always increased the risk for these injuries. Historically, the culture of public safety prepared members through social knowledge and storytelling in order to give meaning to events. This has now become the basis for critical incident stress management.

During this incident, members of the San Bernardino City Fire Department (SBFD) connected with San Bernardino Police Department officers and other law enforcement officers to rapidly triage and extract victims of two assailants. SBFD personnel exposed themselves to threat when they operated within warm zones while San Bernardino Police Department officers and county probation officers extracted casualties from the hot zone of the conference room to a hasty casualty collection point (CCP). From the CCP, probation officers drove casualties to the SBFD triage and treatment site using vehicles of opportunity (VOO).

The characteristics of the response emerged from several factors. The drive to save lives caused smaller units of responders to self-organize into a common mission. The ambiguity of the presence of a threat interfered with public

safety personnel identifying a straightforward solution. The lack of necessary information then led participants to improvise toward an effective resolution.

For the victims, environment and time functioned as comorbidities to complicate rescue and treatment of the wounded. Time taken by LE to mitigate the assailant prolonged the time until the patient could reach medical care. As with any comorbidity, time can independently increase mortality or morbidity. The environment may indirectly increase time because of access to the patient or directly affect the medical condition directly such as by rain, temperature, or toxic substances.

Once life-saving operations concluded, law enforcement also had the duty to investigate the criminal incident. This could have taken place without the assistance of the fire service but logistic support from the San Bernardino County Fire Protection District, through the Incident Management Team, greatly assisted investigators to complete their tasks during the multi-day investigation.

Introduction to a few concepts not routinely discussed with public safety will help explain the public safety response, identify lessons learned, and support recommendations. Described above, these include the VUCA-T environment, High Reliability Organizing, culture, threat-cognition-affect, expert skills performance, distinction between public safety disciplines, leadership *in extremis*, and survivability.

EMERGENCY MEDICAL CARE IN THE PUBLIC SAFETY ENVIRONMENT

Resuscitation science is the medical science for treating cardiopulmonary failure or arrest and life-threatening traumatic injury. Emergency medicine, trauma surgery, and critical care share the treatment of this pathophysiology. Death during hospitalization is almost exclusively from pathophysiology, a form of uncontrolled physiology. The healthcare facility environment is safe and well-controlled. Information is relatively independent of context.

In the public safety environment, the environment, itself, is uncontrolled and deadly from uncontrolled behaviors or energy. Adverse or hostile environments contain important information that makes them heavily context dependent. Law Enforcement (LE) generally works with behaviors when the behavior threatens life, such as from crime or psychological disorders. The fire department becomes involved with various forms of energy and technology when they threaten life. This is generally in the form of thermal energy, chemical energy, mechanical energy, electrical energy, or energy from ionizing radiation (some form of nuclear energy).

Threats within the environment from uncontrolled behaviors and energy require a different type of thinking and operational system. Stress strongly impairs cognition in the unconditioned civilian but public safety personnel generally can become conditioned to moderate this and the reflexive threat responses. Perception and our ability to reason creates bounds on rationality and reason. Public safety personnel, with good training and conditioning, become pragmatic and adaptive with a different form of rationality and reason (van Stralen, et al 2017).

An active shooter incident does not follow linear processes. Civilians, LE, fire, and EMS can be killed by the uncontrolled behavior of the suspect, uncontrolled energy from an IED, or uncontrolled physiology from a gunshot wound, or any combination and degree. The adverse or hostile environment impedes care reaching the casualty or evacuation of the casualty. While we can likely survive any one of these determinants, nonlinear interactions markedly decrease our survivability.

This concept is central to understanding how a survivable traumatic injury quickly becomes lethal. Law Enforcement Officers (LEO) cannot ensure safe entry or a safe working environment until they have neutralized the threat. IEDs may be present. Fire personnel do not have the requisite protective or ballistic clothing for entry. Once public safety personnel do reach the casualty, they must direct their efforts to evacuating the casualty. Vigilance for personal safety, protecting the casualty, and evaluation and treatment of the casualty will distract from effective care and require extra personnel.

"Images of the tactical medic or physician intubating with one hand and laying down suppressive fire with the other…is Hollywood, it is not rooted in any kind of operational reality…Get the patient behind cover, have your guys provide security, and treat your patient."

– USSOCOM 18D Medic

There are other limits, also, such as the number of personnel available to support medical treatments, the amount of equipment available, and the lack of a clean or sterile environment. For an active shooter incident, public safety personnel may only be able to acquire and protect the airway, control hemorrhage, maintain circulating blood volume, and provide orthopedic stabilization. Peripheral vascular access limits the volume of intravenous fluids that can be administered. Because paramedics work under delegated medical authority, the amount of care EMS can offer is limited by regulatory agencies and local physician medical control.

Physicians rarely have the requisite conditioning in the cognitive, affective, and psychomotor processes essential for the dynamic, threatening public safety environment. Consequently, physicians place restrictions on what procedures paramedics are allowed to perform.

Airway acquisition is not as straightforward as in the well-controlled healthcare environment. Maintaining the airway in a turbulent, threatening environment without supportive technology creates unreasonable expectations on public safety personnel. Technical solutions exist to improve salvageability, such as endotracheal intubation, end-tidal CO2 monitoring, and videolaryngoscopy.

In the field, blood volume can often only be maintained with crystalloid solutions. Blood products are usually not available. Therefore, to review an autopsy report in light of care in the hospital--with better techniques for vascular access, larger catheters for administering fluids, and the capability of Massive Transfusion Protocols—would seem to create a false debate.

A worthwhile discussion for active shooter incidents would be 1) simple triage by head and trunk over extremity (after chest evaluation with shoulder injuries), 2) move "immediate" to "delayed" by use of thoracentesis and tourniquet, 3) improved and straightforward determination of death, 4) discuss use of vascular access and fluid administration, 5) airway management, 6) mask ventilation of breathing patients by EMTs, and 6) evaluation and management of hemothorax and hemopericardium.

THE VUCA-T ENVIRONMENT AND SELF-ORGANIZING IMPROVISATION

The seminal finding from this AAR is "self-organizing improvisation." The interviews of the first responders to this active shooter incident revealed a normality to how they operated in unison despite working with new partners, the novelty of the event, and operating in ways new to them. This is not a new finding as many interviewees described similar past experiences, starting as rookies or even working alone on their first multi-casualty incident. Our interviewers had also experienced these interactions. It is the magnitude of the San Bernardino response and the

intimacy of shared experience that brought focus to these performances. Self-organizing improvisation has likely existed for generations yet without a name to call it or a language to describe it. As a type of social knowledge, it lay hidden in the cultures of operators who work in this world.

The LE officers and SWAT team organized and improvised solutions to secure the building while at the same time initiating casualty care. Probation officers organized to provide aid to the wounded, extract casualties, and then drive them to Triage A. SBFD organized for a large number of casualties and organized a team to enter the warm zone, improvising as they approached. Then these self-organizing units merged at various times to create a larger self-organized group.

This did not arise from a specific plan or training. We characterize self-organizing improvisation as HRO made visible, emerging from the common culture and leadership of each organization. Therefore, we initially describe self-organizing improvisation before we describe HRO. Before we describe either we must appreciate the environment they operate in, the VUCA-T environment. Then we can discuss the leadership that primed this action, the culture that supports it, and the nature of the human minds that self-organize under direct threat while improvising under uncertainty.

VUCA-T ENVIRONMENT

The US Army characterized the post-Cold War environment as VUCA: Volatile, Uncertain, Complex, and Ambiguous. Personnel serving in both the military and in public safety routinely work in a hazardous, if not hostile, environment, something not always discerned by the civilian sector. For this reason, we call the public safety environment VUCA-T, the T representing threat (van Stralen, et al 2017). Recognition of the characteristics of VUCA-T on operations can assist in preparation for these events and help the public better understand public safety operations.

Public safety professionals routinely respond to incidents noted for complexity, nonlinearity, and the capability to rapidly expand. Some have termed this environment as chaos or the edge of chaos, implying confusion or complete lack of order. More likely, we cannot identify the order, form, or structure of events. The use of VUCA-T to describe these environments is more precise and accurate.

In practical terms, success in entering the VUCA-T environment emerges from acts that create structure where none is visible, and which work toward a common end-state. The end-state is one that is consistent with the understanding of commander's intent: saving lives using the processes of their specific disciplines. Each individual enters into this uncertain, time-compressed, threatening environment by first identifying salient and relevant information. From that small bit of initial information, the public safety individual becomes capable of creating sufficient structure to support and build effective operations.

It is the VUCA-T environment that distinguishes public safety from the civilian sector.

This is not a trivial distinction. Uncertainty is less crucial, time is more of an arrow, and threats are more abstract when at a distance in time and space. "A story always sounds clear enough at a distance, but the nearer you get to the scene of events the vaguer it becomes" (Orwell 2008). The passage of time allows a solution to emerge and hindsight bias to influence thinking. "I would not have done that," "you should have …," "did you think of…?" In the absence of time compression, the observer can evaluate information and alternatives. "Exposure to even mild uncontrollable stress can rapidly impair PFC [prefrontal cortex, cognitive] functions in humans and animals" (Arnsten 2009).

Operations in the VUCA-T environment look unreasonable and appear to be acting from the gut to outsiders (van Stralen et al. 2017). Yet they appear reasonable, if not expected, to those who have crossed this "liminal threshold" (Tempest et al. 2007). Operators can either attempt to accurately describe their actions to those who do not understand, or they can restructure their actions into a form that others will likely accept. Either way we lose information, never achieve fidelity to events, and create an after-action report that contains lists, protocols, algorithms, and new concepts that field operators cannot use. In the VUCA-T environment, “inaccurate models can kill” (Heggie 2012).

“Liminal threshold,” a term borrowed from anthropology (van Stralen et al. 2017 119-121), refers to the sill of a doorway (threshold) marking entry into another room. Liminality as a threshold in anthropology marks the middle stage of passage during a ritual marking a change in an individual’s status. It is characterized by fear from the sensation of death fear, ambiguity from loss of known structure, and absence of supportive others. It is similar in experience to probationary periods in public safety. At the liminal, the individual has left the known world and is about to enter an unknown world.

The VUCA-T environment describes the limits of cognition and cognitive processes. How we overcome these limits, as we learn from this incident, may be through acting as a means of thinking, the affective mental processes, and self-organizing improvisation. The almost kinesthetic amalgamation of the cognitive with the affective processes, central to public safety culture, seems to be the root of self-organizing improvisation.

IMPROVISATION VERSUS FREELANCING

The discussion of improvisation invariably brings into question “freelancing” used in the colloquial sense of someone acting on their own accord. Although this is close to the definition of freelancing, one who acts without personal allegiance, it is not a form of improvisation. Improvisation is to perform without preparation yet within constraints. It is a mindful, creative act rising from the unplanned situation. It is *not* a mindless act of anarchy.

How one experiences a VUCA-T incident differs between those within the incident and those outside the incident’s immediate boundaries. Information within the incident has local context and may be fleeting. Perception is limited by the human reaction to threat, the time-compressed nature of the event, and the perceived urgency of the task. Imminent threats impair cognitive processes.

Nevertheless, members do act locally with local information and, because a threat is in close proximity, rely only on local support. This is of necessity in an environment where demands on performance are high and lives are in balance. In these conditions, first-line responders focus on creating change in their portion of the response.

SELF-ORGANIZATION

Self-organization, a concept from complexity science, is a local nonlinear response to a local situation. All forces that drive self-organization are internal to the system; there are no outside influences or leadership. Information develops from short-feedback loops. In physical or biological systems, the direction of self-organizing moves the system to a minimal energy state. In social animal systems, behaviors influence self-organizing of the group toward a functional goal. Humans have the capacity to direct self-organization in order to mitigate threats. For example, frightened individuals may demonstrate fight, flight, or freeze responses influencing group panic directed toward perceived safety. Public safety personnel, on the other hand, become conditioned through training and experience to modulate fear responses producing the capacity to direct, as a group, a self-organizing system toward a common desired or beneficial goal.

Public safety systems are directed by the on-site leader *within* the event. As each individual in the public safety response coalesces into the function of the team, a degree of their personal survival becomes subsumed into the team's operation. The commander's intent, the individual's knowledge and experience, the limits of perception and cognition, and the local environment create an internal force for direction within the self-organizing process. This mental activity increases team safety as the team moves toward the end-state.

Positive feedback lends itself to growth of the system and continuing in the same direction, more common with certainty or when certainty is achieved. Negative feedback, on the other hand, acts as a safety component by identifying the limits within the environment, where there is danger. Through this feedback created from acting, self-organizing systems can build structure within a disordered environment.

Self-organization is a stabilizing process:

- driven by forces internal to the local situation:
- without direction from direct external forces; and,
- responsive to feedback from the environment.

IMPROVISATION

You cannot improvise unless you have taken some action. Improvisation plays off knowledge, experience, and the immediate past responses to actions (Weick 1998). Improvisation as a knowledge-based tactic or strategy (Rasmussen 1983) for an event that transcends rules and experience (Dreyfus 1997 and 2004) is essential for novel and high-risk incidents. This is the type of event commonly encountered by public safety services in the VUCA-T environment.

Planning and training is often based on the assumptions of the planners and trainers about how a future event will unfold. Linear solutions unintentionally disregard the limits of perception, knowledge, and thinking in the context-dependent, time-compressed, high-threat environment. Training context is typically developed and delivered in an environment that does not accurately reflect the working environment with the risk of creating confidence for administrators and responders that seat time and exposure to ideas can replace experience (Weick 2011). By not recognizing relevant differences, the risk increases that lessons learned in one crisis will be misapplied to another crisis (Moynihan 2009). "One of the ways we become mindless is that we create categories and then become trapped by them" (Langer, 2014).

Improvisation is essential to success in the VUCA-T experience because discrete rules and concepts (tactics, techniques, procedures, and plans) cannot anticipate and therefore cannot match the full spectrum of any but the narrowest of situations or the continuously changing state over time (Rasmussen 1983). Successful field operators "are flexible, can learn and do adapt to the peculiarities of the system, and thus they are expected to plug the holes in the designer's imagination," (Rasmussen 1980, 97). A recent example of this would be the Las Vegas incident where prior best practices could not address key performance parameters for response.

Plans and models cannot be expected to coincide with unexpected events (Weick and Sutcliffe 2011). The unexpected is better represented by concepts such as *outlier* and *black swan.* Considered as a statistical measure, the outlier is too readily considered random and independent and, thus, quickly disregarded. Considered as representative of a process, typical of the public safety mindset, the outlier is recognized as possibly an early herald of system failure. Talebs's concept of "platonic fold," the explosive boundary where the Platonic mindset enters into contact with messy reality, is the gap between what is known and what one thinks is known. A person's mindset can allow this to become dangerously wide (Taleb 2010).

Weick (1993) discussed the Mann Gulch Fire with US Forest Service and US National Park Rangers. He conjectured that the ability of the National Park rangers to self-dispatch to an incident teaches them to evaluate if the structures of the system are functioning and, if not, to improvise. The dispatch of fire companies by the SBFD dispatcher and the self-dispatch of SBFD fire companies in this incident reflects the initiative that supports improvisation.

Improvisation for safe and effective performance in the VUCA-T environment relies on judgment and expert skill performance. This involves acting into the incident, placing value on information in flux, responding to moment to moment changes requiring judgment. In contrast, the competent operator's skill level is static, based on rules and generalizations that depend on known and predictable circumstances. Expert skill performance, on the other hand, creates the adaptations to engage constantly changing, ambiguous, and volatile situations (Dreyfus 1997 and 2004).

SELF-ORGANIZING IMPROVISATION

When we accept competency as the preferred skill and knowledge level we risk the individual becoming trapped in the wrong category or using the "strong-but-wrong rule." Once a person creates or selects a category or rule or begins following a list or algorithm, the person becomes trapped in that definition, a process that creates mindless thinking and action (Langer 2014). Acting from the selected rule might be congruent with the operator's intent but, because of mismatch with the plan, the creation of an inadequate plan, or the use of a wrong rule or procedure, the rule *becomes* wrong and, thus, actions will not achieve their intended result. "When mindless, however, people treat information as though it were context-free – true regardless of circumstances" (Langer, page 5 e-book). This discussion pertains to the VUCA-T environment and not stable situations or problems having limited interventions and answers.

The expert thinks by acting, while the competent or proficient individual thinks by rules. Actions create immediate information that confirms or disconfirms the action and predicates the next action. Through continual action and improvising each response, the individual continually updates information from moment to moment. Rules lower skill performance to the competent level and interfere with an expert's thinking, generally causing a significant deterioration in performance (Dreyfus 1997 and 2004).

Rules are not broken, and they are not "thrown out." The situation simply may not have a corresponding rule, or rules conflict and compete with each other. In the environment without identifiable structure, routinely encountered by public safety personnel, principles guide thinking. As structure is created, it becomes more apparent which rules apply and can then be used.

Self-organizing improvisation begins with the first action by an operator. Until initiated, the dynamic state of the VUCA-T environment will continue to change, expand, and endanger the public. It is in this situation that we see the difference between the expert and the competent from the Dreyfus model of skill acquisition (Dreyfus 1997 and 2004).

- The *expert* acts with uncertain or ambiguous information.
- The *competent* or *proficient* responder categorizes the situation to identify the rules before acting.
- The *competent* responder must first categorize the situation to identify the applicable rule before acting to enact, or change, the situation's trajectory.

LEADERSHIP

Self-organizing improvisation has a unique leadership in that the agency-designated leader may not be present *within* the event. In this case, leadership occurred before the event. This response is an example when a leader-leader, rather than leader-follower, style of leadership exists in the organizational culture. This system intentionally develops leaders within the ranks.

In these leader-leader systems, all the information and structure for leadership development is built into the larger system. During an unexpected event, a leader will emerge within the event. This approach to leadership recognizes that, during an emergency, a leader will always be present. It also recognizes that if something is not known by a member of the team then that information functionally does not exist. That is, it is not available to the member to use to resolve the incident. The same is true if equipment is not present when needed; the member must function within the environment as if that equipment does not exist. Within these teams of leader-leader each member solves the problems they can, assists others, and accepts assistance from others. In practical terms, public safety operates in real time with what it has at the scene at that time, not with what it needs or wishes it had. Leader-leader self-organization is a response to the unexpected.

What may make public safety, and the operational military, unique is that whoever responds is the most qualified. There may be others more qualified but, if they are not available, it is irrelevant. The responding public safety team, then, is the most qualified team for that incident and the public service agency will support that team.

This active shooter incident drove actions (self-improvisation) and the incident drove their solutions (improvisation). This contrasts with abiding by a well-thought out plan, which would have forced the first responders to wait for the accepted thresholds to be identified as having been reached prior to acting.

CREATING STRUCTURE

Personnel from public safety agencies responded to a VUCA-T incident that had no identifiable structure. Upon entering this environment, personnel from the San Bernardino City Fire Department and San Bernardino Police Department, law enforcement officers from allied agencies, including county probation officers, immediately engaged, self-organized, and improvised toward a common purpose of casualty management in the chain of survival: the police mitigating any suspected threat(s) to citizens (stopping the wounding process), transferring the casualties from the conference room fire/EMS as early as possible, and enabling the fire MCI process to rapidly move the casualties to surgical definitive care.

For the San Bernardino City Fire Department, a self-organizing phase occurred when the first units arrived at the staging location and began interacting with events. The Fire IC gave early triage, treatment, and triage assignments, allowing units to materially and psychologically prepare for their duties. During this self-organizing phase, the Incident Commander had a greater influence over a small span of control early in the call. Once released to the triage/treatment site, the units, while still under the command and control of the Fire IC, increased in their self-organization and improvisation. This occurred as the physical distance from the fire IC and the geographic spread increased, diluting direct command and control. The Commander's intent and public safety leadership principles, discussed later in this report, ensured effective indirect command and control.

The structural phase emerges as operators identify what is working to bring control over the incident. As these are identified, more effort is directed toward successful actions. Structure also develops as more resources respond and more varied tasks are acted upon. The Incident Command System now becomes critical for effective command,

control, and communications over a larger event and the efficiently tasking of every public safety discipline's efforts toward the desired end-state.

EXAMPLE

A subtle example of self-organizing improvisation was tarp placement and use at Triage A. Without the designated MCI Tarps, they used salvage tarps marked with chalk for immediate, delayed, minor, and deceased. There was an initial announcement of the designation of what the three tarps. That is, immediate, delayed, and deceased. One medic was not sure if that was ever repeated and the whole system seemed to self-organize. A deceased victim was placed on the immediate tarp. Someone quietly moved the victim to the deceased tarp, out of sight of onlookers. The worst patients ended up on the east end of the immediate tarp and the more stable patients were on the west end of the tarp towards the delayed patients. This occurred spontaneously. It resulted in the most seriously wounded patients being placed adjacent to the incoming ambulances.

HIGH RELIABILITY ORGANIZING AND PUBLIC SAFETY DISCIPLINES

HIGH RELIABILITY ORGANIZING (HRO)

High Reliability Organizations (HRO) were initially described from US Naval air operations, nuclear power plants, and wildland firefighting. HRO describes an organization that operates in an environment where catastrophic failure is expected. For the HRO, however, these failures are rare or nonexistent. Weick and Sutcliffe (2011) identified five principles necessary and sufficient for HRO operations: preoccupation with failure, reluctance to simplify, sensitivity to operations, commitment to resilience, and deference to expertise.

We define the HRO as an organizational methodology that enables an organization to effectively enter and perform in an austere or hazardous environment (van Stralen et al. 2017). We follow the distinction made by Emery Roe and Paul R. Schulman (2015) who identified differences in reliability management between the emergency responder form of HRO, using wildland firefighting operations as their example, and the control operator form of HRO used to manage dangerous technology such as nuclear power. Emergency responders engage a system when it fails while control operators keep the system from failing. HRO, then, can represent a type of organization, a High Reliability Organization, or the methods used to work toward high reliability, High Reliability Organizing.

For the emergency responder, discrepancies and disruptions occur in the environment. For the control operator, they occur in the processes and application of rules. That is, strict adherence to rules is unreliable to the emergency operator but the means for maintaining reliability to the control operator. Deviation from the rules is unreliable, if not unsafe, for the control operator while the emergency responder must improvise when few rules exist to guide operations. This distinction can become an inborn error for the system. For example, when the control operator, who is accustomed to a style which uses rules and protocols to keep the system from failing (such as healthcare preventing error in treatment) works with emergency responders who must self-organize and improvise when the system has failed (as in public safety).

This is not to say the two are incompatible. In reality, this is the duality of HRO to use both methods simultaneously. One of the hallmarks of an HRO is the ability to toggle between the two, even using them simultaneously. Values can shift fluidly as demonstrated by the fire companies holding rigidly at staging despite evidence to move to Triage A, the value of obedience to authority. Yet, on arrival, firefighters showed initiative when they entered the warm zone without obtaining permission and against standard procedures for the fire department. What may appear a conflict to those outside public safety is a mark of adaptability and reliability for public safety personnel.

Public safety operators have a *duty to act* in an emergency and, therefore, are selected and trained to have a *bias for action*. That is, upon encountering a situation during an emergency where the individual can affect change, the operator must make a decision quickly and act toward affecting that change. Public safety response, by its very nature, is a time-limited event in the spectrum of the VUCA-T environment. This bias for action is more than a character trait or sense of duty to others. It is how a person, any human being, thinks in an uncertain situation.

We act in order to think. We act to make sense of an unstructured situation. We act to identify what can be controlled or influenced. "Bias for action," then, is also a “bias for thinking,” understanding, and engagement. Acting in this manner ties together Karl Weick’s concepts of sensemaking which is creating sense of a situation through action and enactment which is to affect change in a situation through acting or not acting. Much of our sensemaking is retrospective. Reviewing the actions of SBFD and public safety as acting in order to make sense, Capt. Chesley “Sully” Sullenberger (personal communication) commented, “After the fact, we try to come up with a rationale to explain why we did what we did."

Capt. Sullenberger further describes this interactive approach: "During a crisis there is not time to think about each specific bit of knowledge or experience that we depend on to make sense of imperfect information and ambiguity. But having those resources immediately accessible in our minds, we use them in a conceptual decision-making process to frame the decision. We essentially quickly come up with a paradigm of how to solve the problem. It is after the fact that we retrospectively begin to attribute specific reasons for the decisions that we made."

Actions are seen to thread through the incident, changing the trajectory of events even as acting clarifies the problem. Specific actions will increase the cues and options available to the responder and, at the same time, make other cues and options less relevant (Weick 1988). This is what Weick calls enactment: we enact the situation. Actions are how we make, or create, sense in the VUCA-T environment. We act and evaluate the response to our action, then improvise or modify our next action based on that immediate result.

HRO describes an organizing method of sensemaking and enactment. We construct our sense of the situation and give it meaning through our interaction with the situation. This is the HRO process of sensemaking. These interactions not only create sensemaking, they are directed toward resolution of the situation. Action-based sensemaking enacts change (the HRO process of enactment).

Enactment and sensemaking are common to all organizations and all human being’s lives. Public safety, though, relies more heavily on interacting with the VUCA-T environment and with people (bystanders, victims, and other public safety personnel) in their response to emergencies, both large and small. As noted previously, Weick and Sutcliffe (2001) identified five principles that distinguish HROs from other organizations: the preoccupation with failure, the reluctance to simplify, the sensitivity to operations, the commitment to resilience, and the deference to expertise.

> ***Preoccupation with failure;*** This is a vigilance toward a system’s vulnerability and the early engagement of problems, noticing adverse change before it begins to build into a threatening problem.
>
> ***Reluctance to simplify;*** This is reluctance to accept simplifications, the incident is always far more complex than what is noted on initial observation. HRO's are also suspicious of simplicity because that represents a loss of information; complexity is where information lies. HROs will simplify but are reluctant to do so. Simplifying the ahistoric event can be catastrophic. Simplifying the event can be compared to stereotyping an individual where accurate context and nuance is lost.

Sensitivity to operations; There is a priority of addressing local discrepancies, disturbances, and interruptions as they arise *while still* maintaining strategic operations.

Deference to expertise; Rather than deferring solely to rank or an outside expert, the organization recognizes the importance of the local knowledge gained from interacting with the situation. Command may have a better understanding of the larger context and global operation due to distance from the situation. However, those individuals directly interacting with and having relevant knowledge and experience to respond to the specific local problem now possess valuable local information. They may know the problem with sufficient intimacy for more effective or timely action. Deference here does not mean "*must* defer," it is more "*attention* to."

Commitment to resilience; Support of the continued, open-ended working of a problem until resolution is achieved.

While necessary and sufficient to identify high reliability organizing, the five principles do not provide a motive for individuals to engage a situation or a process for an organization to achieve HRO. This is in the "affective" domain of attitudes, values, and motivation; the value of information and goals and how that value is situational; and the regulation of the sensation of fear and modulation physiological threat responses. It uses a system of short-feedback decision-making such as John Boyd's OODA Loop rather than protocols, algorithms, and decision trees.

PUBLIC SAFETY DISCIPLINES

Although the fire service, law enforcement, and EMS are public safety disciplines, they operate in similar environments but with distinctly different threats. Thus, they have different missions and operations.

Threat suppression, or threat mitigation, terminology often appears in AS response guidelines and training, without defining the various threats and how each discipline of first responders interacts with these threats. This creates confusion when integrating disciplines. While all Public Safety disciplines operate in VUCA-T environments, they engage different threats, operate on different timescales, and act in a different space.

- Law Enforcement engages unpredictable, uncontrolled behavior that is intentional, thinking, and mobile; threats change in less than a second. The *behavioral threat* can expand indefinitely in size, scope, and time. Mitigation of threat is attained through containment, isolating and ending the capability of the threat to continue to injure others, and ensuring every physical space is safe and secure. The response tends to be individual units acting with some degree of independence, aggregating into ad hoc teams that may exist for the duration of the incident or dissolve and then rejoin with other officers for specific, limited tasks.
- Fire department engages *physical threats*, generally uncontrolled energy in the five more common forms (thermal, chemical, mechanical, electrical, and ionizing radiation). The uncontrolled dissipation of energy or uncontrolled transformation of energy between forms, following the laws of thermodynamics, damages property, injures people, and kills. This is finite, ending when the energy source becomes depleted, is not intentional in that energy does not think or plan, and, with the exception of explosions, changes over minutes. The fire department achieves containment by direct extinguishment of the threat or constructing an environmental perimeter. Units are groups of team members formed into individual companies with organized aggregation through addition of like or different companies, all having interdependent function.
- Emergency Medical Services (EMS) engage immediate *physiological threats* to health through medical treatment of unstable, life-threatening medical conditions. This care is given in the public safety

environment where the environment, itself, can act as a distinct pathology. Generally, EMS works with teams of two operators supported by others as needed. Authorization for the more critical medical treatments and procedures derives from delegated medical authority using protocols and algorithms. (In EMS, delegated medical authority allows paramedics to treat specified medical conditions using specified medications and medical procedures otherwise legally reserved for physicians.) A more rigid, vertical hierarchy can result that, while decreasing unsafe deviations, also reduces adaptability. Fortunately, most medical care in the AS incident is at the level of first aid or EMT care.

Describing the safety disciplines as discrete entities, without acknowledging the characteristics of each threat, makes the differences between disciplines appear trite. Each specialty looks at the situation from the knowledge of their discipline and their experience in that environment:

- Law Enforcement may view the situation as, *A victim will die, or more people will become victims, unless we stop the assailant.*
- The Fire Service may instead say, *A person may die unless our team extricates the casualty from the confined space.*
- EMS may look at it as, *the person will die unless we acquire and protect the airway to ventilate the lungs.* Or*, the person will die if the team cannot begin resuscitation.*

All are correct.

Crossing the boundaries of disciplines demonstrates the richness of the SBFD, SBPD, and probation officers' response to this active shooter incident. The discipline accustomed to stopping the assailant is instead busy stopping the bleeding or driving a dying victim to safety. The team that normally extricates casualties is instead entering a room that is technically in a hot zone. To know what the person will die from, in these scenarios, crosses disciplines and necessitates cross-training, mutual experience, shared language, deference to expertise, and, most of all, respectful communication when there is insufficient time to communicate.

THREAT, COGNITION, AND AFFECT

COGNITIVE AND AFFECTIVE PROCESSES

To work in the VUCA-T environment we must balance threat, cognition, and affect. Threat is an automatic perception that we cannot prevent because it operates under the level of our awareness through part of the brain called the amygdala. Threat is represented by the fear reflexes of fight, flight, and freeze. Cognition describes the "cognitive" processes of the brain which include thinking and the executive functions controlling or influencing perception, decision-making, and action. Affect refers to the processes associated with emotion.

The ability to effectively use affective processes in combination with cognitive processes such as reasoning distinguishes public safety personnel from people in the public sector. Affective processes consist of 1) attitudes, motivation, and values; 2) the value that a person places on information and goals in a particular situation; and, 3) the ability to self-regulate fear and threat responses (fight, flight, and freeze). All three processes, the cognitive processes, the reflexive threat responses, and the affective processes, are essential for public safety personnel to operate in the VUCA-T environment.

A different type of conditioning seems to take hold when we act in the face of threats. People are attracted to the organization and clarity of cognitive decision-making (objective, linear, predictable, with known biases, and heuristics. Professionals take pride in critical thinking, which uses linear thinking and logic, avoids fallacies, and insists that hypotheses are drawn from the facts. In live-or-die situations, threats impair cognitive processes and we must place value on information depending on context and change the value of information as the context changes. This is only a part of the affective domain. The most vital aspect for a public safety emergency is modulation of threat responses and emotion (van Stralen et al. 2017).

CRITICAL INCIDENT STRESS MANAGEMENT

The high-altitude mountain climber has the equipment, training, and mindset that allows the climber to enter a hostile environment. By entering this environment, the mountain climber becomes exposed to ever greater hazards that can injure or kill. In a similar manner, public safety personnel have the equipment, training, mindset, and the ability to regulate threat reflexes. This allows public safety personnel to enter the VUCA-T environment.

We expect extreme activities to have unique injuries associated with that activity. Injuries can come from the environment of the activity (concussions in football) or from extreme physical conditioning (torn muscles and ligaments in track and field). To reduce these the elite athlete turns to coaches for better technique and trainers for medical assistance. In public safety, our leaders act as coaches and mental health professionals (MHP) act as trainers.

Public safety injuries are both musculoskeletal and psychological. The psychological strengths that permit entry into dangerous contexts can also become injured from the context. MHPs are educated and experienced to sort these injuries out and develop a treatment plan.

It is difficult to identify when one has crossed the threshold of a known but unseen threat. Despite preparation, training, experience, and equipment the threshold is not only individual and context dependent, it is also relative for a specific individual at a specific time and situation. This is true of the SCUBA diver for the bends, the high-altitude mountaineer for hypoxia, and the winter sportsman for hypothermia. This is also true for public safety personnel with the mindset, training, and experience that prepared and conditioned them to cross the liminal threshold into a time-compressed, hazardous environment without identifiable structure. This is the unseen threat of public safety. In these situations, the ability to modulate the amygdala in order to operate in high-threat situations also exposes the individual to the possibility of psychological injury to the amygdala. This is the circumstance for Critical Incident Stress.

The use of Critical Incident Stress Management (CISM) or Critical Incident Stress Debriefings (CISD) does not reflect weakness but, rather, it is a sign of strength to have had such severe exposure. CISD and CISM are supportive, not treatments, and therefore may not work for everyone. But no one should be discouraged from making use of these aids. To some extent, the effects of these events can affect morale.

Kathy Platoni, Psy.D. (personal communication) observed:

> It's not the degree or the training that makes any one of these first responders effective. This falls upon experience, trust, and rapport. For any MHP who renders treatment to [public safety personnel], they must have taken the time to obtain some degree of exposure and experience in law enforcement in order to be a valuable resource. [This includes] both having dealt with and treating trauma, tragedy, critical incident stress, line of duty injuries and fatalities, poor and inadequate leadership, domestic violence, the impact of [public safety] stress on the family, and the unique aspects of [public safety] stress. MHPs must also be good

"generalists" in terms of providing treatment for the wide range of issues pertaining to the human condition, particularly as this applies to [public safety].

There is far more to receiving mental health services than just "dumping your load." Learning to cope more effectively with the rigors and demands of the job is only a small part of service provision. The MHP should also be adept at providing educational services to the agency and to train officers and agents in such topics as critical incident stress management, coping with departmental leadership issues, surviving deadly force encounters, peer support training and supervision, stress management, grief and loss, LOD issues and the like. There is an ever-present stigma associated with MH services and the fears that too many public safety personnel believe with respect to obtaining them. If the body breaks down, most of us would not hesitate to seek medical intervention. Your head is not disconnected from your body-----the same applies.

Recognize that from a psychological perspective, much of what you are confronting involves *normal reactions to exceptionally abnormal circumstances that have the potential to cause psychological injuries*, unnecessary suffering and a vast human toll. Have the courage of conviction and the strength of character to realize that alone, none of us has all the answers.

The psychological ability to enter the public safety incident is also the ability to sustain psychological injury.

PSYCHONEUROLOGY

To appreciate the mental capabilities demonstrated during this active shooter incident it is useful to distinguish threat, fear, and anxiety from each other. These objective threat *responses* are the commonly known fight, flight, and freeze working through the amygdala. The subjective fear *feelings* are fear and anxiety working in the frontal cortex. The mechanism for the unconscious response to threat is not the same as the mechanism for the subjective feelings of fear or anxiety (LeDoux 2014). We find useful LeDoux and Pine's (2016) distinction between the objective threat response, the subjective feeling of fear, and the subjective experience of anxiety.

- The threat response is subconscious, objectively observable, a suite of reflexive responses, primarily subcortical and amygdala, and primarily occurs when the source of harm is imminent.
- The mental state of fear, as a feeling, is conscious, subjective, primarily cortical, and develops when the source of harm is near.
- The mental state of anxiety, as a feeling, is conscious, subjective, primarily cortical, and develops when the source of harm is uncertain or distant in space or time.

The ability to regulate the threat reflexes of fight, flight, and freeze is vital to public safety operations. No one is certain how this ability to regulate becomes conditioned, but it is clearly present in this incident. We do know that the brain region responsible, the cingulate cortex, is a specific brain region capable of modulating the fear and emotional responses caused by the amygdala. The cingulate cortex also mediates error recognition and adaptive decision making.

The amygdala is also responsible for emotional memory, a form of memory that is unique because, from a single experience, a response is learned permanently and becomes nearly reflexive in nature. This has been identified as giving a survival advantage to wild animals. In humans, it can be protective, or, along a spectrum, it is debilitating.

We see it in the grumpy old-timer who seems to see things before they happen, but we also see it as the physiological basis of post-traumatic stress.

Severe memories interfere with performance because of the learned reflex when a similar incident too strongly triggers a response that is not appropriate for the situation. "The trigger is today but the response is from yesterday."

LIMINALITY

Structure in the VUCA-T environment is not detectable, and an unstructured environment is threatening. The lack of structure creates a threshold that, when crossed with sufficient frequency, conditions the individual to a different way of operating and thinking (Tempest et al. 2007). Anthropology describes this as a liminal threshold, but it also describes extreme events as occurred at the active shooter incident. It helps explain some of the difficulty communicating between public safety and the civilian sector (Tempest et al. 2007). An extreme liminal event is associated with post-traumatic stress (Morris 2015).

Thus, there are two uses of the liminal concept in public safety. This can represent an extreme event when crossing the liminal threshold suddenly places the individual into an unstructured environment where known rules not only do not apply, they are unreliable and may be counter-productive. It is this sense that the liminal event is associated with Critical Incident Stress and Post-Traumatic Stress (Morris 2015).

This response is not only found in humans, a similar response is found in prey and predator mammals (van Stralen et al. 2017, 344). Learning to identify life-threat from a single experience (emotional memory) improves survival in the wild. Ecologists have also identified the effects of this traumatic stress in wild animals as being similar to post-traumatic stress in humans (Boonstra 2013). This is due to the need for prey species to rapidly learn predator threats without the lengthy time period needed for learning with operant or classical (Pavlovian) conditioning. Because this neural circuitry is conserved in animals through predator-prey interactions, it is likely adaptive.

The liminal space, a second form of the concept of liminality, describes an environment of extremes where structure and rules are not known or recognizable by outsiders. This is the meaning used by Tempest et al. (2007) when they described high-altitude mountaineering on Mt. Everest. The danger here is not to those acculturated to the environment, nor even to sojourners who enter the environment under the guidance of mountaineers. Rather, the danger is from novice sojourners who maintain their beliefs and behaviors in the liminal space. This is readily recognizable when public safety personnel explain operations or when civilians such as regulators or healthcare professionals prescribe procedures for use in the liminal space of the public safety environment. This relates to more than observation, it is an embodied set of attitudes, values, and judgment described by HRO.

A consequence of learning to operate in the environment within the liminal space is difficulty explaining judgment and actions to others. The public sector, and even some emergency personnel who have not experienced these events, are outside this environment and will use their beliefs, plans, and models despite not being tested in a dangerous environment. This is particularly true for the level of liminal event that creates critical incident stress. "Insensitive comments from a culture that is perceived as anything but compassionate are a tremendously destructive force," Kathy Platoni, Psy.D., DAIPM, FAIS, COL (RET), US Army (personal communication).

Peer support, then, becomes an important component of critical incident stress management (CISM). These are the people who have shared these extreme experiences. Kathy Platoni (personal communication) identified advantages of this type of peer support. "You ALWAYS have control over how you treat one another. Be there for each other through the worst of times. Be careful about what you say in response, even in jest. Be the kind of person who is

there to support and not to malign…this could be you on the receiving end of bad blood one day. In the aftermath of traumatic events, seek the support of your most trusted colleagues. Remain nonjudgmental at all times.

PERSONAL NARRATIVES AND THE CRITICAL INCIDENT

Stories describing these experiences across the liminal spectrum educate and emotionally prepare the novice for extreme environments. They are a part of the reappraisal necessary for personal growth, identify the values and attitudes for embodiment and give meaning to what public safety is. Stories show the novice who the group is that they are a part of, and what their place is within that group. (van Stralen et al. 2017, 266, 327-329).

There is evidence that, with time, these experiences become internalized and contribute to personal growth, possibly through the sharing of meaningful stories (Campbell 1976; Greenberg and Arndt 2011; Tedeschi and Calhoun 2004, Bonanno 2004).

Highly stressful events require a restructuring of our world view. This restructuring can take either a negative or positive form. After such an event, emotional memory can trigger strongly negative feelings and actions. Over time (months to years), other characteristics may develop such as strength from the recognition of vulnerability, the acceptance of the novel, a feeling of greater connection to others, and the giving of personal values to priorities (Calhoun and Tedeschi 2014).

Surveys of people who have faced major life challenges have identified several characteristics described as post-traumatic growth, or PTG (Tedeschi and Calhoun 2004). People who experience PTG are more likely to embrace novelty, are more open to experience, and are more accepting of paradox. These people use paradox for synthesis to balance order and chaos and to integrate complexity (Calhoun and Tedeschi 2014). People recognize that the personal strength they acquired was because of their experience of vulnerability.

PTG also contributes to resilience, which can be resilience on three dimensions: reaching the outcome, dynamic interactions between risk and protective factors, or reconfiguring one's life (LePore and Revenson 2014). What is important to someone after experiencing trauma often differs from what was important before the trauma. What is important before the trauma is commonly theoretical or conceptual, while what is important after trauma is perceptual and functional.

Public safety transmits social knowledge through stories that embody what it is to be a firefighter or a police officer. These stories give meaning to what appears to be an irrational world, offer error wisdom (Reason 2004) to recover from mistakes, and guide application of general principles to particular situations. To outsiders these are "war stories" and "anecdotes" having little value. In public safety, they carry meaning, demonstrate a principle or truth, prepare the novice for these events, and transmit the social norms of public safety operations. After novices engage an emergency, these stories contribute to their new self-narrative, thus contributing to their resilience (Neimeyer 2014).

These stories are sensemaking after the event to give meaning that links events to a person's personality, identity, and personal growth. The personal narrative that develops after an extreme event can incorporate these deficits into learning and growth or it can become negative, leading to a decrease in self-worth and impaired resilience. Reappraisal changes the way we perceive situations and can decrease the emotional impact, for example by reinterpreting an emotional event in more objective terms. Using reappraisal as a regulatory strategy contributes to this resilience (Bonanno and Burton 2013).

A discrepancy between the individual's appraised situational meaning and global meaning can drive post-event distress. It is this distress that drives our efforts to make sense of the situation, accept the situation, or reevaluate our personal identity or beliefs. Reappraisal as retrospective sensemaking integrates the events, causes, and meanings of the experience (Park 2010).

As social knowledge, the more inexperienced individual in public safety can develop meaning. He or she can learn that even in utter destruction and extreme loss, firefighters, police officers, and medics more than keep the loss from being worse- they save lives and properties even when not visible and they stood by citizens when no one else could. These stories, far from being war stories or anecdotes, are part of the creation of one's identity and starts the embodiment of public safety values and attitudes. They form the basis of the resilience necessary to enter dangerous environments and also support post-traumatic growth.

STRESS AND MEMORY

Stress and threat not only impair memory recall (freeze or confusion) but they also impair memory formation. Cortisol mediates both memory deficits. Two people standing side-by-side while engaging a chaotic incident will have distinctly different experiences and create, or fail to create, different memories to produce normal and expected gaps in their recitations of the events. Cortisol, released from fear or stress, blocks memory formation. Because different aspects of the incident affect individuals differently, there will be different parts of the event each person *does not* remember. The sense they make of the incident is transient and will never again exist.

What is recorded in memory does not represent what participants factually experienced but what their embedded and highly emotional memories cause them to believe they experienced. These embedded memories are susceptible to change. We do not remember the incident we actually experienced as it happened.

An example of this is found in the memory of a shooting victim who participated in a television documentary. She was critically injured in the shooting and in her on-camera interview, stated the following:

> Victim: *"They took us across the street to the triage, and I was placed on the tarp and I look to the right and there were two bodies covered, and then I looked to the left and I watched two more of my coworkers pass away, so…"*
>
> Interviewer: "*Right there?"*
>
> Victim: *"Right there on the tarp, ya, so um, once we were out of the room we were still having to deal with the aftermath and still dealing with death."*
>
> *Showtime Networks, Inc. "Active Shooter: America Under Fire, San Bernardino, California"*

Everyone who was living when they left the conference room survived the incident. Autopsy records from the San Bernardino Coroner's Office confirm the location of death, nine deceased were found in the conference room and three deceased were found outside the doors of the conference room. The two deceased at the triage/treatment site had been moved there in haste by law enforcement officers prior to the SWAT medic organizing the initial extraction of victims.

This victim's very vivid and terrible memory of her experience and its impact upon her life is an example of how the memory of every person is affected by their immersion into a VUCA-T incident. This is not intentional on the part of any individual. Memories are not photographs, though they may seem as vivid. While each of our stories is an

accurate representation of our experience, our memories, and the stories on which they are based, are a less reliable record of the facts of any event than we often appreciate.

EXPERT TRAINING AND PERFORMANCE

Training programs improve procedural memory through repetition to make the routine become automatic. When automatic, the process or procedure requires little attention, freeing cognitive resources for problem solving. This is the purpose of “instinctive reaction training” found in leadership *in extremis* (Dixon et al. 2016) and the purpose behind Rasmussen’s (1983) skill-based behavior.

TRAINING

Another approach for training, one that contributed to the performance of the San Bernardino City Fire Department (SBFD) and San Bernardino Police Department (SBPD) personnel in this incident, is to train for collaborative problem-solving. For example, in their 2013 training session for an active shooter at a school, rather than learning the “best” method to extract and transport patients to the triage/treatment site, the training session evolved. Participants collaborated to identify how to evacuate casualties.

SCHOOL ACTIVE SHOOTER TRAINING, 2013

The San Bernardino City Unified School District (SBCUSD) Police Department and SBPD conducted a joint “active shooter on campus” training exercise at Indian Springs High School. Numerous agencies joined to make it a three-day exercise. Participating agencies included SBFD, SBPD SWAT, American Medical Response (AMR), San Bernardino County Fire Protection District (SBCoFD), San Bernardino County Probation Office, the Office of Emergency Services (OES), and three hospitals. Probation officers participated because the Probation Office assigns an officer on a rotating basis to each high school.

The purpose of the training was to test unified command and patient transport/triage. It was quickly determined that physically carrying patients long distances was out of the question as it was exhausting. They tried different modes of moving patients and found the use of vehicles was the only reasonable and practical solution to move patients from the scene to triage in the warm zone.

Day 1: Triage was too far away from the site and transfer of patients was difficult. They considered using a nearby middle school.

Day 2: Triage was moved closer. (The CCP must be nearby because carrying casualties by hand wore everyone out.)

Day 3: Triage moved onto the school grounds. They simply drove up to the school and parked on the grounds.

The participation of probation officers led to the familiarity with SBPD and working together to move victims. From this training, the SBCUSD Emergency Manager understood the importance for unified command to have PD and FD commanders physically with each other.

Several lessons came from this training. Participants learned that the system may not have a solution to the problem, a belief supporting improvisation (Weick 1993). They also gained experience in collaborative problem solving across disciplines.

Their plans were not improvised. Improvisation *was* the plan. Training can support this approach.

FIRE/EMS EXPOSURE TO POSSIBLE IEDS

Every response to an active shooter must involve a strategy for identifying and eliminating the presence of improvised explosive devices (IED). There will always be a degree of risk of an IED in the crime scene as well as secondary devices specifically targeting first responders in likely staging and response areas. European and Israeli first responders are well-versed in this tactic and experienced in searching out possible IEDs early in an event. US first responders may be forced to develop these skills. This goes back to scene assessment that all first responders learn.

This is particularly important when considering complex terrorists attacks, specifically attacks against First Responders that have occurred in Afghanistan and Iraq, where the initial event is planned to draw in a response, so that terrorists/insurgents/attackers are able to concentrate more targets (ie First Responders) in an area but utilizing the First Responders SOPs and TTPs against them.

BEYOND PROCEDURAL LEARNING

Smaller training sessions can make skills almost automatic and larger sessions create instinctive reaction training. In these sessions, participants learn there are no individual actions. All actions interconnect and are influenced by the immediate environment. But, this only works when participants actively pay attention to each other, responding to those around the individual (Weick and Roberts 1993).

Responsiveness includes visual cues and the tone of voice, just as much as words. This form of communication is vital in a noisy or crowded environment. People may not hear though they are adjacent to each other or two may be in line of sight, not knowing who is being spoken to. Visual cues can confirm the message or be the message. Observers, regardless of the distance from this action, are likely to miss it or misunderstand its relevance or significance.

Reviewing records and interviews, the rapid yet smooth integration of diverse teams and disciplines came together in this incident much like that of launch and recovery operations on the flight deck of a US Navy Aircraft Carrier. For launch and recovery, different job tasks and responsibilities of diverse teams synchronize to achieve the mission of the safe launch and recovery of aircraft. Public safety personnel, throughout this incident and with little effort, self-organized to achieve effective resolution of this incident and just as rapidly improvised solutions for emergent problems. “Little effort” is a key concept because this method of organizing becomes the routine, if not natural, way of working together. It appears much of the information the participants in this active shooter incident needed and used was present in the context of the incident. We can characterize this effectiveness in operations as "self-organizing improvisation” and a method learned in training sessions and routine operations.

The great value of this type of training lies in the opportunity for collaborative problem solving, improvisation, and familiarity with the way other agencies operate.

EXPERT SKILL PERFORMANCE

The control operator form of HRO rightfully adheres to processes to manage dangerous technology. In the VUCA-T environment, however, this suppresses development of expert skill performance. Knowledge-based systems rely on intuition, knowledge, and experience for effective decision making (Rasmussen 1983; Dreyfus 1997 and 2004). When the expert performer must follow rules, the performance level reduces to the level of rule-based performance, that of competence.

Competency-based systems work well with high-turnover employees because training becomes straightforward with a rule-based system and no discussion of particular situations (Benner 1982). The fire service relies on knowledge-based members who remain for years, if not decades. This gives time to learn particular cases and prepare for unexpected events.

Competency and proficiency build from lists, rules, protocols, and standard operating procedures (SOP). The attraction of these methods is their standardization and application free of context. They also assume the existence of a right answer. Duncan Dieterly (1980) described as trivial the problem with a known situation and known objective, needing only a single or a few interventions. Knowing, or collecting, sufficient information to solve the problem reduces problem solving to puzzle-solving (Wolfberg 2006).

Public safety personnel, in order to engage the VUCA-T environment, move past the trivial problem and puzzle to Dieterly's (1980) undefined problem where we do not know the situation, or the problem yet must find the solution. A more productive approach is to mystery-solve (Wolfberg 2006). In other words, we are not sure of the problem, the information, or the solution. We figure it out as we go. This describes the expert performance described by SBFD and allied agencies.

Operations in a dynamic, fluctuating, time-compressed incident reveal the problems and contradictions from application of discrete concepts to continuous perceptions of the incident (Weick 2011). Actions in the VUCA-T environment are simultaneous, interactive, responsive, and anticipatory to take advantage of a full spectrum of possible actions (Wolfberg 2006). Full-spectrum analysis (Wolfberg 2006), essential when the environment contains relevant information, takes consideration of every type of information, beyond what is considered necessary or sufficient in medical models or public safety practice. As this incident demonstrates, moving to full-spectrum analysis is rapid and reliable.

In the VUCA-T environment, decisions are ineffective when taken as discrete actions such as selecting a protocol or as a series of decisions following an algorithm. Decisions as discrete entities do not allow for incorporation of changing perceptions or changing situations nor the interaction between perception and changing situation (Weick 2011). To enter the VUCA-T environment, the field operator must balance concepts, perceptions, responses, and changing threats.

That is a problem to identify lessons learned after an incident that can be used for training. It is the nature of performance in the VUCA-T situation that makes it difficult to remember, recall, or retrospectively analyze events.

For problem-solving in a novel or hazardous situation, the expert relies on short feedback loop decision making (e.g., OODA cycle), orienting from the organization's culture, and use of practice-based behavior. The expert does not use rule-based protocols and practices, which ensure success in stable environments but do not adapt to particular circumstances or VUCA-T environments. The decision points and actions of the response exist along a continuous spectrum. They fall somewhere between the linear theoretical rules and algorithms developed in the calm and safe

offices of planners and trainers, and the VUCA-T environment that more closely aligns with real-world mystery solving.

LEADERSHIP

I didn't have any second thoughts about the fire department's response. There was no "I wish we would have done 'X' in this event."

SBFD Battalion Chief

INTRODUCTION

Leadership in public safety must account for 1) operations in dangerous contexts, 2) serving outgroups associated with attacks on public safety personnel, 3) the meaning of providing public safety in situations with grievous loss, and 4) reappraisal after a critical incident to give meaning to the experience. An individual who directly threatens a member of a public safety organization will not only affect the member's attitude toward that group but may create permanent memories that influence later behavior. This is discussed elsewhere in this AAR, but we evaluate in this section the part leaders have in generalization of these attitudes to all members of the outgroup. For example, a wealthy individual, under the influence of illegal drugs, may threaten immediate harm to the member. This can change attitudes, and, for personal safety, the member may self-categorize (mindlessness) and be more guarded at the next encounter. The member of the public, sensing this, develops a negative attitude toward public safety that can then endanger members who later work with this outgroup.

Leaders in public safety have a positive effect on maintaining idealism in the motivation of subordinates by emphasizing the significance and idealistic aspects of public safety service. The leader can also educate subordinates about the socioeconomic characteristics of the outgroup in an effort to avoid stereotyping groups of people.

The significance of this leadership becomes apparent both before and after a critical incident as subordinates are creating their appraisals of groups before an incident and then afterwards as the members reappraise the incident and any part an individual from an outgroup played in the emergency. Negative reactions to groups created by a critical incident can become deep-seated and contribute toward creation of biases and prejudices.

Leadership models developed in organizational science or military organizations may not generalize "live-or-die" situations where one must actively avoid death (Kolditz 2006). In such situations, leaders and followers personally face dynamic and unpredictable situations where outcomes include severe physical or psychological injury (Campbell et al. 2010). This type of leadership requires context-dependent factors and development of affective thought processes (Palmer et al. 2011; van Stralen et al. 2017).

Full-spectrum leadership, necessary for these extreme incidents, begins with the rookie's enculturation of the organization's attitudes, values, judgment, and beliefs. While it is well accepted that leaders prepare their unit for high-risk operations it appears less well understood that leadership after the critical incident helps maintain members in operational readiness, reduces some of the negative effects of a critical incident, and can convert what could have been a scarring experience into strengths.

From her experience as a clinical psychologist who served four tours in combat zones from the Persian Gulf to Iraq and Afghanistan, and who participated in the debriefing after the Navy Yard Shooting, Kathy Platoni, Psy.D., DAIPM, FAIS, COL (RET), US Army, has identified the importance of leadership after a critical incident (personal

communication). “How officers are treated by leadership following traumatic events determines how well officers recover from trauma. Most officers will remember, long past any traumatic event, the conduct of every member of their chain of command and who called, who cared, and who showed up.”

After the critical incident, some leader behaviors, from Dr. Platoni’s observations, can cause harm. “Being let down by leadership, abandoned, and mistreated when most vulnerable is [unfortunately too] commonplace and completely unacceptable in any case. Abusive, incompetent, inept leadership results in an enormous loss of productivity, costing billions every year.” These leaders will, intentionally or unintentionally, drive out highly qualified operators.

“Good leaders who support the troops and demonstrate compassion, caring, honesty, fairness, integrity and professionalism, and a listening ear are a rare breed.”

We, in public safety, must recognize and acknowledge these leaders.

Without supportive leadership after a critical incident, Dr. Platoni has further observed that the operator must then assume responsibility for finding constructive ways to cope. She recommends the operator find someone objective within the department, particularly on the same shift, to talk with to receive equally objective feedback and have the chance to ventilate. Important in all post-incident reappraisal is validation of how the operator made sense of events and the actions taken by the operator. To reduce “mental scar formation” we advise the isolated operator to seek mental health services to find even better ways to cope, function, and to explore other options. “Think survivor and solution, not victim.”

LEADERSHIP IN THE FIRE SERVICE

Without leadership, sensemaking can collapse with deadly consequences (Weick 1993). To counteract vulnerability in dynamic threat, the leader maintains respectful interaction to synthesize meanings when two or more people have a different sense about the same situation (Weick 1993). Leadership skills in life-threatening situations are unique, tied into the ideals of optimism, hope and resilience (Kolditz and Brazil 2005), the values of trust and honesty, and the beliefs of self-respect in moment-to-moment interactions (Weick 1993).

When the leader frames the situation as uncertain and dynamic then subordinates will increase their vigilance toward outliers and disruptions (Barton et al. 2015). The authors found higher performance in wildland firefighting teams when they were engaged with the context of the situation and proactively sought outliers.

The leader-leader culture is fostered rather than the leader-follower approach because role systems may not be able to change fast enough within these chaotic and threatening environments. Any member of the team can transition into any role at any time, fostering a mindset for vigilance to threats, a sense of responsibility for self and the team’s safety and welfare, and acknowledgment of command. HRO culture and a leader-leader structure facilitates coordination between individuals. This, literally, forms the group as each person can now reconstitute the group and assume whatever role is vacated (Weick 1993).

After analyzing firefighter near-miss records, Benjamin Baran and Cliff Scott (2010) identified eight leadership categories. The culture of firefighting “organizes ambiguity” for the dangerous contexts of firefighting. These categories are situational awareness, direction setting, communication, knowledge, role acting, agility, role

modeling, and trust. The authors also identified several higher-order categories: *framing*, *heedful interrelating*, and *adjusting*, described below.

Framing involves effectively gauging the level of risk present in the environment. Firefighting teams place the ambiguity of their situations into an initial context, or frame, as a means of focusing their attention, permitting them to receive information about what is important and what is not important about the event. Leaders must draw on lessons learned from prior equivocal experiences—past successes and failures—to contextualize the type of situation they have encountered. Not only do they use their collective experience for framing, but framing is also influenced by organizational structures such as policies, procedures, and formal reporting relationships.

Heedful interrelating describes how a group comes to a *joint conclusion* about what is plausible in the environment. This is an interactive process of collective sensemaking, actively sensitive to available and changing indicators to understand the situation with the serious realization that each change in evaluation also changes the rules. They always question assumptions, including the communications they receive. They do not want to assume that they have heard a communication correctly. “Freelancing” behavior, that is, disregarding Commander’s Intent, hinders heedful interrelating by being heedless of the situation risking serious risk harm to others.

Adjusting describes a flexible posture toward change that firefighters continually maintain. They not only maintain awareness of their surroundings but also continually revise and update their conclusions as a form of collective mindfulness.

In the more dynamic and dangerous incident, leadership must match cognitive processes and affective processes in a complex and growing incident. This places cognitive demands on the leader to have a mental model of the system that exceeds the complexity of the evolving incident (Ashby 1958). “Complexity must match complexity," describes the need for the requisite variety of resources a leader must be able to call upon.

Koldtiz (2010) describes these types of organizations that enter the VUCA-T environment as normative, meaning its members are values-based, performing out of a sense of duty and have the ideals of optimism, hope, and resilience. Their leaders have the opportunity to model leadership ideals, receive specific leadership training, and have experiences in dangerous situations.

It is important to distinguish the use of “duty” in public safety cultures, which is duty to others and the community, from “duty” as a task that must be performed because of a person’s job. It is the sense of duty to others in the fire department and in public safety that drives self-organizing and the sense of duty to the community that drives the level of service provided.

LEADERSHIP *IN EXTREMIS*

From their experience in combat, Deirdre Dixon et al (2016) identified five key sensemaking activities that enable effective leadership: synchronicity, hyper-focus, reciprocity, sense of duty, and instinctive reaction training. We will describe these five and use them to interpret leadership during the active shooter incident. The similarity of the work of Noel Palmer at al (2011) with law enforcement allows us to incorporate both models into one.

Synchronicity: Sensemaking and sensegiving occur simultaneously in a continuous cycle as information about the situation accumulates and is processed. Karl Weick (1995) identified these “retrospective” and “ongoing” characteristics of sensemaking as the reason events seem like they no longer act in a single sequence. The individual

constantly looks back to see what is still working and what has just failed contributing to improvisation. This shift in time perception is also the difference in time perception between low context and high context cultures (Hall 1959). Low context cultures, such as North American culture, generally view time as an arrow traveling in a straight line and prefer to work in sequence to "get things done." In public safety events, time is overlapping, we do many things at once, and we think of what to achieve as we work to achieve it. The difference is also clearly seen in the use of protocols between rigorous adherence in low-context systems and more fluid guideline in high context systems.

We create sense out of our situation through action. That is, we construct meaningful explanations for our situations from our experience with that situation. We must update our knowledge from the unfolding situation or risk being caught up in dangerous events if we still act upon what happened in the recent past (Weick, 1993). Successful leaders process environmental cues while also conveying critical information to subordinates (Barton et al. 2015).

Hyper-focus: The urgency of sensemaking and sensegiving intensifies in a life-threatening situation. Though threat impairs our cognitive abilities, with conditioning an individual modulates the effects of the fear response to better interact within the threat environment. This is accomplished through affective processes in the brain. "Affect" describes attitudes, values, and how we experience and regulate feelings or emotions. Cognition describes the mental processes we use to acquire knowledge and to understand what is around us.

Leader resiliency is a significant element of leadership *in extremis* (Palmer et al. 2011). Resiliency is to be reactive to setbacks rather than focus on future expectations or rely on optimism. In dangerous contexts, we experience cycles of success and failure. Linking identity to successes also sets the individual up to link to failures. The linkage to success or failure depends on the degree and length of time, both out of the leader's control but observable in the morale of subordinates. The leader guides subordinates away from identifying strongly with success, reframing all results as merely information to be used to guide the decision-making to influence the trajectory of events and achieve the desired end-state.

Reciprocity: Sensemaking is actively shared by all who generate information. The leader is receptive to new, especially disconfirming, information. For example, subordinates may notice the leader lost focus on events and, through reciprocal sensemaking and sensegiving, might intervene to interpret a situation, suggest an appropriate action, or encourage communication with other leaders. This creates distributed cognition as every team member understands the mission and knows what others will do during the incident.

Sense of duty: Even though hyper-focused in "*in extremis*" situations, the leader maintains self-awareness and modulates feelings of fear and the threat reflexes. The leader makes decisions toward their shared sense of duty. All members of the organization share a sense of duty to others. Sense of duty in the fire service is reflected in the willingness to help others, contribute to the successful solution of the situation, and to sacrifice for the good of others.

The embodiment of specific values and beliefs is central to the self-concept of what it is to be a firefighter. Embodiment is alignment of behavior and espoused values; one acts as role model. This is the highly developed moral capacity described by Palmer et al. (2011).

Instinctive reaction training: Extensive training often creates automatic behaviors, increasing the leader's cognitive capacity for problem solving. Training, however, in the VUCA-T context refers to behavioral routines as actors cannot rely on rules, protocols, or algorithms to deliver a successful solution.

The leader cannot rely upon reaction training as a substitute for cognitive capacity. Reflective and informed experience builds a repertoire of adaptive knowledge useful for a variety of experiences. During turbulent events, knowledgeable adaptation is essential.

LEADERSHIP IN ACTION

SYNCHRONICITY

By acting we make sense out of the incident. *We act in order to think.*

On December 2, 2015, there was no distinct, advance plan to rely on probation officers to select patients and establish the casualty collection point. Nor to rely on probation officers to drive casualties to a triage area.

- As the leaders involved interacted with the scene, they felt they had met a threshold of safety that would allow them to begin extracting casualties.
- The probation officers demonstrated the interest, ability, and drive to rapidly learn they could move the casualties.
- Fire/EMS personnel in the crime scene directed the probation officers away from the deceased, focusing them toward the remaining salvageable patients.
- Officers began appearing with vehicles and the leaders assigned them to moving casualties to the treatment site.

The arriving battalion chief quickly realized he was the fire department incident commander (FD IC) and took command.

- He sought the location of the police incident commander, but all he could see was a sea of police units parked haphazardly on Waterman Avenue.
- The San Bernardino Unified School District (SBCUSD) Safety/Emergency Management Manager was on-scene with his emergency vehicle with complete radio compatibility between fire and police radio frequencies.
- The SBCUSD police officer assisted the Fire IC in making contact with the Police IC who was conducting police operations from the trunk of his patrol car.

AR (Air Rescue) 006 orbited over the incident scene.

- They could land, exposing the helicopter to damage from the assailants or delaying a response to a secondary incident.
- They could continue to orbit but would not be able to provide assistance to the casualties in the conference room.
- They landed, Sheriff's Air Rescue paramedics assisted the SWAT Medic, and AR 006 transported two patients, immediate and delayed.

HYPER-FOCUS

Remain focused and regulate emotion.

Public safety personnel learn quickly that rarely do the details of the dispatch match the situation and conditions on the ground.

The fire captains who responded to staging maintained their positions.

- They perceived the urgency of responding to the patients.
- They observed a law enforcement officer waving them in.
- They remained staged, partly because of discipline but also from their ability to remain focused on their mission and regulate their emotions that might have otherwise driven their response.

The Fire IC preassigned fire companies for treatment, triage while in staging.

- The Fire Captain assigned a paramedic to be the Med Comms (Medical Communications Officer) who then immediately notified Med Comms while they were still in staging.
- Fire companies arrived at Triage A and, without words, immediately pulled gear and set up Triage A; patients arrived just as they completed set up.

The gravity of this response was well recognized from various clues.

- SBFD personnel heard the number of fire companies responding, the number of police responding, and the speed of the police vehicles as they passed by to the scene.
- SBFD personnel monitored police radio frequencies.
- They quickly realized this incident may involve many more casualties than they had seen before.
- SBFD personnel regulated their emotions and scaled their expectations and preparations to meet the anticipated numbers.

RECIPROCITY

Actively sharing information.

The overall impression of fire personnel was that the Fire Chief was “everywhere.”

- Asking questions and offering suggestions and assistance.
- Asking one BC, “Do you want me to take over [one command] and you take [another command]?”
- The Chief hung around, listening in, assisting with command as needed.

The SWAT Medic requested a Captain and his crew to come with him into to IRC building.

- The SWAT team was actively clearing the multi-story building.
- The Captain personally knew the SWAT medic, trusted him with the security of his engine crew.
- The firefighters approached the conference room entrance with the armed SWAT Medic providing security.
- (Communication and reciprocity occurs visually and through tone of voice.)

INSTINCTIVE REACTION TRAINING

Train to automatic behavior.

The casualties arrived at Triage A.

- Personnel responded to their individual MCI duties with a practiced familiarity derived from many experiences with groups of wounded or injured victims.
- Everything was working as it was supposed to. The 3T (triage, treatment, and transport) response seemed like it was a program that was simply unfolding.
- The critically wounded entered the process, those whose need was most critical were transported immediately.
- It felt like the 3T commander was just the maestro who was coordinating the response.
- The 3T leaders were doing the work and kept him informed with little input from him.

Fire was then immediately cleared from staging to Triage A.

- They parked and rapidly pulled out salvage tarps.
- A police officer paired up with the captain and said, “We’re going to be bringing patients out soon.”
- One patient was brought out and quickly transported.
- Probation Officers with pickup trucks were bringing patients to them before the 3T team was able to fully set up.
- The first wave of patients arrived in a pickup truck and one in a car.
- Two deceased individuals were delivered in the first wave of those transported.
- “I thought we would be tied up a lot longer treating patients, but they were gone in what seemed to be a blink of an eye.”
- There were waves of fire/EMS personnel doing one task multiple times for multiple patients.

SENSE OF DUTY

Willingness to help others.

Numerous fire companies self-dispatched.

- Reduced response time, resulting in help getting there sooner.
- Some fire companies arrived at staging early and had the discipline to remain in staging despite their perceived urgency of the patients they knew were there.
- One company did not stage but drove directly to the treatment site.
    - They began setting up and realized they had no triage tarps.
    - They immediately improvise, using salvage covers and marked “Immediate,” “Delayed,” and “Minor” on the street with chalk. Patients had not arrived yet.

PD and FD commands were not immediately, physically connected.

- A hybrid command effectively grew into an efficient CP.
- SBCUSD police officer connected FD command with PD command

The FD IC pre-designated assignments to fire companies.

- Each captain and the crew used this opportunity to perform a mental checklist of their tasks.
- A Battalion Commander remarked, “What I did know, the people on-duty were going to perform to the best of their ability.”

When personnel within an incident take action, the commanders want to know.

- The fire crew entered the warm zone without command authorization.
- If it is not an authorized action yet within the individual’s or team’s purview, the commanders continue to support them.
- This flexibility of response without command knowledge or permission demonstrates the freedom to react to the local truth within the team’s mission limitations.

TRIAGE AND SURVIVABILITY

Emergency medical care is provided by the fire department, but certain aspects are regulated by the Inland Counties Emergency Medical Agency (ICEMA). Care *not* requiring delegation of medical authority includes manually acquiring and protecting the airway, bag-valve-mask ventilation, and hemorrhage control. Airway intubation, tourniquet placement, thoracentesis, and intravenous therapy are regulated by ICEMA. Determination of death is also regulated by ICEMA.

An active shooter incident combines the system demands from a multiple casualty incident (MCI) with the local Public Safety demands of a hostile environment. This is most clearly observed when a moribund patient is either in a hazardous location accessible only by a law enforcement officer or the patient is in a group of equally critical patients. It is nearly impossible to judge, at any distance, the order for who to treat and transport in a hazardous scene. Death will come to some patients, not because of the actions or inactions of the fire department or EMS, but because of the circumstances created by the active shooter.

Methods for patient evaluation were developed in a well-controlled environment without the degree of time compression and threat experienced in a hazardous environment. Care must be taken not to interfere with the rapid, fluid patient evaluation and management necessary in the active shooter incident. In all of our interviews, the evaluation of the dead was consistent with following modest modifications of START Triage, mainly deletion of a numeric value for heart rate and respiratory rate.

Triage scoring has the same difficulties as many of the conceptual differences between an HRO and conventional organizing. The patient has a nonlinear, full, continuous spectrum of diseases while trauma scoring systems are linear and segmented. Environment is an essential consideration.

The medical literature and business models rely on cognitive processes. Discussed in other parts of this AAR is the danger this poses if we disregard the affective processes. A triage score that is delayed in a healthy patient in a safe environment may also create the “delay” level for an unhealthy patient in an unstable environment who would more accurately be an "immediate" level of care due to circumstances.

TARPS (SALVAGE TARPS)

For a multiple casualty incident (MCI), the truck companies normally carry kits that contain colored Triage Tarps. The kits had recently been removed to evaluate them in preparation for an MCI drill. The truck companies used salvage tarps and marked the area with chalk designating the particular salvage tarp as "immediate," "delayed," "minor," and "deceased."

There was an initial announcement for what the three tarps were for and it may not have been repeated. The absence of colored trio tarps did not hamper care. Paramedics could tell visually where the patient should be placed. In fact, for the immediate tarp, more serious patients were spontaneously placed adjacent to the side for ambulance pickup.

TRIAGE TAGS

Triage tags do not appear to assist in identifying level of triage, despite being called "triage tag." Rather, they may have acquired the name because they label the triage level of a patient. This is significant because all medics interviewed did not see how the tag helped them triage. The tags did interfere with their "3T work" of triage, treat, and transport.

In this active shooter incident, one patient transported by ambulance did not have a tag and there is no record a tag was placed at the scene.

The fire engineer brought triage tags with him when the fire company went to the conference room in the rear of the IRC and placed tags on the patients and deceased. Some of the tags may have become water damaged but there is no record of a tag being lost due to water damage.

Triage tags are not typically carried by SWAT Medics in their first-line med gear/bags. SWAT Medics may have them available in a back-up bag which is usually left in a SWAT vehicle. A SWAT Medic's "In and Out" bag carries very specific equipment that can bridge the gap between point of wounding and rapid casualty evacuation to a hand-off crew or medical facility. Since the SWAT Medic's primary role is to support the members of the tactical team, the capabilities in a MARCH belt or Aid bag are focused toward potential or possible injuries specific to team members. For any mission type, the medic must always evaluate mission-specific risk to decide what to pack, remembering that speed is security for the SWAT Medic.

A steady flow of patients arrived at Triage A, driven by San Bernardino Police Department SWAT's armored vehicle (Bearcat) and via VOOs (including a Ford Crown Victoria and Probation Office pickup trucks). A medic placed a triage tag on each arriving patient which, all agreed, interfered with efforts to treat patients, particularly when ambulances started stacking up. They stopped halfway through and, instead, placed the tags on the patient at the ambulance.

One medic used triage tags as a formality because that's how they were trained. The tag had no bearing on outcome on scene and was soon seen as a waste of time. All medics felt the triage tag system and START system of triage were ineffective. Colored tape, as quick patient marking system, was suggested.

The hospitals did, however, report the usefulness of the tags. There are no reports if the hospital used the tags for tracking patients, to determine medical care, or to determine destination within the hospital.

If tags are to be used for patient tracking from scene through hospital admission, then calling them “tracking” tags would be more descriptive and may increase compliance with tag use. If they are to be used to determine treatment in the hospital, then space to record a few vital signs and exam information should be added.

PURPOSE OF TRIAGE

Triage can be a score, a threshold limit of vital signs, anatomic injury pattern, abbreviated history or a brief physical examination. Triage is likely to be some combination of these elements. Without distinguishing the purpose of triage, the elements may not properly contribute to the triage decision and make the triage evaluation lengthy and involved.

The purposes of triage seem to overlap, ranging from decision to immediately treat, priority to transport, decision of mode of transport, location and type of receiving hospital, to reduce hospital surge, to predict emergency department treatments, predict intensive care unit or hospital length of stay, or predict probability of death.

In line with purpose are environmental constraints and ease of obtaining the necessary information. As one medic pointed out, he is expected to use respiratory rate and is also expected to accurately measure respiratory rates for 15 seconds. This quickly adds up to minutes and occupies a paramedic with counting breaths rather than treating patients. If this is unreasonable then the medical authorities of the system should determine if the 15 second count is necessary.

Once the determination of purpose is made a score can be identified for that purpose and a qualified person identified to teach medics. Medics can learn all of the elements in a triage score and can learn excellent judgment.

In some regions triage may: 1) support helicopter use, 2) prevent a patient from admission to a distant hospital, and 3) reduce patient surge.

Triage tools are independent of context in that they do not account for higher patient numbers, greater severity of injury, or limited on-scene resources. A score for this setting would be rapid, serve the needs of the paramedic and patient, and tend toward over-triage. Many medics reported concern for patients who had gunshot wounds to the trunk yet had no visible blood or had multiple gunshot wounds to extremities and in the trunk.

MEDIC TRIAGE

“There was triage to which tarp, triage to treatment, and triage for transportation,” said one Triage Medic. The medic triaged the patient to a specific tarp as the patient arrived from the casualty collection point. Once on the tarp, another paramedic triaged again for who is treated first. Then the transport medic triages again for who is transported, to where, and by what mode. The transport manager kept a mental calculation of patient destination (trauma center or hospital), reciting it in the interview 18 months later.

Every decision point had anatomic or physiological meaning; triage is not intuition or a “gut” feeling. Many of the measures could be noted simultaneously; triage is not linear. The weight placed on a measure varied depending on other elements and whether it could be changed; triage is not independent of context. For example, major bleeding is immediate until a tourniquet stops the bleeding. Respiratory distress is immediate until needle decompression relieves the tension pneumothorax.

They looked for trunk versus extremity, activity level, then skin and breathing.

Physiologic criteria

- Activity, all were quiet, there was not much talking.
- Skin is pale.
- The biggest clue is the skin is pale "because even black people have pale skin in shock or death."
- Breathing: "Immediate" patients were breathing rapidly but gasping, which was a more significant sign than rate. Respiratory rates above 30 can happen when people are frightened. Respiratory dysfunction can be present with rates under 30. Bilateral needle decompression can rule out possible pneumothorax or hemothorax.
- Bleeding is visible and was usually in the legs.

Anatomic criteria

- The trunk (chest and abdomen and pelvis) are all considered serious and "immediate" triage.
- If shot in the chest the patient went out before the extremity GSW did.
  - The shoulder joint is considered extremity, but it could be a chest wound, it would need evaluation for evidence the chest cavity was penetrated.
- Extremity, the bleeding can be controlled. The chest is more likely to expire.
  - A tourniquet for an extremity and it became less of an immediate concern.
  - All extremity wounds were less serious, the shoulder less serious than the trunk.

TRANSPORT TRIAGE

"Trauma center determination- trunk versus limb"

Transport Medic

For rapid decision-making, the transport manager sent any wound to the trunk or head to a trauma center. Any extremity wound was sent to a non-trauma center. The medics for transportation used the same criteria for extremity, with special consideration for the shoulder as described above.

The transport manager utilized an unexpected pause of 3-5 minutes between ambulance groupings to employ the Sheriff's Air Rescue helicopter. One reason was to take advantage of the pause to move patients and the other was to spread some of the trauma load out of the county. In a combined decision between AMR, Sheriff's Air Rescue medics, and SBFD they rapidly moved two patients to the nearby helicopter.

The receiving hospital in the neighboring county did not receive notification of the helicopter's destination. During the eight-minute fight time the medics administered oxygen, started IVs, and administered a normal saline bolus of fluid resulting in a decrease in heart rate. Radio traffic interfered with timely notification of their dispatcher and they did not have the radio frequency for the receiving trauma center for direct contact.

ICEMA in San Bernardino County later discussed transferring trauma patients out of county in circumstances when the county has the capacity to keep the patients. This is due to California law and not personal beliefs.

One of the patients received endotracheal intubation in the emergency department and a blood transfusion and the other was taken to the operating room for abdominal injuries due to GSW.

THE DYNAMIC NATURE OF TRIAGE

The medics found it was difficult to move a patient to a more serious triage level. The conditions of patients would change, either improve with treatment or deteriorate to reveal more serious injury. One medic re-evaluated a patient who developed back pain and found two bullet holes without blood and no visible hole in the shirt.

One medic was concerned that he was taught to never delay transport but when he took the time to treat a patient with pneumothorax, the patient improved. In that case, it appeared delaying transport in order to treat could make an unstable patient stable for transport.

TREATMENT

What the paramedic can ask is, "What is killing this person next?" Nothing a medic does in the field is a definitive treatment, these are only temporizing measures in order to get the patient(s) to a higher level of care for definitive interventions. However, there are interventions that can delay the lethal slide of compensated shock to decompensation, allowing more time for the patient to receive the proper level of care.

In these settings, application of the fundamentals of physiology and response to therapy can effectively guide actions, identify "immediate patients", and give more accurate descriptions. The purpose of the medic is to identify and correct derangements in physiology. This can be accomplished by using response to therapy as the guide. Did the patient improve, deteriorate or remain unchanged? *Unexpected* deterioration indicates an unrecognized problem and warrants further examination. No change is more difficult to interpret. If, after administering a bolus of crystalloid fluid, there is no change in vital signs or physical examination then it may be due to the wrong diagnosis or insufficient crystalloid bolus.

Several patients received a fluid bolus without significant change in physical examination or vital signs. Yet one patient received one unit of packed red blood cells in the emergency department.

Ultimately, in these cases the only definitive evaluation and treatment for these injury patterns is operative management with the most reliable assessment tools being ultrasound FAST-1 A examination (focused assessment sonography in trauma) and X-ray (American College of Surgeons Committee on Trauma 2012).

**Treatments by SBFD or AMR paramedics**	
Supplemental oxygen	5
Intravenous catheter	12
Fluid bolus (300 or 500 mL)	4
Needle decompression	2
Full spinal precautions	1
Tourniquets	0

START TRIAGE

Initially the medics stated they used START or RPM Triage. On further questioning, they stated they did not use either system. RPM triage utilizes measures of respiratory rate, pulse, and mental status. START utilizes visual (respirations and walking wounded), tactile (respirations and pulse), verbal (walking wounded and follows commands). Their earlier characterization reflected system expectations to use one of the two triage scores. The medics stated that, in a mass casualty environment, use of the START may slow triage and not properly triage patients. This is especially true for the "walking wounded" who may be compensating or are able to move even though injured in "the box," that is, the trunk.

In the opinion of several experienced medics, START classified patients as "delayed" when they felt the patient was "surgical expectant," that is, they would likely need urgent surgical care.

Several patients classified "immediate" or "delayed" did deteriorate or received unexpected major surgical interventions on arrival to the emergency department (see results section).

SURVIVABILITY: THE ENVIRONMENT AS PATHOLOGY

The "Golden Hour" has influenced EMS systems to support expeditious transportation of trauma patients from the scene to a trauma center. It has also motivated some physicians and surgeons to respond into the prehospital environment to initiate care. The active shooter incident brings attention to the contribution of time and environment to death independent of wound characteristics. That is, a wound survivable if transported to a trauma center for operative repair within 30 minutes will have a different survivability if the victim is in a room with a barricaded active shooter. The idea of time contributing to increased mortality led to the concept of the Golden Hour. Delays in care are part of every rural community or our own county where a motor vehicle collision in the desert stretches of interstate or state highway deducts significant time from the Golden Hour.

Environment as a contribution to mortality, independent of the injury, is also not foreign to those who live in harsh climates. The active shooter, or the threat an active shooter may remain in the area, creates an environment restricting medical assets from reaching the patient.

The idea, then, that a VUCA-T environment contributes to death is not irrelevant to discussions of mortality. Patients may have a readily treatable injury, but a paramedic working under the constraints of the EMS system may not be able to reach them because of extreme environmental conditions.

In this incident, some in the medical community have suggested, if not pronounced outright, that had the paramedics transported the deceased rather than make a determination of death, those victims had a chance for survival.

For this reason, we conducted an extensive review of the deceased, wound patterns, and the circumstances of death with trauma surgeons, intensive care physicians, civilian paramedics, and medics from the Special Operations Forces.

Healthcare professionals unfamiliar with the VUCA-T environment can readily interpret a wounding pattern as potentially survivable. This reflects a lack of appreciation of the environment and time as comorbidities public safety must "treat" in addition to physiological pathology.

Healthcare recognizes a patient may develop a complicating disease or complications as result of a secondary illness, the disease process, or medical and surgical treatment. For example, Adult Respiratory Distress Syndrome (ARDS) can develop in a patient with pneumonia despite an easily treated bacterial infection. Even after successful treatment of the inciting infection, death may follow the ARDS complication.

In a similar manner, a casualty may have a treatable wound in the context of an emergency department. In the context of an active shooter event, the environmental pathology may delay the safe interaction with that casualty, consuming time from the Golden Hour, and the narrow window for effective treatment could be lost. For example, a casualty hemorrhaging from a large artery in an extremity may respond brusquely to a compression bandage, tourniquet, or intravenous crystalloid solutions.

Delay causing death, however, may result from the presence of an active shooter, a nearby explosive device, and/or the over-riding safety need to clear the area or structure of threat, preventing the applications of those life-saving modalities. The term “potentially preventable death” can lead some authorities and the public to disregard the effect of environmental pathologies, such as an active shooter. Instead, they will attribute the primary cause of death to actions or inaction of first responders. A better appreciation of environmental threats from uncontrolled behavior and energy will help more people recognize the environment as a pathology independent of the shooting.

Earlier, we described time as an independent contributor to mortality, which can reduce survivability. The VUCA-T environment is a pathology (van Stralen et al. 2017) and acts in the same manner as a comorbidity does in medical care. A comorbidity is an additional medical condition or disease that is independent of other diseases or conditions. Time and environment, then, exist independently of the wounding but are interdependent with the wounding when evaluating survivability. Just as inflammatory processes or adverse drug reactions are a threat that can cause death in a patient with a treatable disease, in the VUCA-T environment, the threat from the environment can cause death even though injuries may be readily treatable in a clinical setting.

Nine of the deceased had immediately fatal wounds. Five of the deceased had nonsurvivable wounds, defined as severity of wound plus extended wounding time.

DEATH

After the active shooter incident, paramedics began to hear from some physicians that, if the medics had transported all the patients, then some could have been saved. This may result from a misunderstanding of the patient injuries, the length of time between wounding and encounter with EMS, or the environment at the time of triage. Even arriving 10 minutes later can change the nature and degree of threat.

Describing the environment as VUCA-T *at the time of rescue* may help others understand the nature of the work performed. The use of wounding time as part of the patient evaluation would describe to others the lapse of time between injury and EMS reaching the patient. Combined with trauma’s Golden Hour, a person can readily see that half of the Golden Hour expired if EMS cannot reach a victim in 30 minutes. This is not to say public safety will not rescue such victims, only that time becomes part of the calculation of risk of death to public safety personnel with likelihood of death of a victim.

The discussion that the first responders in some way contributed to the death of a victim was taken personally by all involved and it was not considered to be supportive of EMS, the fire department, paramedics, or the public safety services involved. There was no suggestion from those interviewed that the medical community stepped in to explain

the circumstances. One aim of this after-action report, and the reason for its breadth and depth, is to give the first responders the science and language to explain their work to the medical community.

Rather than discuss this as “death,” we may better be served by calling this futile therapy. In medical ethics, a physician is not obligated to offer futile therapy. We can also consider other terms such as "irreversible injury likely to be nonresponsive to current field treatment,” or "death expected despite treatment."

Using a color scheme such as black versus gray can become a euphemism to avoid describing the patient’s condition, which is "moribund.” This contributes to the larger discussion of other risks in the prehospital environment. For example, balancing the risk to paramedics and the patient for using a helicopter to transport a patient also applies to the environmental risk (VUCA-T) to paramedics in reaching a "moribund" patient.

DETERMINATION OF DEATH

Medics interviewed used the same visual method for determination of death, patient is not moving, gray, and not breathing. The medic would then feel for a carotid pulse in both arteries.

- No observable spontaneous movement.
- Observed ashen gray skin.
- Skin is gray, reliable with darker skin pigmentation.
- Check breathing visually.
- Check carotid pulse, one artery at a time.

In each case, several medics examined for death determination shortly after a medic made the determination. This occurred two to three times for each deceased.

Autopsy review by the AAR Team and trauma consultants found no survivable wounds.

One suggestion for the warm zone is to clearly mark obviously deceased victims. This would be in a situation where LE has no means of marking the bodies (tape, triage cards, etc.). Possibly a sign such as roll face down and cross legs and arms for identification to lessen the chance for redundant assessment and unneeded movement or evacuation to CCP.

ICEMA Reference 12010

Determination of Death Criteria (Obvious Death)

- Decomposition.
- Obvious signs of rigor mortis such as rigidity or stiffening of muscular tissues and joints in the body, which occurs any time after death and usually appears in the head, face and neck muscles first.
- Obvious signs of venous pooling in dependent body parts, lividity such as mottled bluish-tinged discoloration of the skin, often accompanied by cold extremities.
- Decapitation.
- Incineration of the torso and/or head.
- Massive crush injury.

- Penetrating injury with evisceration of the heart, and/or brain.
- Gross dismemberment of the trunk.

ICEMA Reference 15030

Determination of Death Criteria (Penetrating Trauma Arrest)

If the patient does not meet the *“Obvious Death Criteria”* in the ICEMA Reference #12010 - Determination of Death on Scene, contact the Trauma base hospital for determination of death on scene for those patients who suffer a traumatic cardiac arrest in the setting of penetrating trauma with documented asystole in at least two (2) leads, and no reported vital signs (palpable pulse and/or spontaneous respirations) during the EMS encounter with the patient.

No deceased victim had survivable wounds, defined as severity of wound plus extended wounding time. Neither did any deceased victim meet the criteria for determination of death by ICEMA protocol.

SUPPORT FOR PARAMEDICS

"I received a lot of negative feedback from the XXX ED nurses when I was completing my paperwork who thought that the two minor shooting victims should have been transported to a trauma center and not their facility. I advised them that in an MCI, this is exactly what should happen - the trauma or specialty hospitals should not be inundated with minor patients simply because of the type of trauma."

Ambulance medic

Triage and treatment make an already difficult incident even more difficult. Public safety has a culture of mutual support. In this incident, there were comments from multiple people that more lives could have been saved had the paramedics operated differently. We report here an incident where paramedics transported a patient with gunshot wounds, and nurses heavily criticized the medics for going to their facility rather than a trauma center. This demonstrates the difference in operations between the emergency responder form of HRO, to “get it right,” and the control operator form of HRO, “to do it right.” To achieve high reliability, the organization and system must have fluid changes between the two processes.

We place this in the triage section rather than critical incident stress management because the participants directly questioned the care provided by medics and the operating philosophy of the EMS system during a high-risk operation.

Issues:

- Triage scores could reflect the practice of experienced medics for a measure that identifies a patient who may deteriorate rapidly or would benefit from transportation to a trauma center.
- Triage Tags may better be called tracking tags to reflect their purpose and ensure proper use.
- Determination of death in a moribund victim or a non-salvageable situation should not put a medic in danger or interfere with the care of viable patients.
- Consider "no response" during a fluid challenge as a response, becoming an indicator to further evaluate the patient and repeat the trial.

WORKING WITH LAW ENFORCEMENT

Within their disciplines, the fire service and law enforcement have different cultural approaches toward their public safety responsibilities. The fire service uses teams, then teams of teams, to construct a perimeter around, and penetrate, a system formed by uncontrolled energy. Law enforcement pursues a thinking, maneuverable person, a threat who does not want to be caught or wants to hurt someone. For Law Enforcement, the more diffuse the members are then the greater the likelihood the suspect will be caught. This creates two distinct cultures but with some similarity and overlap.

This distinction in cultures is critical to appreciate the innate differences between the fire service and law enforcement. "Culture gives meaning about the word and guidance for accepted behaviors and beliefs within a defined group" (van Stralen et al. 2015, 425). Within public safety, a member from each service can stand side-by-side during an incident yet have different experiences and make sense of events differently.

Fire personnel organize around a top-down, structured team approach. Law enforcement, on the other hand, organizes with a bottom-up, organic approach. That is, a fire company engages as a team commanded by a captain and rapidly combining with other teams coordinated by a battalion chief. This top-down characteristic gives the incident commander the ability to strategically direct assets. The bottom-up characteristic of law enforcement gives the individual officer freedom to respond to the immediate environment. It is important to note that both public safety services actually operate simultaneously top-down and bottom-up. We describe it in this manner to demonstrate subtle operational differences.

The fire service structures its command system from the start partly because of the nature of the threat but also because their operations are larger and last for longer periods of time. Law enforcement is able to begin a structured approach only when their operations begin to stabilize, or sufficient personnel have arrived. We see this in the use of the Incident Command System (ICS).

PROFESSIONALISM

The professionalism exhibited by every public safety discipline in this incident is a mainstay of public safety culture. The ability of the two groups to work together is partly due to the frequency of gunshot wound calls. There is more to it, however, as probation officers do not routinely work with the fire service or treating injured people.

The different missions of the public safety disciplines merge in an active shooter incident. It is professionalism that drives one to support a different mission without performance degradation in their own mission. We see that throughout this call from the various fire services, law enforcement agencies, and American Medical Response and Symons Ambulance services.

FAMILIARITY

The primary services, SBFD, SBPD, and AMR, commonly work together on numerous shooting responses involving multiple gunshot victims. The procedure is for SBPD to clear the scene for SBFD and AMR to conduct their MCI protocols. The scope—and threat—of this terror attack was greater than anything any of these responders had experienced. Knowledge of their roles and understanding the necessary tasks allowed the various services to work together to reach efficacious conclusions for novel incidents.

VEHICLE PLACEMENT

Law enforcement rapidly places as many officers on the scene as possible, therefore they tend to park close to the scene. Fire personnel maintain ingress and egress in order to allow arrival of more or specialized equipment and to escape if the incident surges.

FORCE PROTECTION

Protection from an assailant uses a different skill set compared to protection from fire or hazardous materials. Working in an area of an active shooter, the force protection offered by law enforcement allows the fire service to focus on rescue and EMS. A successful response is the rapid mitigation of suspect threat, ending the wounding process, and the rapid shuttling of the wounded for processing through the MCI process, followed by the rapid transfer of patients to definitive medical care. Force protection duties are manifold. They include:

I. Fire/EMS staging.
II. Triage/treatment site.
III. Patient transport corridor from injury site to the CCP and/or triage/treatment site.
IV. CP security.
V. Scene perimeter.
VI. Evacuee security.
VII. Crime scene security.

CRITICAL DECISION

The change of law enforcement's definition of response to an AS incident from "Stop the shooter" to "Save life" may assist in changing police behavior in these events. These two responses have now created two missions for law enforcement post-initial call, the period when time becomes a comorbidity to the wounding. Through distributed judgment, on-scene officers will determine when to change their individual mission regarding what is most lethal to the victims: the proximity of the assailant or the distance from medical care. Time is not the only determinant, there is consideration of the number and location of officers and the area to be searched or secured. Nonlinear interactions between time, threat, and resources demand judgment from individuals and prevent rigid adherence to a specific protocol or algorithm.

The SWAT Commander recognized that he had enough LEOs to deal with the threat and re-tasked the remaining law enforcement officers to start extracting casualties. It speaks well to their professionalism that they redirected themselves from the suspect to the victims, knowing suspects could still be in the area.

This was one of the best, if not *the* best, call a SWAT Commander could have made.

POSSIBLE IEDS

IEDs present two distinct problems. One is the use of a primary explosion to bring first responders to the scene followed by a secondary explosion to harm as many people as possible. European and Israeli first responders are well-versed in this tactic and experienced in searching out possible IED early in an event. The second problem is the improvisation. The bomb may be designed to look like a common item.

There will always be a degree of risk of an IED in the crime scene as well as secondary devices specifically targeting first responders in likely staging and response areas.

In this active shooter incident, Redlands Police Department officers became involved in a shootout with the suspects. Nearly every available law enforcement responded. FS 231 was left unguarded.

One fire/EMS respondent said, “It’s something I think about all the time. We were totally exposed out there. There may have still been a few officers when radio reports of the (second) shooting and an officer down came out, but everyone (police) left…It’s just about coordinating but there were a lot of emotions going on, too.”

INCIDENT COMMAND SYSTEM (ICS)

THE PURPOSE OF ICS

Incident Command System (ICS) was developed to unify command, standardize communication, and identify communication and responsibility structure to assure integrated operations, unified strategy and common language. The ICS system has become the national standard of operations and is known as the National Incident Management System (NIMS) and is now a required mode of operation.

Interruptions of information flow regarding the evolving incident and actions taken will result in life threatening delays in transportation and treatment of casualties. Without knowledge of timely operations and current conditions affecting their operations, other emergency responders may be put at risk. Resource management may be lacking required equipment and manpower to meet the needs of the rapidly evolving event. Security of the general public, the incident scene, victims, and responders will be put at risk as the incident becomes further disconnected by independent actions and objectives.

The central problem is linking the various public agencies at an incident in that critical period at the beginning of the response. These agencies must link to have a strong response yet not become distracted by the efforts to link. Strong protocols of operation help each responding organization operate with a sense of order within its operation. However, they do little to connect the operational environment of Law, Fire, Emergency Medical systems (EMS), and Government. One of the initial efforts of public safety is health structure in an environment that unexpectedly lost structure.

This system, as part of Unity of Command, outlines a single command post, a single Incident Commander and a structure that includes all of the operating segments such as Law, Fire, EMS, and Government in tight communication and operations. The system solves many of the operational difficulties experienced in complicated, rapidly changing, high consequence incidents and is scalable to any size incident from everyday operations to large earthquakes and National Incidents such as hurricanes.

Emergency responders follow accepted processes and protocols during emergency operations. Upon commencing high tempo operations, responders must rapidly adapt to the situation toggling between standard operating procedures and the need for constrained improvisation to meet the emerging hazard. This mode of operation contributes to the rapid actions necessary to operationally meet the challenges of a rapidly developing incident. However, these rapid changes in operations, situation, and information can lead to a further disconnect between operational segments such as Law, Fire, EMS, and Government

ESTABLISHING ICS

Law enforcement (LE) established incident command (IC) early. San Bernardino City Fire Department fire command arrived minutes later. LE vehicles prevented the two commands from physically uniting in a unified command (UC). Radios without a shared frequency prevented them from uniting over the radio. The Emergency Manager for the San Bernardino City Unified School District found the San Bernardino City Fire Department (SBFD) fire IC and physically connected him with the LE IC. Two years prior to this incident, the school police officer attended a training scenario where the importance of having physical presence for the UC was demonstrated and led to his actions that day. He also had radio compatibility, allowing the FD IC to communicate with fire operations.

Though a "virtual" UC developed with the use of the SBCUSD radios, until a physical UC was established there were two commands, one for law enforcement and one for the fire department.

The FD IC then started to build up his portion of the ICS structure but was basically acting as medical branch and the LE IC functioned as the law branch. SBFD personnel were assigned for check in but LE officers did not seem accustomed to a check in system and being available from a known location. They arrived then seemed to stage themselves elsewhere. A helicopter had landed at the golf course and there was no air branch. One of the Air Rescue medics did walk over to the CP and offer to assist with air operations.

The intensity of the threat from the assailants, exacerbated by the number of dead and wounded readily visible, appeared to diminish the ability for LE to support SBFD. The number of incoming LE officers became not only a logistics problem with vehicles but with the number of officers openly carrying their weapons and in plainclothes. SBFD personnel shared their concern that other assailants may hide amongst the LE officers.

Further evidence of minimal ICS structure was the departure of LE officers from FS 231 and the Rescue Task Force (RTF).

THE LAW ENFORCEMENT RESPONSE

The IC structure early in the law enforcement response is decentralized with the actions of convergent individual police responders seeking to stop the shooting. The reliance on individual action is based on their training and culture (Braziel 2016, 60). During the initial response period, SBFD initiated ICS but was unsuccessful in folding the fire ICS into the Police IC structure. Initially this was primarily due to the incompatibility of their radios while later it may be lack of experience with ICS or meaningful training. An ICS-based Unified Command did not develop until law enforcement command needs exceeded their capabilities, and the formal ICS structure was necessary to finally contain this incident.

FORENSIC INVESTIGATION

As federal law enforcement arrived, there developed a number of parallel investigations: San Bernardino Police Department (SBPD) investigating the original murders, San Bernardino County Sheriff's Office (SBCoSO) investigating the officer-involved shooting of the suspects, Bureau of Alcohol, Tobacco, and Firearms investigating bomb manufacturing, Department of Homeland Security investigating the terrorism link, and the FBI investigating the terrorists. As described in "The Purpose of ICS" above, there were few links between LE and fewer with SBFD or San Bernardino County Fire Protection District (SBCoFD).

IC changed fairly often. Without a schedule, these changes occurred unexpectedly to fire ICS participants.

SBFD and SBCoFD fire commanders continued working with LE as the incident evolved from an active shooter to a crime scene then to an extended forensic investigation. The initial resistance from federal LE officers was overcome as they experienced the benefit of logistic support.

BRIEFINGS

Briefings occurred during a communal lunch. It appears the reason the IMT gathered people for lunch was for the purpose of briefings.

LEARNING ICS

Like many of the training programs today, much of the basic training is available locally or online. Due to this mode of delivery, as well as time and financial constraints, training becomes more about structure and listing responsibilities of positions. This can too easily lead one to believe ICS is a bureaucratic and burdensome system that does not lend itself to fast moving and dynamic incidents. In fact, this type of incident is what makes the ICS even more critical to the early dynamic incident.

Due to this fact, members of local, State, and Federal ICS teams spend many hours on established teams, shadowing positions, developing the ability and understanding of functions, and gaining experience interfacing between sections. This also increases expertise in the operations for the various positions along with knowledge of legal responsibilities. Awareness of the structures, supporting communications, common planning, logistics, and interface with all agencies facilitates initiation and effectiveness of ICS for an organization and specific incident.

ESTABLISHING THE INCIDENT MANAGEMENT TEAM

Moving vehicles delayed full deployment of the IMT in the parking lot of the Inland Regional Center (IRC). Ownership of the vehicles was not straightforward. Some vehicles were owned by private citizens working in the IRC buildings and some belonged to various responding LE agencies.

ISSUES WITH ICS

Results that became apparent during the interviews of emergency responders at all levels of the response and from all aspects including Law, Fire, EMS, and Government outlined certain aspects of the response that are important to the discussion.

1. It was noted by Government, Law, Fire, and EMS that self-dispatch to the incident (responding to the incident without being ordered) resulted in difficulties. Operational difficulties included traffic congestion blocking responding Fire and EMS equipment. Some LE units appeared to operate within the incident area without a clear mission or command.
2. Two Command Posts were established, one for Law Enforcement and one for Fire/EMS, leading to further separation of information, situational awareness, and operational decisions.
3. The safe operating environment was questioned by several in their interviews, as many plain clothes officers without standard markings were responding and moving through ongoing operations. This made it impossible to assure scene safety of many of the responding agencies. The Department Operations Center (DOC) for Public Health moved their operations center to a different floor of their building (located 4,000 feet away) because they felt threatened by observing armed unmarked officers moving through their parking area.
4. From the County Government perspective, the physical separation in Incident Command interfered with communications regarding the overall incident.

COMMUNICATION

INTRODUCTION

Communication is a behavior. If someone doesn't want to listen, they won't. If someone doesn't want to talk, they won't. On the other hand, someone who wants to hear will find a way.

The basis of the relationship between information and communication is similar to the relationship between electricity and electrons. Electricity (communication) is the flow of electrons (information) with the purpose to produce voltage (strength) and wattage (power). The flow of electrons makes electricity in the same way the flow of information creates communication. Electricity and communication give strength and the use of that strength gives power. In public safety, we use that power expressing our sense of duty to the public.

"Communication" was a common complaint in this incident as a general statement without the specific information that was needed. Possibly the complaint was about the lack of an accepted structure for communication, as opposed to absent or corrupted transmission of information, or information that is poorly understood.

Operations, however, moved steadily foreword and information flowed without hindrance. Early in the response, there were communication problems between the LE IC and responding Law Enforcement Officers, and also between LE IC and Fire IC in staging. Initiative from the Fire IC mitigated the communication problem to move the fire companies to the triage sites in a timely manner.

In public safety, the ultimate purpose of information and communication is safety. Information, however, entwines with communication making it difficult to distinguish between them. To talk about communication is to talk about information but in public safety it is also to talk about context, the environment, meaning, and safety. Communication does not create safety by the simple transmission or transference of information. Information management is overly broad.

Communication is how we handle information. *Information* is the *measure* of uncertainty about something. When we change uncertainty into certainty we gain or create information (Shannon's Information Theory). The counterpart is to say something that is already certain or known. That is not information.

Context is the information that surrounds the event, binding meaning to the event. In public safety, context carries significant, relevant information. Compare the use of the environment in public safety with its use in medical care where the environment carries far less information. For example, the physician treats pneumonia in the patient, for the most part, regardless of the characteristics of the patient, where the patient lives, or who the family is.

When we treat communication as a part of the environment, then the environment gives context, or meaning, to communication and to how we use information. Communication becomes open and information flows freely. In public safety, information is safety and open and aggressive communication is how we keep each other safe. Prudence and good judgment come from the use of information in context.

Not treating communication as part of the environment changes the way information is handled. Both communication and information can become compartmentalized. Taking information out of its environment removes its meaning, further changing how we use information and how we communicate.

Communication flows in short bursts, particularly in the VUCA-T environment. Communication is neither episodic nor continuous. Short bursts of information can overload a compartmentalized system or energize a system where information flows.

Information surges are characteristic of the VUCA-T environment. These surges can overwhelm the individual or system when the amount of input to a system or person exceeds processing capacity, or a mismatch occurs between information processing and information loads. Karl Weick and Kathleen Sutcliffe (2008) argue that the traditional conception of capacity is a finite amount: too much information and information is lost, missed, or interferes with processing. Overload is physical and perceptual. Weick and Sutcliffe (2008) further argue that *enlargement of understanding* will decrease overload.

Command and control can handle information by compartmentalization or by facilitation of flow through channels. Compartmentalization is susceptible to becoming overloaded with information, but channels do not become overloaded since they stay in constant contact. Leadership style reflects communication style (compartmentalization versus channelization). Leadership *in extremis* favors channelization's flow of information for handling information thus linking leadership to both command and communication in the triad of command, control, and communication.

COMMUNICATION FAILURE

Respondents interviewed for this report were self-critical of their own lapses in communication, but these may be perceived communication lapses and not actual lapses. No respondent identified a situation or problem where they

lacked the information necessary for a decision. Nor did they describe an action they took that failed because they were missing information.

Throughout the interviews, either archived or in person, respondents referred to communication and failure to achieve communication as opposed to failure to communicate or communication failures. Failure to communicate infers one does not attempt to communicate. Communication failure infers there was no means for communication. Criticisms that appear to be about radio communication failures were directed at the radios, not communication. When the law enforcement IC wanted to communicate with the fire department IC, he could not use his radio, so he sent a message to any police officer in the vicinity of fire staging. The mode of communication may fail, but effort to communicate continued. This is not a subtle or nuanced distinction. Throughout the narrative individuals described how they sought to give or find information. They never described someone holding on to information, intentional or otherwise.

Communication, and failure to achieve communication, appeared to be related to expected structures for communication rather than information, who had it, and who needed it. Individuals successfully communicated despite failures of communication structures. This is important for any communication lesson learned; how to embed this drive to communicate, and how to develop the belief that communication has context and its purpose is to channel the flow of information. Once this drive and belief is in place, improvised answers are more effective and information overload does not occur (Weick and Sutcliffe 2008).

COMMUNICATION BETWEEN PEOPLE

Communication occurs at two levels, between individuals *within* an operation and *between* groups.

Communication *face-to-face* occurs within an operation. Individuals appeared to use visual cues, particularly from each other, for effective, rapid, and responsive actions. During a public safety operation, particularly in the fire service, visual communication can have greater fidelity than sound. Distracting noise and voices (particularly cries of casualties) make sound less useful for communication. In a crowded scene, it can be unclear who the person is speaking to, words may have to be repeated, and concrete thinking develops from stress or distraction. Visual cues are detected even from subtle or nuanced movements that can be maintained until visual confirmation. Visual threat cues are particularly sensitive and less susceptible to stimulus fatigue.

Communication by *radio* became a problem between the fire service and law enforcement. The first LE IC could not give information directly to officers responding from other agencies. Incoming officers did not know to drive southbound in the northbound lanes of Waterman Avenue to preserve southbound lanes for fire and EMS nor did they know the location of the command post. Arriving officers acquired little information during their response, which contributed to the aggregating behavior on scene.

Communication between fire and police had to pass between dispatchers. Fire commanders were unable to use police tactical channels. This contributed to the difficulty of fire clearing from staging to triage and finding the law enforcement command post at Waterman and Parkcenter North.

The lack of radio connectivity prevented forming early uniform command. Both commanders understood the urgency, but radio traffic occupied the fire IC and law enforcement activities combined with casualty extraction occupied the police IC. The school police emergency manager’s action, initiative, and creativity physically brought the fire IC to the police IC despite lack of a shared radio frequency and area vehicle congestion. This situation demonstrates the benefit of a company level officer assigned as an aide to the fire commander.

Lack of radio connectivity slowed the move into Triage A but the police IC found a way around the problem by alerting all law enforcement officers to reach a fire unit and tell them to respond to the incident. In keeping with the theme of improvisation, both commanders effectively improvised solutions. Broadcast on SBPD main channel: "Any officer at San Bernardino Ave and Waterman, tell the staged fire units to come in and set up their triage area on Waterman and the entrance of the golf course. I need them there now."

RADIOS

Overall, there was an inability to monitor all radio channels, or at least the other service, effectively. The Battalion Chief, as fire command, had two 800 MHz radios that could be used simultaneously for both fire command and fire tactical channels. The handy talkie was programmed to only receive SBPD's primary dispatch channel. There was no other capability to monitor other channels. This interfered with receiving information early from LE IC and affected his ability to achieve unified command structure early in incident.

The tactical medic acting with SWAT attempted to contact the responding fire command from on scene by using fire's main channel. He wanted to give updates of casualties on scene and casualties moving to fire triage area. His radio traffic was transcribed but for some reason neither the dispatcher nor the Battalion Chief responded. There was nonstop radio traffic making it difficult to communicate, but that seems to have been mostly on law enforcement frequencies during the active search to find the assailants.

The trunk system used by the City of San Bernardino was not full during this incident. The system only allows for so many users at once. For example, San Bernardino City sanitation is on the same trunk as police and fire, so the oddity could have occurred that, had refuse collection used the radio excessively, they could bounce police and fire off the radio.

Numerous respondents stressed that fire *must* hear police during law enforcement operations. The capacity for passive monitoring of radio frequencies would satisfy this without transmissions interfering with radio traffic during a major incident.

COMMUNICATION, INCIDENT COMMANDER

Radio difficulties with communication for the incident commander have been discussed above. Also contributing to this is the model of a single incident commander leading the incident from a vehicle with multiple radios available.

Central to this problem is the amount of information coming to the incident commander and the amount that must go out. Channeling information and facilitating its flow reduces the threat of information overload. The fire and police incident commanders demonstrated this skill quite well. The fire IC working without an aide, however, was approaching an information threshold when task demands impeded achieving face-to-face Uniform Command.

The operational complexity of these events is quite clearly demonstrated by this incident. Woven throughout is the organizational complexity that is more abstract and requires more cognitive function. A second officer, either a fire commander or company officer, would have an essential role in managing the situations. Bringing a single individual in as the action ramps up is far more effective then bringing multiple people in once the activity becomes too complex.

AFTER-ACTION REPORT

There did not appear to be a review of the incident immediately afterward. That is the time when memories are fresher and the drive to improve stronger. A review immediately after would reinforce SBFD as a learning organization, encouraging members to speak up.

The concern of contaminating a more thorough investigation is also legitimate. Discussed elsewhere in this report is the effect of silencing voices to create a dominant account of the incident.

Building an inquiring culture and formal acceptance of the principles of High Reliability Organizing can correct some of these missed directions.

Fire personnel regarding this incident felt it was a failing that they had responded to rumors as though the rumors were real information. Rumors cannot be distinguished, however, during the uncertainty and ambiguity of the event. They can only be established in hindsight.

NEWS MEDIA

The news media has a role in communication to the community for health and safety, covered in another section on news media. The news media can also serve as a conduit to explain the fire department's actions on behalf of the community.

There will be times when the news media would like to interview individual members of the fire department such as occurred following this active shooter incident. These interviews bring a public face to the fire department making the department look more personal and directed toward citizens. Because these situations cannot be predicted, a training session would be less useful than a one-page sheet that describes the nature of these interviews. Such a sheet could be given to the member in preparation for an interview.

NEWS MEDIA

The news is a tool for the first responders. News media can transmit important information in real time from public safety agencies. The news is not there to find a mistake or to show the pain of those suffering. The news media can help keep the public informed, keep them out of the area, and keep them away from the phones calling in. The media is there to be used by public safety agencies for the health and safety of the public.

Some first responders who participated in an incident may have received clearance from their agencies to speak to news reporters. The following section is a guideline for public safety agencies describing the information news reporters will use for these purposes. The material was developed in cooperation with a television news reporter.

DURING THE INCIDENT

The news media, during an incident such an active shooter, acts for the health and safety of the community and becomes a tool for public safety agencies to communicate to the public. What do you want people to know? Where

do you want them to go or where you want them to stay? The news media can relay information in real time to the public because people often watch television or listen to radio and will receive vital information in time to act.

Everything is fluid at the beginning of the incident. There are multiple casualties, it is ongoing, and there are no specifics. Reporters will likely be on the air the second they arrive at an important event. They also have the ability to broadcast on the air from small backpacks. (They intentionally do not show law enforcement or the reporter's location when broadcasting while using a backpack.)

The needs of the news reporter and public safety during an active shooter incident change over time. The information and its reliability also change over time and news reporters understand this. The media knows that the information they report as facts can change. What you believe to be true may change as time goes on, making it important to update your information and report facts as you have them.

News reporters will have several questions:

1. Quickly, they need to know is the incident still active, are people still in danger?
2. What is the location of the incident? What is the immediate area of concern? What should people do?
3. What do you and the other public safety agencies want people to know? You can, in real-time, get information to people. This is a health and safety issue.
4. Are there multiple shooters or only one? Do you have an idea?

Recommendations to public safety officers in speaking to new reporters:

1. Use qualifiers for your first answers. "Because this is still breaking, we cannot give you anything definite."
2. Give advice for the public. "In this area… we do not want people outside. And for people not in that area, we do not want them to go there."
3. Advise the public. "Please do not put yourself at risk and do not put others at risk. Law enforcement and firefighters may have to take action to protect you."
4. What information does the public need to keep themselves and their family safe? In whatever terms that you can give him.
5. What is the area of immediate danger? What to do for those who are in it? What is the known location of area of concern. The area may be bigger at first and shrink later. News reporters understand this.

Television is particularly dependent on pictures. Have the media close enough to hear and see the activity but without being in danger. If the press area is too far away, then some photographers may try to get around that by entering unsafe areas.

ONCE IT IS OVER

Reporters will now ask about the severity and significance of the incident. They will ask for any information that can explain the incident.

1. How severe was the incident? What are the basic numbers? Use rough numbers, there's a difference between two and 200.
2. Who were involved as victims?

The more you can narrow down the injured to those who are most likely injured, then the larger the group is that is not injured. This is important, because you can now exclude others who are not hurt. This significantly reduces the number of people arriving to help relatives and friends.

The sooner this information is broadcast the sooner you can calm the fears of those with family in the area. You will also dampen the likelihood that people will flock to the area to see if their friends or relatives are safe.

The minute you are confident that no other shooters are involved, that is, the shooters are dead or captured, then tell people. Do not prematurely release this information. You can advise that you are closely going over the information.

Considerations

- Give accurate information as quickly as it is confirmed. The media is a means for live communication to the public.
- Do not intentionally *not* tell the truth. Never give information if the purpose is to help you. To hold back information, however, or to give information known to be inaccurate, misleads, and possibly endangers, the public.
- It is okay to say: "I know this is really early and things can change" or "We will give the information we can." It is okay to change the information you provide, as opposed to intentionally waiting until things are clear. Some reporters may undertake unsafe actions for information they believe exists when it is not available.
- If information is proprietary, that is, information belonging to the agency or confidential by necessity, say "I cannot answer that question." The media understand these things are fluid and some information is proprietary. They will respect this answer.
- The news department has a research department that will be looking up the background information. If the open source information is inaccurate, then public safety agencies can provide the correction. For example, in this incident the IRC, an organization serving disabled children and adults, rented space for an outside agency to conduct training. People hearing the address will think their child could be at risk. The news media can correct that misperception.

Social media

- The first eyewitness accounts may be broadcast on social media. Initial eyewitness accounts are notoriously unreliable. The news media will want confirmation of any first reports and any eyewitness accounts.
- Some agencies received notification through social media or their members following the event on social media. The news media can help correct the errors found on social media. The news media wants to make sure the information is accurate, and that inaccurate information is not communicated to the public.
- The first responders, however, may be the ones who give these first eyewitness accounts.

BEYOND IMAGINATION

This incident grew more rapidly than any one person's imagination could keep pace with. Yet the most important parts--neutralizing threats, enhancing survivability, and moving casualties to medical care--were rapidly accomplished. Self-organized, improvising teams entered a chaotic situation to create a more desirable resolution.

Despite the effectiveness and smoothness of the operations for this active shooter incident, participants would identify a discrepancy or disruption they had experienced and bring it to the interviewer's attention. As the interviewers compiled information, many of these incidents that had appeared quite wrong were actually people doing what they were supposed to do.

We offer these vignettes as a reminder that during a large event there are many smaller operations and operations within those operations occurring simultaneously and overlapping.

TOO MANY COPS

One observation made by many in the fire department and EMS was the large number of law enforcement officers and the resulting parking problem and loss of drivable highway. The fire department routinely maintains a route to come into the incident and to leave the incident for the purposes of bringing in equipment they may need or for a rapid escape.

Over the radio, arriving officers began asking for the CP location. No one had taken command of the incident. Different agency officers/deputies began flowing into the scene. The SWAT Commander tried to stop the influx of officers and gain control. He wanted to control ingress of personnel. He made it to the doors of the crime scene and then turned around, moving toward Waterman and Parkcenter to set up the CP.

The SWAT Commander could see that Waterman Avenue was becoming congested by incoming police units. He requested the police dispatcher to have all incoming police resources arriving from the north to drive southbound but in the northbound lanes. This would leave the southbound lanes clear for fire and EMS units. This message was not broadcast.

The LE incident commander notified the dispatcher to have all responding outside law enforcement officers switch to the SBPD frequency to help with communications. The dispatchers were not able to transmit the message in time, so the IC was becoming inundated at the command post with officers looking for an assignment. He then began assigning teams of officers for jobs such as patient care in the conference room and force protection for the fire department at Triage A. It was not so much that the law enforcement officers were out of control, it was that they had arrived without information.

In this incident, it appeared to SBFD personnel that no one was organizing the law enforcement officers. Interview transcripts reflect that the LE incident commander, while occupied with life or death responsibilities, also maintained focus on supporting the fire department and giving assignments to the numerous arriving law enforcement officers.

This was a problem of a large, rapid response of police officers to an incident with one commanding officer who was trying to affect a search for the suspects and rescue of the victims.

- Officers would arrive and stand around. Some would check in but not leave information where they could be found.
- Law enforcement officers were unfamiliar with, and inexperienced in, ICS. They did not know the importance of checking in.

- The emphasis Law enforcement places on individual mutual support and initiative led them to respond when other officers were fired upon. Their response left firefighters unprotected.

REACHING THE FIRE DEPARTMENT

Fire companies were staged at Waterman and Orange Show Road waiting to drive into Triage A. They observed a law enforcement officer at the triage site waving and shouting, “Come on. Come on, they need you in there.” They could not understand why the orders to move into the triage site would come from a frantically yelling law enforcement officer rather than the law enforcement incident commander.

The Battalion Chief found it difficult to maintain his discipline and not send FD units to scene. His first responsibility however, was to keep his people safe, and they had not been officially cleared. Thoughts of the Columbine shooting incident were in the back of his mind as they waited for clearance, he knew that seconds would count. The CHP officer was wanting them down there urgently, continuing to shout, but no one with the fire department broke the perimeter from the north or south until they had been officially cleared and he gave the order to move.

We tried to coordinate with the BC: “are we cleared in or not?” There was a delay between what we heard on the PD radio (“Get fire in”) and FD’s dispatch (“stage”). Dispatch was waiting for the BC to clear FD to move in and we were eventually cleared.

When the LE IC could not reach FD on radio, he called every officer on the police radio to tell FD they are clear to go in.

HELO CREW

While en-route they hear radio traffic of “20 or more victims.” They orbited the scene a couple of times. They could see the city fire command post and hear PD calling for fire medics to enter. They knew that Fire Command did not hear the PD request.

SCHOOL PD

He parked next to SBFD BC. He had both FD and PD radios. He was hearing both and getting more info than the BC. He heard SWAT Commander “We’re responding.” SBFD BC hadn’t gotten the word that FD needs to roll in and stage at location for the Treatment Area. He told the BC that Fire needed to get into the scene at Waterman and Parkcenter. He drove the BC to Walker’s vehicle—CP location. His intention was to get FD IC to LE IC together so there was no gap.

DISCIPLINE AND INITIATIVE

"Their discipline and training is to stage, and not simply drive in without receiving an assignment."

Battalion Chief

" A second engine came in behind and also staged."

Fire Captain

As noted in the previous section, a law enforcement officer was near staging on the perimeter of the incident. He was jumping up and down, screaming and waving, "Come here, we need you!" over and over. The Battalion Chief found it difficult to maintain his discipline and not send FD units to the scene, but they had not been officially cleared. *His job is to keep his people safe* while knowing they were needed badly. He believes the Columbine shooting incident was in back of mind, he knew that seconds count and the CHP officer was wanting them down there, shouting, "Come on, come on!" Nobody with the FD broke the perimeter from the north or south until he gave the order to move.

Not long into the incident the SWAT Medic approached and requested the Captain and crew to come with him to IRC building [in the warm zone, the room had not been fully cleared]. The SWAT medic came up and said, "I need triage to be done here. Bring your gear. We may have 20 more victims." He coordinated with the SWAT medic to provide security for his crew on their approach.

TRIAGE SITE SAFETY: INTO THE WARM ZONE

Police IC

- He cleared fire into scene (to the Treatment Area) on Waterman and Parkcenter. He waited a long time. He could see fire was not moving. He didn't want a lot of self-transports like Aurora. He prepared for a battle with FD because he didn't think they'd want to move that close to the scene. "Would fire move that close to the incident, basically in the warm zone?" He wondered.

SBFD BC #2

- "We are willing to go in even if dangerous to keep people from dying, after cops create minimal security. They do not want to be a FD that stages and waits to be safe."
- For the next one, they need to know there is a risk. "I could be at risk, but I can save the person."

SBFD Engine Captain

- The captain was trying to coordinate with the BC: "Are we cleared in or not?" He kept asking himself. There was a great sense of urgency to move to triage.

SBFD BC #1

- "I was seeing a CHP officer doing traffic control at the intersection waving his arms and hands at Medic Truck 221 to move forward. He was yelling "come on, come on they need you in there."
- At the same time a fire officer came over the command channel, he was hearing traffic over PD's channel 1 that the LE IC was requesting fire to respond in to the scene." I attempted to confirm this by going through our dispatch center but was unable to get a response."

- “With what I was hearing and what I was seeing, I broadcasted to all units to respond to the entrance of the golf course and Waterman and assume their pre-designated assignments.”

The LE IC

- “Set up at the mouth of the golf course. I will get guys to protect you ASAP.”
- He was pleasantly surprised when FD moved to the location he designated.

Fire set up the Triage Site at 11:15 a.m.

GAP BETWEEN FIRE AND POLICE COMMAND

LE IC

Would have been nice to have a PD-FD command at the onset of incident. Don’t know how long it took fire to integrate into command structure, but it was too long. Had a meaningful discussion with FD IC but would have been nice to tie together in the same physical location to have on-going discussions. FD IC was with School PD, that kept FD and PD from joining up.

FIRE IC AND UNIFIED COMMAND

There was so much radio traffic on my tactical channel that I had to address, I never had the opportunity to break and verify exactly where PD had the command post set up. Shortly after I took a position on Waterman Avenue, a School PD officer pulled up next to me. He stated to me he had been listening to PD's incident commander on their tactical channel and was requesting that I come to their command post. At that time, I couldn't leave my position without compromising command and control of my objectives due to the amount of radio traffic I had to manage. He offered to take me over to the PD's command post in his vehicle which allowed me to maintain radio communications as well as monitor all of PD's radio traffic.

We moved my command post 1/4 block amongst a sea of police units and had a face-to-face with LE IC. At this point we still had not established a formal unified command. I still wasn't able to break away from the radio as units were coming in staging and requesting additional resources.

SCHOOL PD

Later he heard SWAT requesting SBFD to move to the triage/ treatment site. He knew that SBFD had not received that message. His intention was to get FD IC to LE IC together so there would be no gap in command. He parked next to the SBFD BC to advise the battalion chief and assist in communications and formation of a Unified Command. He drove the BC to the SWAT Commander’s vehicle which was the Command Post.

UNEXPECTED HELICOPTER TRANSPORT

A trauma center received two patients by helicopter. They had not been notified of the patients. They were also concerned that their ReddiNet message showed the helicopter was ordered to fly to a private, non-trauma hospital and the paramedic on board appeared to have redirected the flight to the trauma center.

There was a pause in the flow of ambulances coming to Triage A. The transportation manager quickly took advantage of this lull and selected two patients to be transported by the Sheriff's Air Rescue helicopter. The purpose for this transfer was 1) to move some of the patients to trauma centers away from the two local trauma centers and 2) take advantage of the pause to continue moving patients. There was no demand for the helicopter to fly to a private hospital. The helicopter did not notify the trauma center because the flight was too short. Instead, they requested their dispatcher to notify the trauma center, but the dispatcher was preoccupied with activities surrounding the shooting.

WANDERING EVACUEES

One paramedic complained about the creation of a second triage site while he was trying to manage the one he was working at.

The other site was Triage B, a secondary triage site for the possible injured people evacuating from an office building.

The call came for an ambulance with a field supervisor to respond to a home north of Triage A. Another supervisor heard the request, but it did not go through. The dispatcher did not follow the process to obtain an ambulance.

The home was an abandoned house used for Triage B. It was selected for the metal fence that could be used for protection if a sniper was present. The location would also keep the north building people away from triage A if they had to evacuate any patients from North building.

LESS SERIOUS PATIENT TRANSPORTED FIRST

Ambulance crew was prepared to transport a seriously wounded patient shot in the chest. Instead they were given a less critical patient with multiple wounds in the extremities.

At one point a patient was going to get thoracentesis but a patient with multiple gun shots had an IV and was ready to go. Ambulances were backed up. The medic did not want to waste time, so he sent the patient with multiple gunshot wounds who was less sick, so he could complete the thoracentesis for the patient with the chest problem. Certain treatments could delay transport, but the time of delay was not significant in comparison to completion of treatment.

HOW BIG IS THIS?

I received a lot of negative feedback from nurses when I was completing my paperwork. The nurses thought that the two minor shooting victims should've been taken to a trauma center and not their facility.

I advised them that in an MCI, this is exactly what should happen, the trauma or specialty hospitals should not be inundated with minor patients simply because of the type of trauma. It seems as though there are always people who don't understand.

AMBULANCE SELF-DISPATCH

One company officer complained that a private ambulance service self-dispatched several ambulances to the scene which caused confusion within the system.

The EMS Authority had called Symon's Ambulance and requested two strike teams to respond.

INJURY DISCREPANCY

Field to emergency department

Vital sign records for all patients within the range accepted as normal. Patients may have more than one discrepancy.

Level of triage

"Immediate" or "Delayed"

- Severe bleeding wound to leg transported as "immediate"; discharged from emergency department with graze wound.
- Superficial facial injury, transported "immediate"; surgical repair of facial fracture.
- Back [chest] with graze wound; in emergency department found to have pneumothorax, received chest tube and endotracheal intubation.
- Abdomen with shrapnel; in emergency department to operating room for significant abdominal injuries.
- Suspected hypovolemia, fluid bolus 300 mL normal saline, no response; in emergency department received blood transfusion.

"Minor" level

Minor, home without care, transported by private vehicle; emergency department, chest radiograph showed shrapnel in chest.

REFERENCES

American College of Surgeons Committee on Trauma. 2012. *Advanced trauma life support: Student manual.* 9th ed. Chicago, IL.: American College of Surgeons.

Arnsten, Amy F. 2009. "Stress Signaling pathways that Impair prefrontal cortex structure and function." *Nature Reviews Neuroscience* 10 (6): 410–22.

Ashby W.R. (1958) "Requisite variety and its implications for the control of complex systems." *Cybernetica* 1 (2):83-99.

Baran, Benjamin E., and Cliff W. Scott. 2010. "Organizing ambiguity: A grounded theory of leadership and sensemaking within dangerous contexts." *Military Psychology* 22 (S1): S42.

Barton, Michelle A., Kathleen M. Sutcliffe, Timothy J. Vogus, and Theodore DeWitt. 2015. "Performing under uncertainty: Contextualized engagement in wildland firefighting." *Journal of Contingencies and Crisis Management* 23 (2): 74-83.

Benner, Patricia. 1982. "From Novice to Expert." American Journal of Nursing 82 (3):402-407.

Bonanno, George A. 2004. "Loss, Trauma, and Human Resilience: Have We Underestimated the Human Capacity to Thrive After Extremely Aversive Events?" *American Psychologist* 59: 20–8.

Bonanno, George A., and Charles L. Burton. 2013. "Regulatory Flexibility: An Individual Differences Perspective on Coping and Emotion Regulation." *Perspectives on Psychological Science* 8 (6): 591–612.

Boonstra, Rudy. 2013. "The Ecology of Stress: A Marriage of Disciplines." *Functional Ecology* 27 (1): 7–10.

Braziel, Rick, Frank Straub, George Watson, and Rod Hoops. 2016. *Bringing Calm to Chaos: A Critical Incident Review of the San Bernardino Public Safety Response to the December 2, 2015, Terrorist Shooting Incident at the Inland Regional Center*. Critical Response Initiative. Washington, DC: Office of Community Oriented Policing Services.

Browner, Bruce D., Jesse B. Jupiter, Christian Krettek, and Paul A. Anderson. 2014. *Skeletal Trauma E-Book*. Philadelphia, PA: Elsevier Health Sciences.

Calhoun, Lawrence G., and Richard G. Tedeschi. 2014. "The Foundation of Posttraumatic Growth: An Expanded Framework", In *Handbook of Posttraumatic Growth*: Research and Practice, edited by Lawrence G. Calhoun and Richard G. Tedeschi, 1-23. New York, NY: Routledge.

Campbell, Joseph. 1976. *The Masks of God: Occidental Mythology*. Vol. 3. New York, NY: Penguin.

Cromwell, Howard Casey, and Jaak Panksepp. 2011. "Rethinking the cognitive revolution from a neural perspective: how overuse/misuse of the term 'cognition' and the neglect of affective controls in behavioral neuroscience could be delaying progress in understanding the BrainMind." *Neuroscience & Biobehavioral Reviews* 35 (9): 2026-2035.

Dieterly, Duncan L. 1980. "Problem Solving and Decisionmaking: An Integration." *NASA Technical Memorandum 81191*. Moffett Field, California: NASA Ames Research Center.

Dixon, Deirdre P., Michael Weeks, Richard Boland Jr, and Sheri Perelli. 2016. "Making Sense When It Matters Most: An Exploratory Study of Leadership *In Extremis." Journal of Leadership and Organizational Studi*es. 24 (3): 294-317.

Dreyfus, H. L. 1997. "Intuitive, deliberative, and calculative models of expert performance." In C. E. Zsambok & G. Klein (Eds.), *Naturalistic decision making,*17-28. New York, NY: Lawrence Erlbaum.

Dreyfus, Stuart E. 2004. "The Five-Stage Model of Adult Skill Acquisition." *Bulletin of Science, Technology and Society* 24 (3): 177–81.

Elmes, Michael and Bob Frame. 2008. “Into hot air: A critical perspective on Everest.” *Human Relations* 61(2): 213-241.

Emson, Harry E. 1987. “Health, disease and illness: matters for definition.” *Canadian Medical Association Journal*. 136(8): 811–813.

Fraher, Amy L., Layla Jane Branicki, and Keith Grint. 2017.” Mindfulness in Action: Discovering How US Navy Seals Build Capacity for Mindfulness in High-Reliability Organizations (HROs).” *Academy of Management Discoveries* 3(3): 239-261.

Greenberg, Jeff, and Jamie Arndt. 2011. “Terror Management Theory.” *Handbook of Theories of Social Psychology* 1: 398–415.

Hall, Edward T. 1959. *The Silent Language*. New York: Doubleday.

Heggie, Vanessa. 2013. "Experimental physiology, Everest and oxygen: from the ghastly kitchens to the gasping lung." *The British Journal for the History of Science*. 46 (1): 123-147.

Holcomb, John B., Lynn G. Stansbury, Howard R. Champion, Charles Wade, and Ronald F. Bellamy. 2006. "Understanding combat casualty care statistics." *Journal of Trauma and Acute Care Surgery* 60 (2): 397-401.

Ioannidis, John PA. 2016. "Why most clinical research is not useful." *PLoS medicine* 13(6): e1002049.

Jacobs, L. M., A. Eastman, N. Mcswain, F. K. Butler, M. Rotondo, J. Sinclair, D. S. Wade, W. R. Fabbri, and Joint Committee to Create a National Policy to Enhance Survivability from Mass Casualty Shooting Events. 2015. "Improving Survival from Active Shooter Events: The Hartford Consensus." *Bulletin of the American College of Surgeons*100 (1 Suppl): 32-34.

Kelly, Joseph F., Amber E. Ritenour, Daniel F. McLaughlin, Karen A. Bagg, Amy N. Apodaca, Craig T. Mallak, Lisa Pearse et al. 2008. "Injury severity and causes of death from Operation Iraqi Freedom and Operation Enduring Freedom: 2003–2004 versus 2006." *Journal of Trauma and Acute Care Surgery* 64 (2): S21-S27.

Kolditz, T. A., & Brazil, D. M. 2005.” Authentic leadership in *in extremis* settings.” In W. L. Gardener, B. J. Avolio & F. O. Walumbwa (Eds.), *Authentic leadership theory and practice: Origins, effects and development* (Vol. 3). Oxford, UK: Elsevier.

Kolditz, Thomas A. 2006. "Research in *in extremis* settings: expanding the critique of ‘why they fight’." *Armed Forces & Society* 32 (4): 655-658.

Kolditz, Thomas A. 2010. *In extremis leadership: Leading as if your life depended on it*. Vol. 107. Hoboken, NJ: John Wiley & Sons.

Kotwal, Russ S., Harold R. Montgomery, Bari M. Kotwal, Howard R. Champion, Frank K. Butler, Robert L. Mabry, Jeffrey S. Cain, Lorne H. Blackbourne, Kathy K. Mechler, and John B. Holcomb. 2011."Eliminating preventable death on the battlefield." *Archives of surgery* 146 (12): 1350-1358.

Langer, Ellen. 2014. *Mindfulness, the 25th Anniversary Edition*. Perseus Books: Philadelphia, PA.

LeDoux, Joseph E. 2014. “Coming to Terms with Fear.” *Proceedings of the National Academy of Sciences* 111 (8): 2871–8.

LeDoux, Joseph E., and Daniel S. Pine. 2016. “Using Neuroscience to Help Understand Fear and Anxiety: A Two-System Framework.” *American Journal of Psychiatry* 173 (11): 1083–93.

LePore, Stephen J., and Tracey A. Revenson. “Resilience and Posttraumatic Growth: Recovery, Resistance, and Reconfiguration.” In *Handbook of Posttraumatic Growth: Research and Practice*, edited by Lawrence G. Calhoun and Richard G. Tedeschi, 24–46. Routledge: New York.

Mechem, C. Crawford, Richard Bossert, and Christopher Baldini. 2015. "Rapid assessment medical support (RAMS) for active shooter incidents." *Prehospital emergency care* 19 (2): 213-217.

Morris, David J. 2015. *The Evil Hours: A Biography of Post-Traumatic Stress Disorder*. New York: Houghton Mifflin Harcourt.

Moynihan, Donald P. 2009. “From intercrisis to intracrisis learning.” *Journal of Contingencies and Crisis Management* 17(3): 189-198.

Neimeyer Robert. 2014. “Re-Storying Loss: Fostering Growth in the Posttraumatic Narrative.” *In Handbook of Posttraumatic Growth: Research and Practice*, edited by L. G. Calhoun and R. G. Tedeschi, 68–80. New York, New York: Routledge.

Orwell, George. 2008. “Shooting elephant.” In *Facing Unpleasant Facts: Narrative essays* compiled by George Packer, 29-37. Boston, MA: Mariner Books.

Palmer, Noel F.; Hannah, Sean T.; and Sosnowik, Daniel E. 2011. “Leader Development for Dangerous Contexts.” US Army Research. 350. http://digitalcommons.unl.edu/usarmyresearch/350 accessed November 1, 2017

Park, Crystal L. 2010. “Making Sense of the Meaning Literature: An Integrative Review of Meaning Making and Its Effects on Adjustment to Stressful Life Events.” Psychological Bulletin 136 (2): 257-301.

Rasmussen, Jens. 1980. “What Can Be Learned from Human Error Reports?” in Duncan, K.D., Gruneberg, M.M. and Wallis, D. (eds), *Changes in Working Life*, 97-113. New York, NY: Wiley.

Rasmussen. 1983. “Skills, Rules, and Knowledge: Signals, Signs, and Symbols, and Other Distinctions in Human Performance Models.” *IEEE Transactions on Systems, Man, and Cybernetics*, SMC-13 (3): 257–66.

Reason, James. 2004 "Beyond the organizational accident: the need for “error wisdom” on the frontline." Quality and Safety in Health Care 13 (2): ii28-ii33.

Roe, Emery, and Paul R. Schulman. 2015. "Comparing Emergency Response Infrastructure to Other Critical Infrastructures in the California Bay-Delta of the United States: A Research Note on Inter-Infrastructural Differences in Reliability Management." *Journal of Contingencies and Crisis Management* 23 (4): 193-200.

Russell, Bertrand. 1910. Knowledge by acquaintance and knowledge by description. In *Proceedings of the Aristotelian Society*.11: 108-128. London, UK: Aristotelian Society, Wiley-Blackwell.

Silver, Nate. 2012. *The signal and the noise: why so many predictions fail--but some don't*. NY: Penguin Books.

Smith, Edward Reed, Geoff Shapiro, and Babak Sarani. 2016. "The profile of wounding in civilian public mass shooting fatalities." *Journal of Trauma and Acute Care Surgery* 81 (1): 86-92.

Taleb, Nassim Nicholas. 2007. *The black swan: the impact of the highly improbable.* NY: Random House.

Tedeschi, Richard G., and Lawrence G. Calhoun. 2004. “Posttraumatic Growth: Conceptual Foundations and Empirical Evidence.” *Psychological Inquiry* 15 (1): 1–18.

Tempest, Sue, Ken Starkey, and Christine Ennew. 2007. "In the Death Zone: A study of limits in the 1996 Mount Everest disaster." *Human Relations* 60 (7): 1039-1064.

TriData Division, System Planning Corporation. 2014. *Aurora Theater 16 Theater Shooting: After Action Report for the City of Aurora, Colorado*. Aurora, CO: City of Aurora.

Van Stralen, Daved, Spencer Byrum and Bahadir Inozu. 2017. “High Reliability for a Highly Unreliable World: Preparing for Code Blue through Daily Operations in Healthcare.” North Charleston, SC: CreatSpace Publishing.

Weick. 1993. “The collapse of sensemaking in organizations”: The Mann Gulch disaster. *Administrative science quarterly* 38(4): 628-652.

Weick. 1998. Introductory essay: “Improvisation as a Mindset for Organizational Analysis.” *Organization Science* 9 (5): 543–55.

Weick. 2010. "Reflections on enacted sensemaking in the Bhopal disaster." *Journal of Management Studies* 47 (3): 537-550.

Weick. 2011. "Organizing for Transient Reliability: The Production of Dynamic Non-Events." *Journal of Contingencies and Crisis Management* 19 (1): 21–7.

Weick, Karl E., and Kathleen M. Sutcliffe. 2011. *Managing the unexpected: Resilient performance in an age of uncertainty*. Hoboken, NJ: John Wiley & Sons.

Weick, Karl E. 1979. "Enactment and Organizing" in *The Social Psychology of Organizing* (Topics in Social Psychology Series), 147-169. NY: McGraw-Hill.

Weick, Karl E., and Karlene H. Roberts. 1993. "Collective mind in organizations: Heedful interrelating on flight decks." *Administrative science quarterly* 38 (3): 357-381.

Weick, Karl E., Kathleen M. Sutcliffe, and David Obstfeld. 2005. "Organizing and the process of sensemaking." *Organization science* 16(4): 409-421.

Williams, George T., and Jeff Martin. 1999. "Responding to the active shooter." *Law and Order*. 47 (10): 71-76.

Wolfberg, Adrian. 2006. "Full-Spectrum Analysis: A New Way of Thinking for a New World." *Military Review* 86 (4): 35-42.
